# A web server for analysis, comparison and prediction of protein ligand binding sites

**DOI:** 10.1186/s13062-016-0118-5

**Published:** 2016-03-25

**Authors:** Harinder Singh, Hemant Kumar Srivastava, Gajendra P. S. Raghava

**Affiliations:** Bioinformatics Centre, CSIR-Institute of Microbial Technology, Chandigarh, 160036 India; http://www.imtech.res.in/raghava/

**Keywords:** Ligand-amino acid interaction analysis, Two-sample logo, Motif analysis, Propensity-based analysis, Amino acid composition based analysis, Physicochemical property-based analysis

## Abstract

**Background:**

One of the major challenges in the field of system biology is to understand the interaction between a wide range of proteins and ligands. In the past, methods have been developed for predicting binding sites in a protein for a limited number of ligands.

**Results:**

In order to address this problem, we developed a web server named ‘LPIcom’ to facilitate users in understanding protein-ligand interaction. Analysis, comparison and prediction modules are available in the “LPIcom’ server to predict protein-ligand interacting residues for 824 ligands. Each ligand must have at least 30 protein binding sites in PDB. Analysis module of the server can identify residues preferred in interaction and binding motif for a given ligand; for example residues glycine, lysine and arginine are preferred in ATP binding sites. Comparison module of the server allows comparing protein-binding sites of multiple ligands to understand the similarity between ligands based on their binding site. This module indicates that ATP, ADP and GTP ligands are in the same cluster and thus their binding sites or interacting residues exhibit a high level of similarity. Propensity-based prediction module has been developed for predicting ligand-interacting residues in a protein for more than 800 ligands. In addition, a number of web-based tools have been integrated to facilitate users in creating web logo and two-sample between ligand interacting and non-interacting residues.

**Conclusions:**

In summary, this manuscript presents a web-server for analysis of ligand interacting residue. This server is available for public use from URL http://crdd.osdd.net/raghava/lpicom.

**Reviewers:**

This article was reviewed by Prof Michael Gromiha, Prof Vladimir Poroikov and Prof Zlatko Trajanoski.

**Electronic supplementary material:**

The online version of this article (doi:10.1186/s13062-016-0118-5) contains supplementary material, which is available to authorized users.

## Background

Ligands play a variety of roles in the regulation and expression of proteins. Currently, PDB has thousands of ligands and the majority of them bound non-covalently to various proteins. The non-covalent ligand binding occurs by intermolecular forces like hydrogen bonds, ionic bonds, hydrophobic-hydrophobic interaction, van der Waals forces, etc. 3D shape of the protein gets altered as a result of the ligand binding. These changes in the conformational state of the protein may activate or inhibit some specific function of the protein. Various methods have been developed to predict the binding affinity of ligands [1–9]. Many databases are also developed to summarize binding affinity of a diverse class of ligands [10, 11] or specific class of ligands [12, 13].

Ligands have high or low binding with specific amino acids depending on various factors (e.g. shape, charge, surface area). ATP has significantly higher interaction with glycine and least interaction with leucine [14]. Various studies have been performed to understand the binding behaviour of ligands with the amino acids in a protein. Many machine learning methods have also been developed to predict the preference of interacting and non-interacting amino acids with various ligands [15–24].

However, binding preference analysis between different ligands and protein was not carried out on a large dataset. Considering this, we performed a rigorous study to understand the binding behaviour of various ligands with different amino acids. This information can be used to either enhance or diminish the binding strength of the given ligand by mutating unfavourable residue with preferred residue at the site of binding. In addition, we developed a web-based platform for the analysis of amino acid preference for all the ligand present in PDB.

## Results and Discussion

### Clustering of nucleotides based on their binding sites

The nucleotides are clustered to understand similarity or dissimilarity in their binding sites. In this study only major nucleotides (e.g., adenine, guanine, cytosine, uracil, thymine monophosphate) are clustered based on residues preferred in their binding sites.

### Propensity-based clustering

**Fig. 1:**
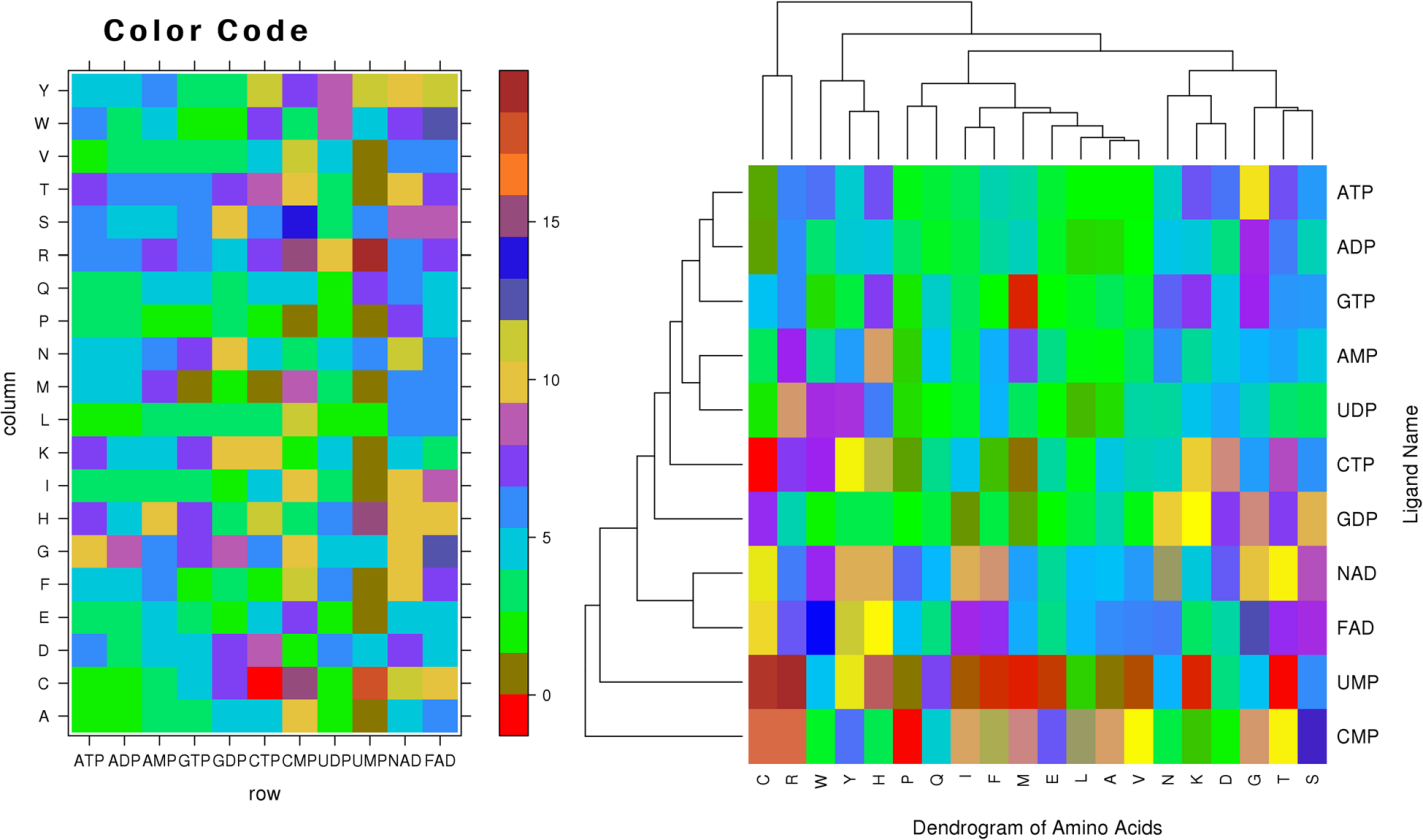
Shows residue-wise propensity score for different nucleotides (left) and clustering of nucleotides based on propensity score (right)

The propensity score of nucleotides interacting residues (${\mathbf{RP}}_{\mathbf{i}}\Big)$ is calculated using equation 3 and the propensity-based Euclidean distance (**PED**_**p,q**_) between nucleotides is calculated using equation 5. The **PED** between each pair of nucleotides is used to construct a distance matrix, which was further used for clustering these nucleotides. Figure 1 depicts the propensity score based clustering of all the nucleotides. The propensity score of GMP, CDP and UTP ligands are negligible and hence these ligands are not included in the analysis. We defined preference of a residue in ligand binding site based on its propensity score, if score of a residue is lower than 5 than we called it low preferred residue. Similarly, we called a residue moderate if it has propensity score between 5 to 12; high if score is more than 12. As shown in Fig. 1, for most of nucleotides propensity score for different type of residues is low or moderate. It is clear from Fig. 1 that ATP and ADP nucleotides are highly similar in term of residue preferred in their binding sites as Euclidean distance is minimum. Similarly, AMP and UDP nucleotides are clustered together; while CMP nucleotide is out of cluster. The NAD and FAD nucleotides binding/interacting residues are also similar as they fall in same cluster.

Figure 1 indicates the low propensity score of W,Y,H,Q,N,K,D,L and F amino acids for CMP nucleotide. Based on propensity score one may conclude that CMP has a strong preference for amino acid S,C,R,I and G amino acids. Similarly, UDP binding sites are dominated by C,R,H,I,E and V amino acids, as the propensity of these residues is high for UDP. The interaction of CTP with most of the amino acids also falls in the low category. The interaction of GTP ligand with H,K and G amino acids falls in the moderate category while amino acid M shows negligible interaction with this ligand. Rest 16 amino acids show low interaction with GTP ligand. Interestingly ADP ligand shows low interaction with most of the amino acids except G and T where moderate interaction is observed. W,H,K,D,G and T amino acids show moderate interaction with ATP ligand and rest 14 amino acids show low interaction with this ligand. Amino acids show similarity generally on the basis of their category e.g. charged amino acids (K and D), hydrophobic amino acids (L, A and V) and polar amino acids (T and S) show similarity up to some extent. Clearly, there is no similarity between C & I, W & V etc. amino acids.

### Clustering of nucleotides using physicochemical property-based

**Fig. 2:**
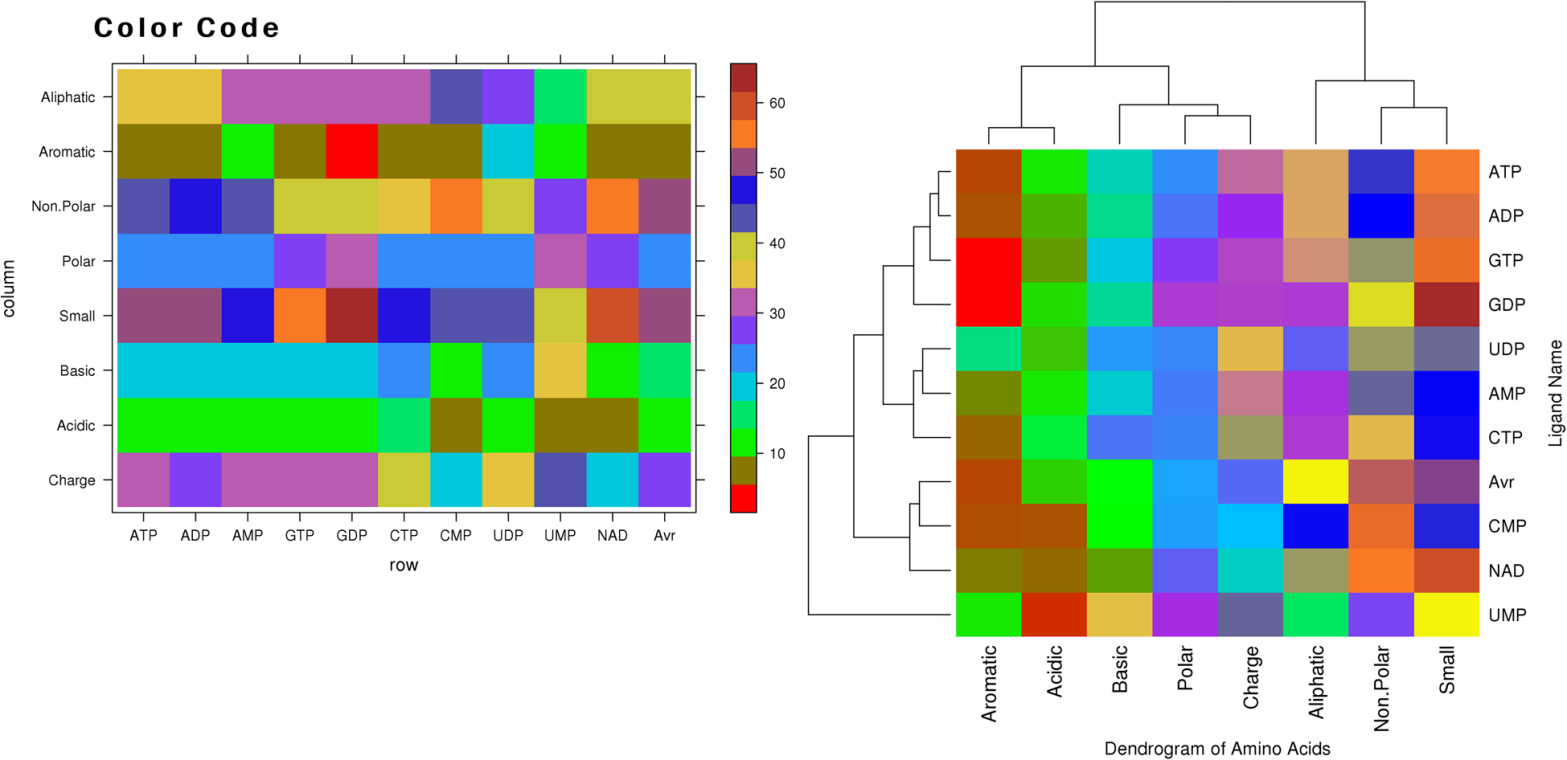
Show property based residue composition of different nucleotides binding sites (left) and clustering of nucleotides based on the physicochemical properties of ligand interacting amino acids

The physicochemical property based composition of nucleotides binding/interacting residues, ${\mathbf{PC}}_{\mathbf{i}}$ is calculated using equation 6. The physicochemical composition based Euclidean distance (**PCED**_**p,q**_) between nucleotides is calculated using equation 7. The **PCED**_**p,q**_ of each nucleotides is used to construct a distance matrix, which was further used for clustering. Figure 2 shows the clustering of different nucleotides based on physicochemical properties of residues in their binding sites or interacting residues. In this ATP and ADP nucleotides falls in same cluster, it means ATP and ADP interacting residues have similar physicochemical properties. Similar trend was observed for GTP and GDP binding or interacting residues. The next group of nucleotides consist of UDP, AMP and CTP. The UMP nucleotide again occurs as isolated in the overall cluster of nucleotides. As expected the NAD and FAD nucleotides occur in the same group and are most similar with CMP and AVR (average of all ligand interacting amino acids in PDB) ligands. Aromatic and acidic amino acids show similar interactions and form a single group. The interaction of polar and charged amino acids is also similar to each other followed by basic group of amino acids. Non-polar and small amino acids show similarity in the interaction up to some extent. Aliphatic amino acids have similarity with the group of non-polar, small amino acids and are least similar to all other groups. UMP ligand shows low interaction with aromatic and aliphatic amino acids, strong interaction with acidic amino acids and moderate interaction with other groups.

### Clustering of carbohydrates based on their binding sites

**Fig. 3:**
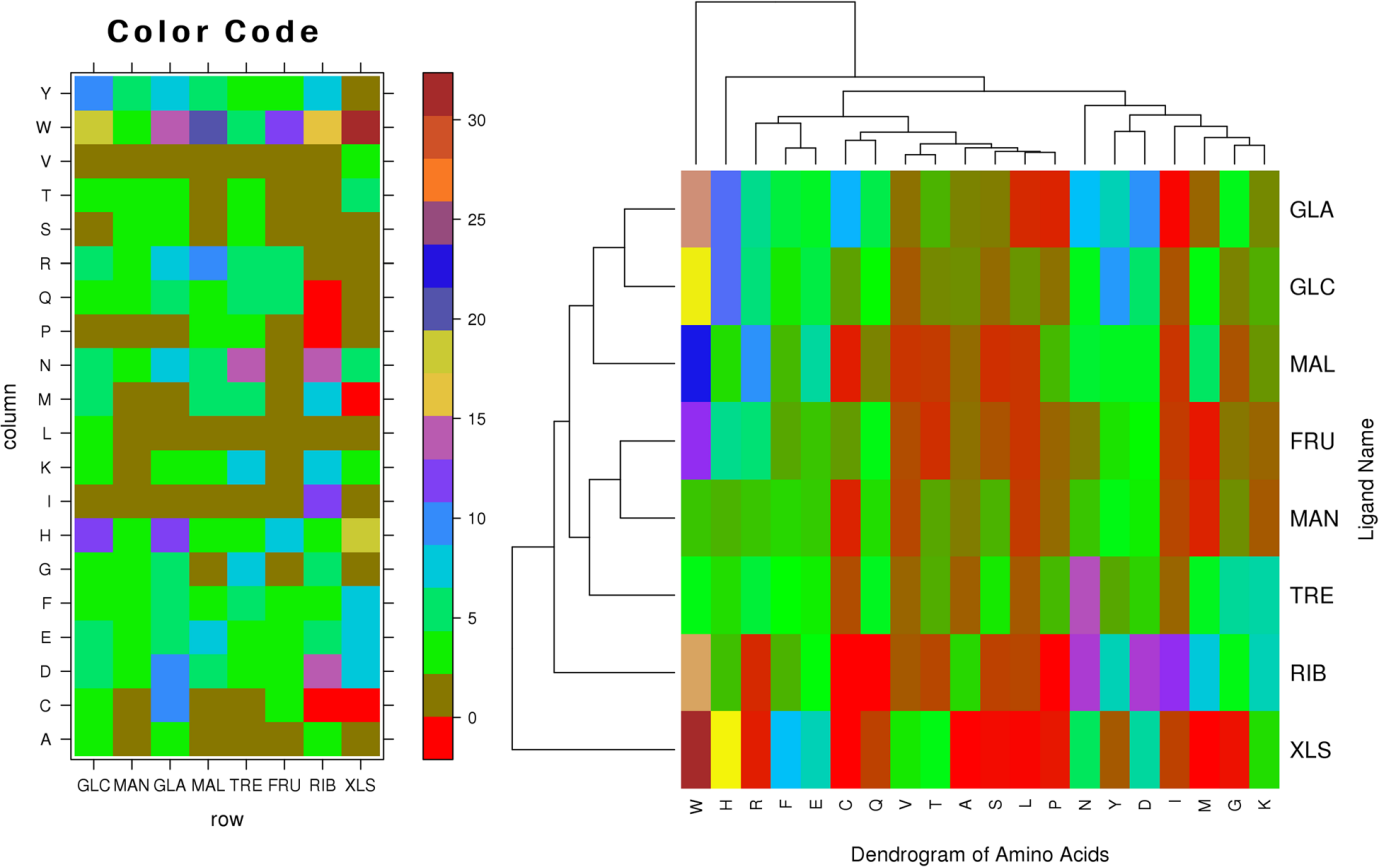
Shows residue-wise propensity score for different carbohydrates (left) and clustering of carbohydrates based on propensity score (right)

The propensity score of carbohydrates binding or interacting residues (${\mathbf{RP}}_{\mathbf{i}}\Big)$ is calculated using equation 3. In order to compute similarity/distance between different carbohydrates, we compute propensity based Euclidean distance (**PED**_**p,q**_) between carbohydrates using equation 5. The **PED** between all pair of carbohydrate was compute for distance matrix, which was further used for clustering. Figure 3 shows the clustering of carbohydrates based on the propensity score of interacting amino acids. The interacting region of GLC and GLA carbohydrates are similar and occur in the same group. Both of these carbohydrates interacting regions are similar to the MAL carbohydrate. The MAN and FRU carbohydrates also show similar interactions and form the same group. Interactions of TRE carbohydrate with the group of FRU, MAN group are also similar up to some extent. The remaining two carbohydrates RIB and XLS occur as isolated in the overall cluster of carbohydrates. The F and T amino acids occur in the same group (F-T). C-Q, V-T, A-S-L-P, Y-D and M-G-K amino acids show similarity in the interaction with various carbohydrates. On the other hand, the interaction behaviour of W and C amino acids are different as clear from Fig. 3.

Similarly, GLA-GLC-MAL ligands and FRU-MAN-TRE ligands show very similar interactions with various amino acids. There is no similarity between the interaction behaviour of XLS and MAN ligands. XLS shows negligible interaction with A,S,L,P,M and G amino acids and strong interaction with W,Q,Y and I amino acids. Other amino acids show low or moderate interaction with XLS ligand. RIB shows a strong interaction with V,T,S and L-amino acids and negligible interaction with C,Q and P amino acids. TRE shows a strong interaction with C,L,A and I amino acids while other amino acids show low or moderate interaction with this ligand. MAN and FRU ligands show low interaction with most of the amino acids while MAL, GLC and GLA ligands show strong interaction. Additional file 1: Figure S1 displays the percentile of the interaction of graphical representation of these interactions in detail.

### Description of web based tools of LPIcom

LPIcom has three different modules namely ‘analysis of binding sites’, ‘comparison of multiple binding sites’ and ‘propensity based prediction’ implemented in the LPIcom website for the analysis and prediction of interacting amino acids for various ligands. We consider a case study of various ligands e.g. ATP, ADP, GTP, NAD and FAD etc. for illustrating these modules.

### Analysis of binding sites

**Fig. 4:**
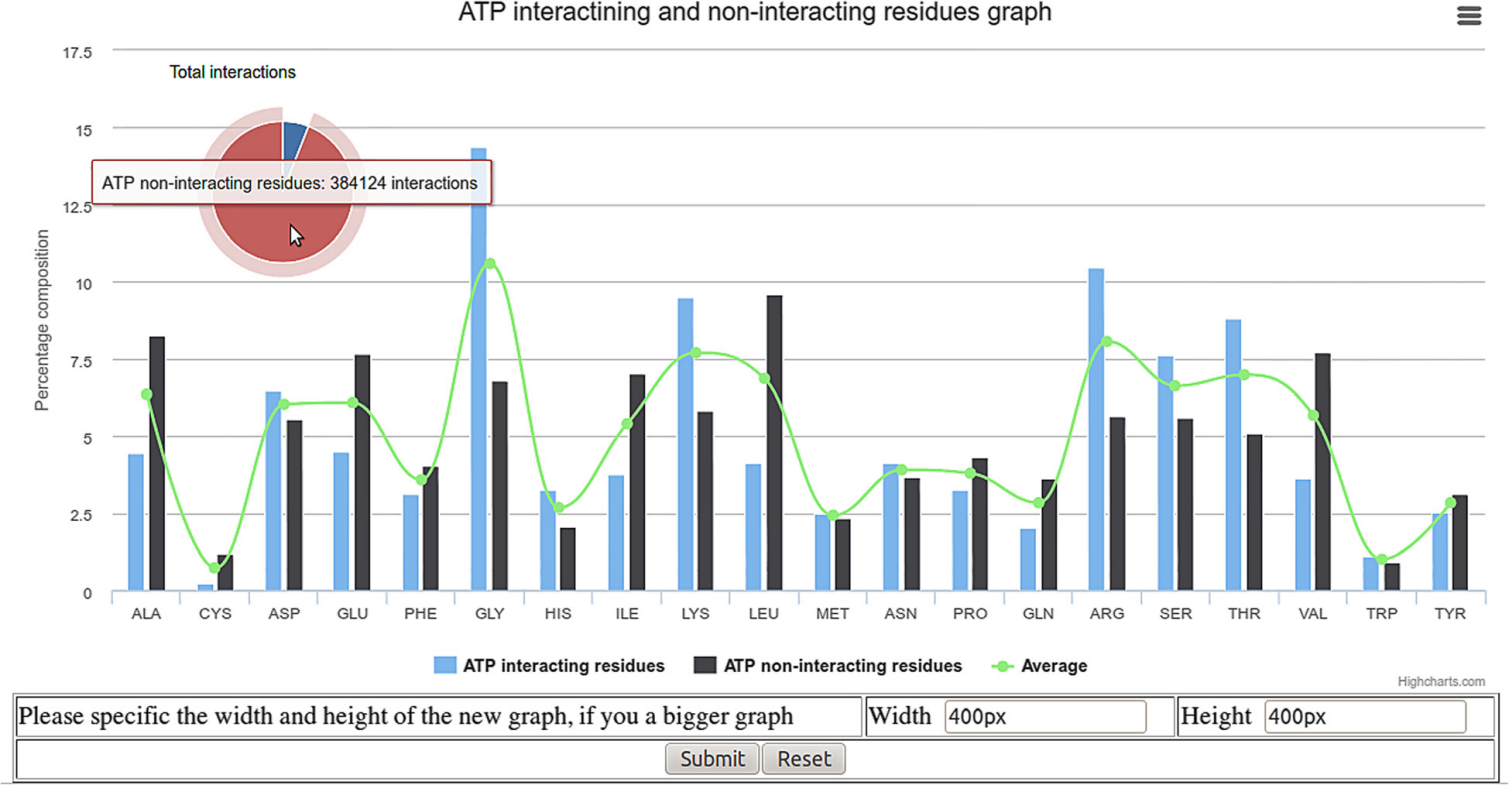
Example output of analysis module of server LPIcom for ATP with composition option; bar graph shows the composition of ATP interacting and non-interacting residues along with the average amino acid composition of proteins

This module calculates the amino acid composition of ligand interacting and non-interacting residues using equation 1. This server compute residue composition of ligand interacting and non-interacting residues. It shows the composition of interacting and non-interacting residues by a bar graph. In order to understand residue preference in ligand interaction, the server also shows the average amino acid composition of residues in proteins. This help user to understand whether the residue is preferred or not based on its composition, whether it is higher or lower than its average composition. In order to demonstrate the utility of this module of LPIcom server, we analyzed ATP binding sites (Fig. 4). It was observed that G, L and R amino acids are frequent binders followed by S and T amino acids. These results are in accordance with walker motif where G, L, S and T amino acids are dominated. Figure 4 also contains a pie chart in the left corner, which depicts the total number of ATP interacting and non-interacting residues.

**Fig. 5:**
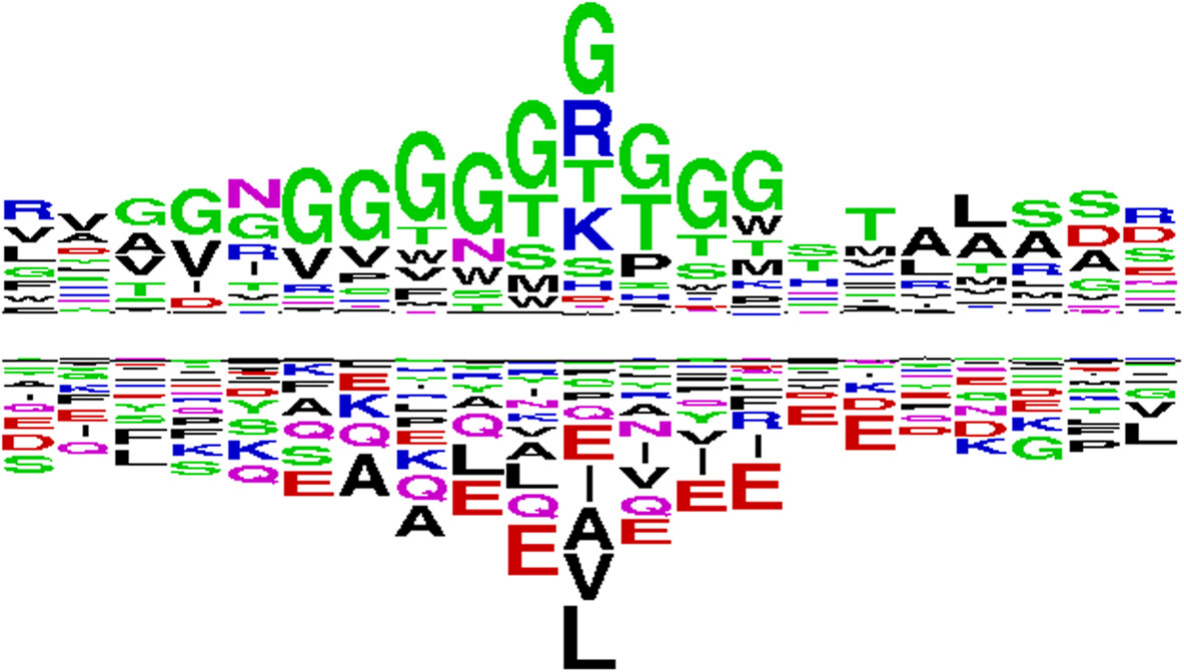
Two sample logo of ATP interacting and non-interacting residues using a window length of 21 amino acids. It is an example output of analysis module of LPIcom for ATP with logo option

**Fig. 6:**
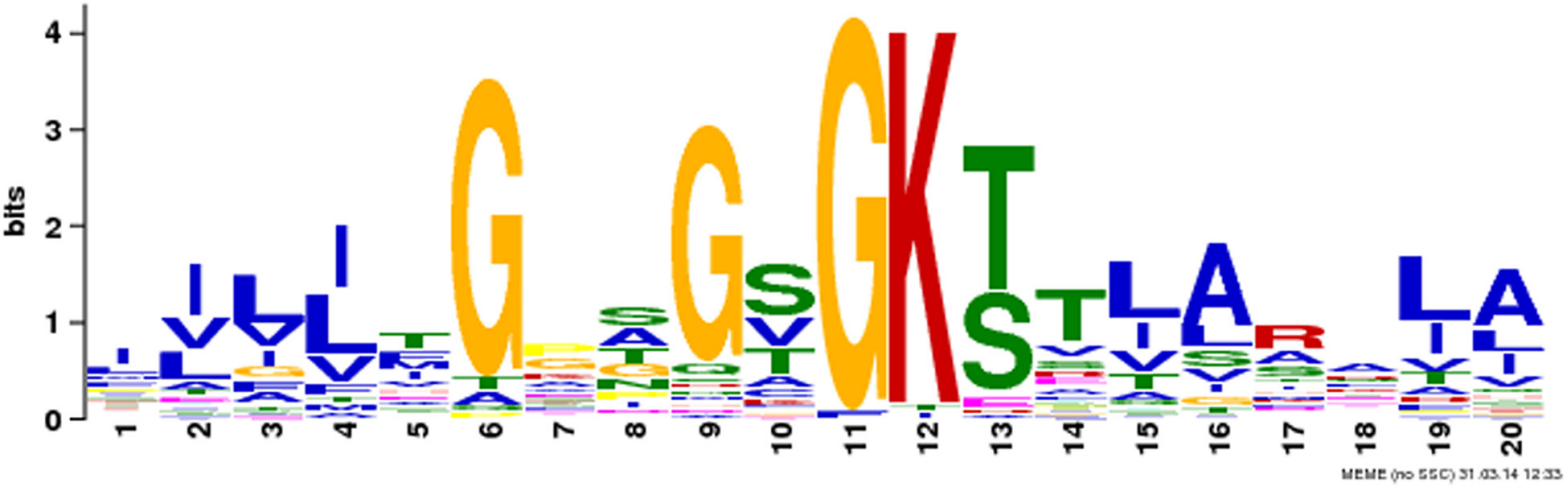
The motif detected in ATP interacting proteins, which resembles the walker motif

Analysis module of the server also allows the user to generate two sample logo for ligands based on their interaction. We created all possible overlapping pattern of length 21 residues in proteins; these patterns were classified as interacting and non-interacting patterns based on their central residue whether it is ligand interacting or non-interacting. The web logo of ATP interacting proteins (Additional file 1: Figure S2) shows the dominance of G, R, K, T and S amino acids. The most frequent neighbouring residues are G, T, and S amino acids. On the other hand, L and V amino acids occur as distant neighbouring residues. A further investigation seems necessary to understand the role of L-amino acid as a neighbour of ATP interacting residues. The two-sample logo is created for ATP using two-sample logo package to understand the preference of neighbour (Fig. 5). To detect potential motif in ATP interacting patterns, we used the meme program available in MEME suite [25]. Since motif detection is based on the frequency of the interacting pattern in a dataset, their equal representation is necessary for finding a motif. A 30 % non-redundant dataset of ATP interacting proteins is created, and meme program is used to find any potential motif. Motif depicted in Fig. 6 has a pattern of XXXXXGXXG[SVT]GK[TS] [TV][LIV][AL][RA]X[LI][AL]. The central part of the pattern (GXXG[SVT]GK[TS]) represents the walker motif (GXXXXGK(T/S). The motif is known in the case of ATP; however there are several ligand interacting motifs that need to be discovered. This tool helps in analyses of interacting and non-interacting residues based on the column chart and web logo, two sample logo and discovery of any potential motifs.

### Comparison of multiple ligands based on binding sites

As discussed in the above section, multiple ligands can be compared based on the interacting amino acids. The ligands can be compared based on either the amino acid composition of interacting amino acids or the propensity score of the interacting amino acids as described in methods section. We also implemented a third module for comparing ligands on the basis of the composition of physicochemical properties of interacting amino acids. Each module displays four charts namely column chart, area chart, pie chart and hierarchical cluster and the user can download the data as a text file. Next three sections briefly describe these three modules.

### Composition based on comparison of ligands

**Fig. 7:**
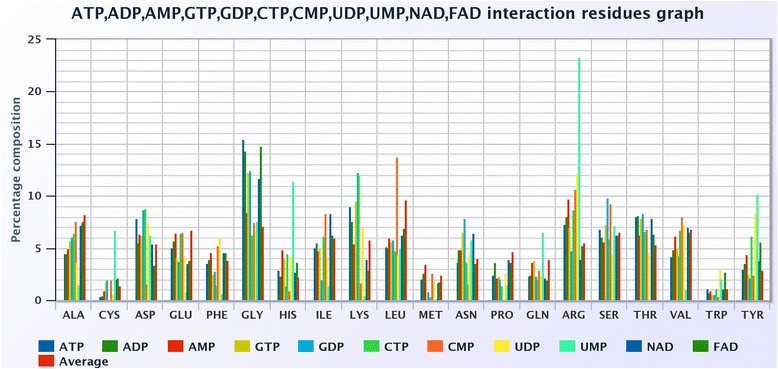
Amino acid composition of interacting residues of various ligands

We compared ten ligands based on the interacting amino acid composition computed using equation 1. The average amino acids composition of all the ligand is used for the reference dataset. Figure 7 depicts the percentage of composition of interacting amino acids with different ligands. It is clear that G amino acid has the highest frequency interaction with most of the ligands followed by R, S, T and V amino acids. On the other hand, C, M, P, Q and W amino acids show the least frequency. A, D, E, F, H, I, K, L, N and Y amino acids show moderate interaction with most of the ligands. UMP ligand with R amino acid shows unusually high frequency and, in general, the interaction of most of the amino acids is higher than average for this ligand as clear from Fig. 7.

### Propensity based on comparison of multiple ligands

**Fig. 8:**
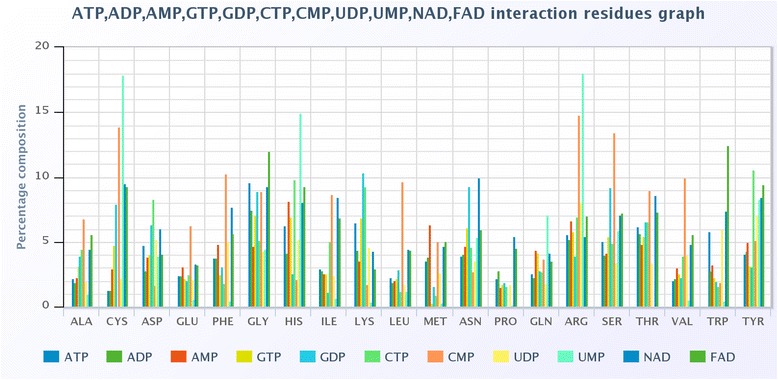
The propensity score of residues interacting with various nucleotides

We compared all these nucleotides based on their propensity score obtained from PDB using equation 3. In this case, the average propensity score of all amino acids cannot be calculated. Figure 8 shows the propensity score of different ligands as bar plot consists of all 20 amino acids. ATP has the least preference for C, A, V, Q, P, L, I and E amino acids while having a high preference or propensity score for G, D, W, T, S, K and H amino acids. Different ligands have different propensity score for the 20 amino acids. The analysis suggests that the propensity score is not dependent on the chemical property or size of the amino acid.

### Comparison ligands based on physicochemical composition of interacting amino acid

**Fig. 9:**
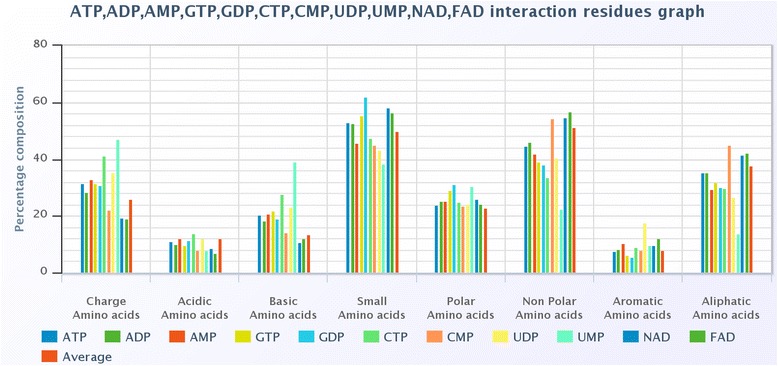
A column chart of various ligands based upon the physicochemical property of amino acids

First, amino acids were grouped into different categories on the basis of their physicochemical properties (e. g. charged, acidic, basic, small, polar, non-polar, aliphatic, and aromatic) than the composition of each class of amino acids is computed using equation 6. The average physicochemical property of all ligand interacting amino acids in PDB is also calculated as a reference. A column chart is created for ATP, ADP, GTP, NAD and FAD interacting amino acid. Charged amino acids, especially basic amino acids, are more favoured in ATP, ADP and GTP interaction compared to NAD and FAD interaction as clear from Fig. 9. Small and polar amino acids are equally represented in all five ligands. ATP, ADP and GTP ligands show lower than the average interaction for non-polar, aromatic and aliphatic amino acids. On the other hand, NAD and FAD ligands show a higher than average interaction. Thus, ligands can be divided into two groups 1) ATP, ADP and GTP and 2) NAD and FAD on the basis of their physicochemical properties.

### Propensity-based prediction

**Fig. 10:**
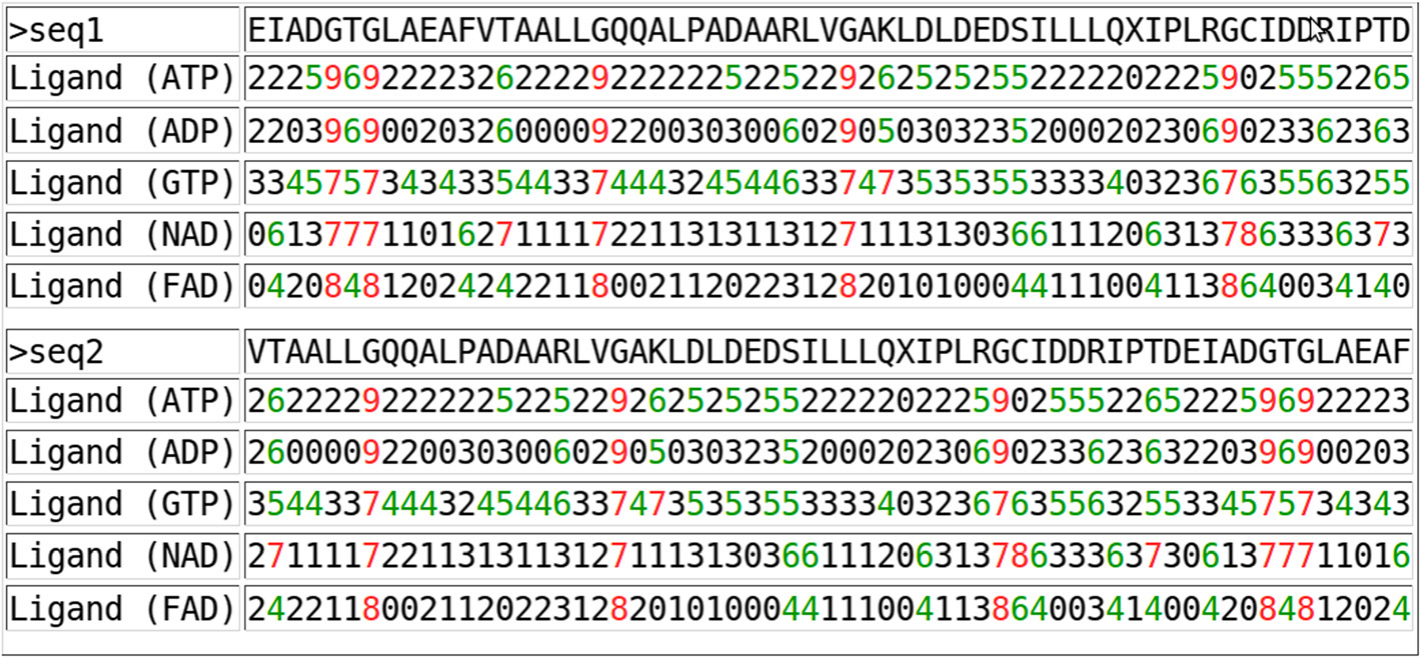
Propensity-based prediction of ATP, ADP, GTP, NAD and FAD interacting residues in test examples

The propensity based prediction method assign the propensity score of the residue according to the selected ligand using equation 3. These propensity scores are normalized in the range of 0–9 and depicted below the sequence (Fig. 10) using equation 4. The high probability region of ATP, ADP and GTP ligands are similar in the test sequence. Probability region of NAD and FAD ligands are also analogous to each other. We tested the performance of propensity based prediction on an independent dataset of 1301 PDB chains interacting with 50 different ligands. Each PDB chain is submitted to the prediction module with relevant ligand information and the predicted propensity scores are compared with the actual data to validate the performance. We achieved an average accuracy of 70 % (minimum 24.65 % to maximum 97.61 %, Additional file 1: Table S1). The region of amino acids having highest propensity score has a higher probability to interact with the given ligand. The similarity-based approach is recommended to use if machine learning methods are not available, and propensities based method is recommended for analysis/understanding of interacting residues in a given sequence.

## Conclusion

Binding preference analysis of a series of ligands with interacting amino acids is performed using a dataset of 824 ligands. Ligands are clustered based on the preference of interacting amino acids. The ligands having a similar preference for interacting residues have a higher probability of interaction with similar pockets if the difference in their size is not significantly bigger. Clustering the ligands based upon residue preference will help in better understanding of various ligand interactions. A web-based method named LPIcom is also developed for identification of favoured interacting residue with specific ligands. Three different approaches are used from LPIcom for analysis of interacting and non-interacting residues. 1) Comparison based on the amino acid composition of the specific ligand interacting and non-interacting residues. 2) Generation of ‘Two sample logo’ for comparison of interacting and non-interacting amino acids based upon *t*-test. 3) Detection of any potential motif in the interacting protein sequences using MEME suite. In addition, three modules are developed for comparison of interacting amino acids of multiple ligands. The first module compares the interacting amino acid composition of multiple ligands, the second module calculates the propensity score of interacting residue for each ligand and compares these propensity scores. The third module compares the physicochemical properties of amino acids for different ligands.

The propensity scores of various ligands are calculated, and the regions of all the protein sequences are highlighted on the basis of propensity scores. The propensity-based method can predict the probable interacting region for every ligand. These results may help biologist in better understanding the ligand interacting regions. Simply, the regions having highest propensity scores have the highest probability to interact with the ligand. The single ligand module helps to understand the interacting and non-interacting residues preference and to detect any potential motif in interacting PDB chains. It also provides a complete and non-redundant dataset for analysis and development of prediction methods. These comparison tools assist in analysing the amino acid preference of various ligands simultaneously. It is important for readers or users to understand limitation of web-server LPIcom describes in this study. As shown in Additional file 1: Table S3, median resolution of PDB chains for around 50 % ligands is poorer than 2.0 Å, even median resolution of 7 % ligands is poorer than 3.0 Å. It means prediction reliability of large number of ligands will be poor, as median of resolution of PDB chains is poor. In addition, user cannot use our server LPIcom for new ligands not included Additional file 1: Table S3.

## Method

The ligand interacting data was obtained from the ccPDB database [26] updated up to September 2015 release of PDB. The ligand-interacting amino acids information is extracted from PDB using the LPC software [27]. This software stores the information of each ligand interacting residue with the ligand name, number and chain id and the residue name, number and chain id. In this study, we consider ligand amino acid distance less than or equal to 4 Å for performing the analysis. In this study we only consider 824 ligands, having more than 30 binding sites in the PDB on the basis of the data release up to September 2015. The list of these 824 ligands is given in the Additional file 1: Table S2 and the respective PDBs resolution details are given in Additional file 1: Table S3.

### Ligand-specific amino acid composition

The percent amino acids composition of interacting residues (or residues in ligand binding sites) of each ligand is calculated using equation 1.

1 $$ \mathbf{R}{\mathbf{C}}_{\mathbf{i}}=\frac{{\mathbf{R}}_{\mathbf{i}}}{\mathbf{N}} \times 100 $$

Where ***RC***_***i***_ is the percent composition of a residue of type ***i***, ***R***_***i***_ is the number of residues of type ***i***, and **N** is the total the number of all twenty interacting residues.

### Similarity between two ligands based on their amino acid composition

In order to compute similarity or distance between two ligands, we compute similarity using amino acid composition of the ligand binding or interacting residues. The similarity between two ligands based on their amino acid composition is calculated using the Euclidean distance (ED). The composition based ED between two ligands **p** and **q** is calculated equation 2:

2 $$ \mathbf{C}\mathbf{E}{\mathbf{D}}_{\mathbf{p},\mathbf{q}}=\sqrt{{\displaystyle {\sum}_{\mathbf{i}=1}^{20}}\Big(\mathbf{R}{\mathbf{C}}_{\mathbf{i}}^{\mathbf{p}} - \mathbf{R}{\mathbf{C}}_{\mathbf{i}}^{\mathbf{q}}}\Big){}^2 $$

Where **CED**_**p,q**_ is distance between two ligands **p** and **q**, **RC**_**i**_^**p**^ is amino acid composition of residue type **i** for ligand **p** and, **RC**_**i**_^**p**^ is amino acid composition of residue type **i** for ligand **q**.

### Residues propensity for ligands

It is important to understand which residue is preferred or not preferred in binding sites of a ligand. In order to compute preference of a residue in binding site or interaction of a ligand, we compute residues propensity for each ligand. The ligand propensity for each type of residue for a given ligand is computed using equation 3.

3 $$ \mathbf{R}{\mathbf{P}}_{\mathbf{i}}=\frac{{\mathbf{R}}_{\mathbf{i}}}{{\mathbf{N}}_{\mathbf{i}}} \times 100 $$

Where **RP**_**i**_ is ligand propensity score for residue type **i**, **R**_**i**_ is number of interacting residues of type **i** and **N**_**i**_ is the total number of residues (interacting and non-interacting) of type **i**. The propensities were further normalized between 0 to 9 using equation 4.

4 $$ \mathbf{N}{\mathbf{P}}_{\mathbf{i}} = \frac{{\mathbf{P}}_{\mathbf{i}} - {\mathbf{P}}_{\mathbf{min}}}{{\mathbf{P}}_{\mathbf{max}} - {\mathbf{P}}_{\mathbf{min}}} \times 9 $$

Where **NP**_**i**_ is the normalized propensity of the residue type **i**, **P**_**min**_ is the minimum propensity score out of twenty amino acids and P_max_ is the maximum propensity score out of twenty amino acids.

### Similarity between two ligands based on their residues propensity

In order to compute the similarity between two ligands based on residues preferred in their interaction, we compute ED between their residues propensity. The propensity based ED between two ligands **p** and **q** is calculated using equation 5.

5 $$ \mathbf{P}\mathbf{E}{\mathbf{D}}_{\mathbf{p},\mathbf{q}}=\sqrt{{\displaystyle {\sum}_{\mathbf{i}=1}^{20}}\Big(\mathbf{R}{\mathbf{P}}_{\mathbf{i}}^{\mathbf{p}} - \mathbf{R}{\mathbf{P}}_{\mathbf{i}}^{\mathbf{q}}}\Big){}^2 $$

Where **PED**_**p,q**_ is distance between two ligands **p** and **q**, **RP**_**i**_^**p**^ is residue composition of residue type **i** for ligand **p** and **RP**_**i**_^**p**^ is residue composition of residue type **i** for ligand **q**.

### Physicochemical property based composition

In order to understand physicochemical property (e.g., charge, polar, hydrophobic) of residues involved a ligand binding residues. We group all twenty types of residues in eight class based on their physicochemical property. As shown in Table 1, we group amino acids based on their major characteristics that include their size, charge, polarity and hydrophobicity. We compute composition of each class of residues of these eight classes (or composition of physicochemical property) using following equation 6.

**Table 1:** The different physicochemical property of amino acids with the respective amino involved

Physicochemical property	Amino acid involved
Charge	ASP,GLU,LYS,HIS,ARG
Acidic amino acids	ASP,GLU
Basic amino acids	LYS,ARG,HIS
Small amino acids	PRO,ALA,CYS,GLY,SER,ASN,ASP,THR,VAL
Polar amino acids	SER,THR,TYR,ASN,GLN
Non polar amino acids	ALA,VAL,LEU,ILE,PRO,PHE,TRP,MET,CYS,GLY
Aromatic amino acids	PHE,TYR,TRP
Aliphatic amino acids	LEU,ILE,VAL,ALA,GLY

6 $$ \mathbf{P}{\mathbf{C}}_{\mathbf{i}}=\frac{{\mathbf{P}}_{\mathbf{i}}}{\mathbf{N}} \times 100 $$

Where **PC**_**i**_ is the percent composition of a physicochemical property of type **i**, **P**_**i**_ is the number of interacting residues having physicochemical property of type **i** and N is the total the number of interacting residues.

### Ligand similarity based on physicochemical property of residues

In order to compute the similarity between two ligands based on their physicochemical property of residues involved in their binding sites, we compute ED between compositions of their physicochemical properties. The composition based ED between two ligands **p** and **q** is calculated using equation 7.

7 $$ \mathbf{P}\mathbf{C}\mathbf{E}{\mathbf{D}}_{\mathbf{p},\mathbf{q}}=\sqrt{{\displaystyle {\sum}_{\mathbf{i}=1}^{20}}\Big(\mathbf{P}{\mathbf{C}}_{\mathbf{i}}^{\mathbf{p}} - \mathbf{P}{\mathbf{C}}_{\mathbf{i}}^{\mathbf{q}}}\Big){}^2 $$

Where **PCED**_**p,q**_ is distance between two ligands **p** and **q**, **PC**_**i**_^**p**^ is composition of physicochemical property of type **i** for ligand **p** and, **PC**_**i**_^**p**^ is residue composition of composition of physicochemical property **i** for ligand **q**.

### Clustering of Ligands

In this study, we have used the ‘dist function’ available in ‘R’ package to obtain the distance matrix between multiple ligands. The distance matrix is used for generating clusters based on hierarchical clustering algorithm embedded in ‘Hclust function’ available in ‘R’ package. The cluster information along with distance matrix is used to generate the heat map using ‘Heatmap function’ also available in ‘R’ package.

### Generation of the dynamic graph

High-charts library was used to display graph according to selected features. The generated charts can also be exported to various image formats. For creating web logo and two sample logo, we generated a pattern of window length 21 for a specific ligand interacting proteins with the central residue as the ligand-interacting residues. The web logo standalone package is used for displaying the logo of interacting amino acids [28]. A two sample logo is generated on the basis of interacting and non-interacting patterns using the default parameters [29]. Meme program from MEME suite [25] is used for motif identification in the non-redundant dataset of interacting proteins.

### Validation dataset

The LPIcom database was generated from PDB complexes released up to September 2015. In order to validate the performance of our prediction module, we created a validation dataset. A 1301 PDB chains, for 50 commonly found ligands, were selected from PDB complexes released between October-December 2015 (Additional file 1: Table S1). Thus, PDB chains in validation dataset are entirely different from PDB chains used for prediction in LPIcom.

## Reviewers’ comments

### Reviewer 1: Response to Prof Michael Gromiha

In this work, the authors developed a web server for predicting ligand binding sites in proteins. They have analyzed the binding propensity of more than 700 ligands and the topmost ones are presented in the manuscript. Further, the ligands/amino acid residues have been clustered to understand the preference of binding. The details about the binding sites and other details are provided in the Additional file 1 and on the web. It is an interesting manuscript with several ligands together.

The manuscript could be improved by incorporating the following suggestions.

1. Propensity analysis has been carried out based on high, moderate and low. The plausible reasons could be discussed.

Response: *We are thankful to the reviewer for the suggestion, in revised manuscript we clearly described propensity score in detail including modification of equations used for calculation. We defined preference of a residue in ligand binding site based on its propensity score if the score of a residue is lower than 5 then we called it low preference residue. Similarly, we called a residue moderate if it has propensity score between 5 to 12 and high if the score is more than 12. In revised manuscript, we incorporate suggestion of reviewer.*

2. Analysis on statistical significance would validate the specific preference of residues/ligands.

Response: *We agree with the reviewer that analysis should show whether the preference is really significant or it is by chance. In order to facilitate users to understand whether propensity or composition of ligand interacting residue is significant or not, we also compute and compare it with an average of each type of amino acids. This help user to understand whether a given residue is preferred in the binding site of a ligand. In revised version, we emphasize this point.*

3. Several examples are given on the binding site prediction of ligands using example proteins and ligands produced no binding site results. It is better to provide examples with binding site residues. Also, these results should be checked.

Response: *We are thankful to the reviewer for pointing the error. We have fixed all the errors.*

4. In the Additional file 1 prediction performance of specific ligands are given. It will be beneficial if the data for all ligands are given although some of them would be poor due to their less occurrence in proteins-ligand complexes.

Response: *We calculated the prediction performance of some of the ligands, which have significantly high frequency in the PDB. After getting comment of the reviewer, we also compute prediction accuracy for more ligands (50 ligands). It is not feasible to compute performance to all ligands (~800 ligands).*

5. Several methods are available for ligand binding site prediction. A comparison with other existing prediction methods could be useful.

Response: *Ideally one should compare newly developed prediction method with existing methods as suggested by a reviewer. In past our group also developed a number of methods for predicting ligand interacting residues (e.g., ATPint, NADbinder, GTPbinder, FADpred) where we compare their performance with existing methods. Development of prediction method even for a single ligand is time-consuming as one need to create clean datasets (e.g., non-redundant) and should evaluate using cross-validation techniques (internal and external validations). This is the reason, so far methods have been developed only for limited ligands. In this study, we described simple propensity based method for a large number of ligands. Though we also compute performance of our method on limited ligands but comparing performance with existing method will be unfair as we have not used clean dataset for training and cross-validation techniques. The objective of our method is to assist biologist in understanding the propensity scores of various amino acid and propensity based prediction of those ligands for which no specific method is available.*

### Reviewer 2: Response to Prof Vladimir Poroikov

In this paper freely available via Internet web-server LPIcom (Ligand-Protein Interactions Comparison and Analysis), which provides the possibility to study protein-ligand interactions, is described. The authors extracted from PDB the information about protein-ligand complexes for 724 ligands, which have 50 protein binding sites in PDB. This information was analyzed, to estimate the propensity of participating in protein-ligand interactions for each of twenty amino acid residues. Web-server consists of three modules provided Analysis, Comparison and Prediction functionalities. It provides the following facilities: a) assigning of ligand-interacting residues in a protein from the structure of protein-ligand complex; b) analysis of composition of ligand-specific interacting residues; c) comparison of binding sites of different ligands; d) generation of two sample logo of ligand binding sites; e) searching of ligand binding motifs; f) propensity-based prediction of ligand-interacting residues.

1 From the technical point of view, everything is well-done, except some misprints in the text at this web-site (e.g., “How to save and pritn the graph” - it should be “print”).

Response: *We are thankful to the reviewer for indicating the error and we have corrected the typing and grammatical mistakes in the revised manuscript.*

Also, according to my knowledge, this is the first analytics of massive data on protein-ligand interactions from PDB, where information about at least 50 binding sites is available for the ligands. However, some questions arose regarding the possibility of application of the obtained results in a prospective mode. The authors declare that “This information can be used to either enhance or diminish the binding strength of the given ligand” (page 3, lines 37–38 of the manuscript).

1. It is unclear if and how the developed web-server could be applied to the new ligands, which are not included into the “training set” (724 ligands).

Response: *The web-server cannot be applied to new ligands; we have increased the number of ligands from 724 to 824 which have a minimum number of ligand binding sites greater than 30. In future, we will update this database to include new ligands.*

2. It is necessary to explain how the user might use the information provided by this web-server, to "enhance or diminish the binding energy of the given ligand." Since such application is of great importance in the field of computer-aided drug design, it would be great if the authors could present at least one case study with such application in the manuscript. Such example(s) could be based on the retrospective data for already studied set of ligands belonging to the same chemical series.

Response: *In revised manuscript, we explain how this server can be used to enhance or to diminish a ligand binding site in a protein. This server provides propensity score or preference for each type of amino acid for a given ligand. Experimentalist may enhance ligand binding by mutating low propensity residue with high propensity residue in the binding site having similar physicochemical property. Similarly, one may also diminish ligand binding by mutating high propensity residue with low propensity residue. Every ligand has a specific preference towards a different type of residues, nucleotides-ligand prefer aliphatic residues and less preference for acidic residues. On the other hand, carbohydrates have more preference for acidic residues than aliphatic residues. Experimental researchers may use above information for increasing or decreasing binding affinity based propensity score. The server only suggests the residues based on the information available in the PDB. Multiple factors influence the binding strength of a residue in a given binding site apart from its affinity to interact with a particular ligand. The purpose of LPIcom is to provide the affinity information of residues toward different ligands as observed in PDB.*

Minor: It would be great if in the Additional file 1 the authors present the estimates of the quality of the X-ray data in the protein-ligand complexes analyzed for each studied ligand (median, minimum and maximum values characterized the resolution for all binding-sites under consideration).

Response: *The X-ray data of all PDB present in the LPIcom database are given in* Additional file 1*: Table S3. We have provided the median, minimum and maximum X-ray resolution for each ligand as shown in Additional file*1*: Table S3*.

Second Revision

Major comments: The authors have provided the responses on my major comments, and now the contents of their work is more clear for a scientific community. There is still some minor issues, which should be fixed prior to the publication. 1. It is necessary to provide the units for values presented in the Additional file 1: Table S3. 2. As one may see from the Additional file 1: Table S3, in some cases the median resolution in X-ray data is quite low (exceeded 2.00). The authors should comment in the manuscript if the obtained results are reliable enough in such cases. 3. It should be explicitely mentioned in the manuscript that the web-server cannot be applied for new ligands.

Response: *We are thankful to reviewer for appreciating our efforts. 1. In* Additional file 1: *Table S3 units of resolution (angstrom) has been stated. 2. Yes, median resolution of ~50 % ligands exceed 2.0 Å, even median resolution of ~7 % ligands exceed 3.0 Å. In revised manuscript, we clearly mentioned limitations of our study as number of ligands have PDB chains of poor resolution. In addition, we also mentioned in last paragraph of ‘Conclusion section’ that our web-server couldn’t by applied for new ligands.*

Minor: Despite the correction of grammatical errors and misprints, the authors added new errors/misprints in the novel part of the manuscript; e.g., Page 10, Line 57: “twnety” it should be “twenty”. The whole manuscript should be carefully checked, and all errors/misprints should be corrected. Despite the correction of grammatical errors and misprints, the authors added new errors/misprints in the novel part of the manuscript; e.g., Page 10, Line 57: “twnety” it should be “twenty”. The whole manuscript should be carefully checked, and all errors/misprints should be corrected.

Response: *We are grateful to the reviewer for indicating the grammatical errors. The manuscript has been carefully checked and corrected.*

### Reviewer 3: Response to Prof Zlatko Trajanoski

General comments The manuscript describes a web server for analysis of protein ligand binding sites. Although the topic is potentially of interest to a broader community, I don' see any considerable contribution neither from manuscript nor from the web server. The manuscript is difficult to read and the presented results seems to show simple statistical analysis of the amino acids which are binding ligands. What is the major contribution and how does this work add additional information compared to other papers?

Response: *Best of our knowledge this is a unique server which allows users to analyse, compare and predict potential binding sites for a large number of ligands based on information in PDB.*

Specifically, the work should be compared to the web servers already available (References 10 and 11) and the advantages/disadvantages highlighted.

Response: *Ideally one should compare newly developed prediction method with existing methods as suggested by a reviewer. In past our group also developed a number of methods for predicting ligand interacting residues (e.g., ATPint, NADbinder, GTPbinder, FADpred) where we compare their performance with existing methods. Development of prediction method even for a single ligand is a time consuming as one need to create clean datasets (e.g., non-redundant) and should evaluate cross-validation techniques (internal and external validations). This is the reason, so far methods have been developed only for limited ligands. In this study, we described simple propensity-based method for a large number of ligands. Though we also compute performance of our method on limited ligands but comparing performance with existing method will be unfair as we have not used clean dataset for training and cross-validation techniques. The objective of our method is to assist biologist in understanding the propensity scores of various amino acid and propensity based prediction of those ligands for which no specific method is available.*

Moreover, the web server itself was not thoroughly tested as evident by a number of issues raised bellow. Specific comments The implementation of the web server has several limitations some of which are provided below:

1) Typos and grammatical errors: For instance, from the input form and output of “analysis of binding sites” (http://crdd.osdd.net/raghava/lpicom/mut.php): - “User are required”; - “Click to Cutomize plot”; - “red color bars shows”; - “amino acid composition of all ligand”; - “High Resolutione”.

Response: *We check and removed all the errors from the revised manuscript and web-server.*

2) Inconsistencies in the descriptions: - The page of “ligand statistics” (http://crdd.osdd.net/raghava/lpicom/ligand-data.php) is once referred as “the complete list of ligands” (http://crdd.osdd.net/raghava/lpicom/mut.php) and once as the list of “highly frequent ligand” (http://crdd.osdd.net/raghava/lpicom/predict.php). The second option is probably the correct one, since the web server provides results also for ligands that are not present in the list. However, the full sentence is difficult to understand: “Detail of highly frequent ligand in PDB is available from and view ligands having highest occurence in PDB HERE”. - The description of results from “analysis of binding sites” (http://crdd.osdd.net/raghava/lpicom/mut.php), says: “blue color bars show ATP interacting and red color bars shows not interacting residues […]”. But there are no red bars, only blue or back ones.

Response: *We check and removed all the errors from the revised manuscript and web-server.*

3) Inconsistencies in the web pages and broken links If “Click to Cutomize plot” is selected on the “analysis of binding sites” results page (http://crdd.osdd.net/raghava/lpicom/mut.php), a different web page is shown. Some links, such as “interacting PDB” are broken.

Response: *We fixed these issues and now they are working fine.*

4) Not-working modules (?) - The example of the module “Comparison of Ligands Binding Sites (Amino acid Composition)” (http://crdd.osdd.net/raghava/lpicom/compare.php) gives 0.00 % result on all amino acids and ligands. - The “Prediction of Ligand Interacting Residues” module (http://crdd.osdd.net/raghava/lpicom/predict.php) predicts 0 propensity score for all positions (there are no regions highlighted in red or green.

Response: *We fixed these issues and now they are working fine.*

5) Finally, it would be useful to have descriptions of acronyms and link to external references (e.g. PDB), as well as a description of the full name of the ligand(s) for which the analysis was run, to have a confirmation of the selection.

Response: *The information is already provided on the web-server and in the Additional file*1. *We have updated the web server language for better understanding of the terminology.*

## Additional file

Additional file 1: Figure S1. The propensity score of residues interacting with various carbohydrates. **Figure S2.** A web logo of ATP interacting patterns of 21-window length. **Table S1.** Performance of our propensity based prediction models on 50 major ligands, evaluated on independent datasets. **Table S2.** List of 824 ligands having more than 30 binding sites in the PDB. **Table S3.** Minimum, Maximum and Median Resolution of PDBs interacting with 824 ligands. (DOCX 276 kb)

## Abbreviations

ATP: adenosine triphosphate
ADP: adenosine diphosphate
AMP: adenosine monophosphate
GTP: guanosine triphosphate
GDP: guanosine diphosphate
CTP: cytidine triphosphate
CMP: cytidine monophosphate
UDP: uridine diphosphate
UMP: uridine monophosphate
NAD: nicotinamide adenine dinucleotide
FAD: flavin adenine dinucleotide
GLA: Alpha D-galactose
GLC: Alpha-D-glucose
MAL: maltose
FRU: fructose
MAN: Alpha-D-mannose
TRE: trehalose
RIB: ribose
XLS: D-xylose (linear form)
Avr: average
