# Supplementary material for: A web server for analysis, comparison and prediction of protein ligand binding sites
Source: Biol Direct. 2016 Mar 25;11:14. doi: 10.1186/s13062-016-0118-5 (PMC4807588; doi:10.1186/s13062-016-0118-5)
Supplement: Additional file 1: Figure S1. — The propensity score of residues interacting with various carbohydrates. Figure S2. A web logo of ATP interacting patterns of 21-window length. Table S1. Performance of our propensity based prediction models on 50 major ligands, evaluated on independent datasets. Table S2. List of 824 ligands having more than 30 binding sites in the PDB. Table S3. Minimum, Maximum and Median Resolution of PDBs interacting with 824 ligands. (DOCX 276 kb) [file 13062_2016_118_MOESM1_ESM.docx]

**Supplementary Material**

**A web server for Analysis, Comparison and Prediction of Protein Ligand Binding Sites**

Harinder Singh, Hemant Kumar Srivastava and Gajendra P. S. Raghava*

Bioinformatics Centre, CSIR-Institute of Microbial Technology, Chandigarh 160036, India.

Email addresses:

HS - [harinder@imtech.res.in](mailto:harinder@imtech.res.in)

HKS - hemantkrsri@gmail.com

GPSR*- [raghava@imtech.res.in](mailto:raghava@imtech.res.in)

**Address for correspondence**

Email: [raghava@imtech.res.in](mailto:raghava@imtech.res.in)

Web: <http://www.imtech.res.in/raghava/>

Phone: +91-172-2690557

Fax: +91-172-2690632

*Corresponding author


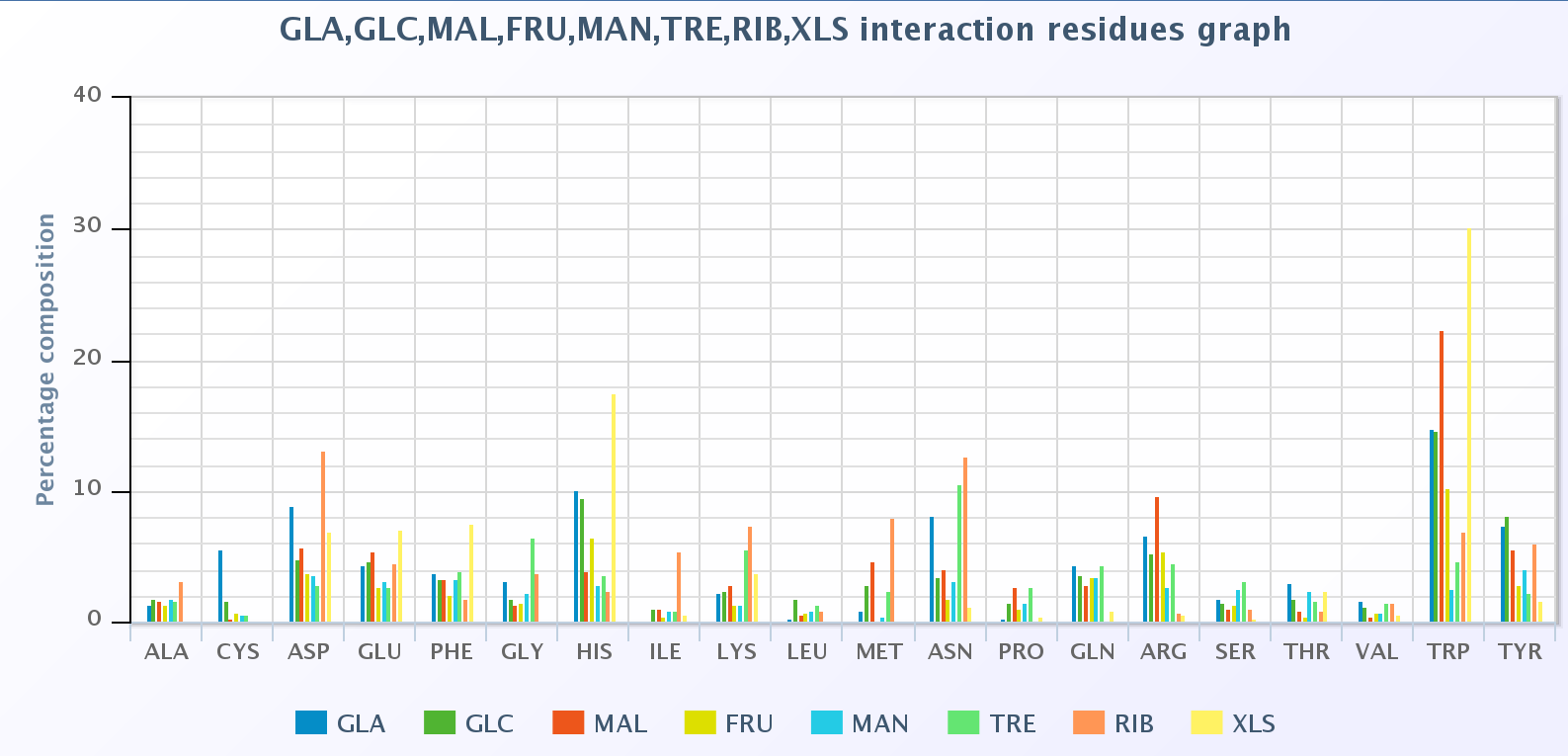


**Figure S1:** The propensity score of residues interacting with various carbohydrates.


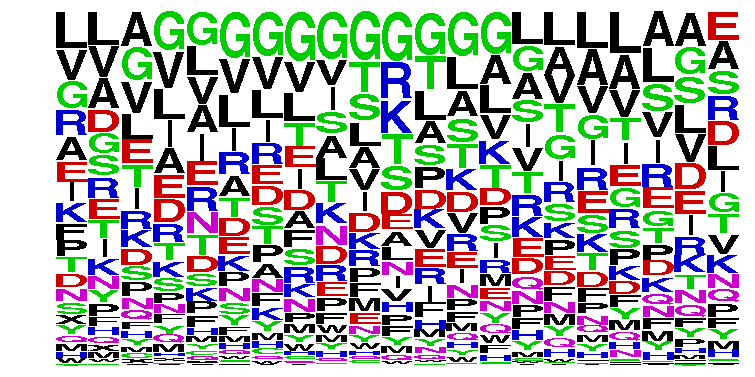


Figure S2: A web logo of ATP interacting patterns of 21-window length.

**Table S1:** Performance of our propensity based prediction models on 50 major ligands, evaluated on independent datasets.

| Ligand ID | No. of PDB Chains | Threshold | Sensitivity | Specificity | Accuracy | MCC | AUC |
| --- | --- | --- | --- | --- | --- | --- | --- |
| ACE | 13 | 3 | 47.5 | 69.22 | 68.94 | 0.04 | 0.59 |
| ACT | 31 | 4 | 44.12 | 79.6 | 78.94 | 0.08 | 0.65 |
| ACY | 9 | 2 | 84.31 | 24.67 | 25.63 | 0.03 | 0.54 |
| ADP | 26 | 5 | 69.84 | 55.19 | 55.62 | 0.08 | 0.65 |
| ATP | 19 | 4 | 50 | 61.63 | 61.16 | 0.05 | 0.58 |
| BGC | 13 | 2 | 53.12 | 71.92 | 71.44 | 0.09 | 0.69 |
| BMA | 36 | 4 | 27.37 | 90.24 | 89.79 | 0.05 | 0.64 |
| BME | 1 | 1 | 58.33 | 81.73 | 81.07 | 0.17 | 0.70 |
| BOG | 6 | 5 | 48.68 | 62.86 | 62.29 | 0.05 | 0.58 |
| CLA | 27 | 4 | 49.77 | 78.38 | 72.25 | 0.26 | 0.71 |
| COA | 14 | 5 | 50.37 | 58.76 | 58.42 | 0.04 | 0.59 |
| DMS | 24 | 5 | 19.39 | 83.1 | 80.37 | 0.01 | 0.51 |
| EDO | 95 | 2 | 55 | 57.47 | 57.38 | 0.05 | 0.58 |
| EPE | 7 | 2 | 33.78 | 78.52 | 77.64 | 0.04 | 0.57 |
| FAD | 22 | 4 | 57.83 | 60.46 | 60.28 | 0.1 | 0.61 |
| FE2 | 12 | 5 | 75 | 97.79 | 97.61 | 0.39 | 0.88 |
| FMN | 6 | 5 | 60.71 | 64.94 | 64.67 | 0.13 | 0.70 |
| FMT | 19 | 2 | 51.59 | 59.13 | 58.82 | 0.04 | 0.58 |
| FUC | 11 | 2 | 53.33 | 58.2 | 58.19 | 0.01 | 0.57 |
| GAL | 11 | 2 | 68.57 | 40.21 | 40.45 | 0.02 | 0.53 |
| GDP | 15 | 5 | 53.9 | 63.76 | 63.36 | 0.07 | 0.61 |
| GLC | 17 | 2 | 43.75 | 68.98 | 68.43 | 0.04 | 0.59 |
| GOL | 267 | 5 | 22.69 | 87.4 | 85.64 | 0.05 | 0.60 |
| HEC | 12 | 5 | 26.56 | 97.25 | 93.38 | 0.27 | 0.72 |
| HEM | 49 | 4 | 51.08 | 76.79 | 74.55 | 0.18 | 0.70 |
| HYP | 7 | 2 | 73.97 | 75 | 74.85 | 0.37 | 0.76 |
| IOD | 8 | 5 | 45.65 | 76.18 | 74.83 | 0.1 | 0.66 |
| IPA | 5 | 5 | 6.06 | 90.34 | 88.14 | -0.02 | 0.43 |
| MAN | 44 | 5 | 79.28 | 36.52 | 37.29 | 0.04 | 0.57 |
| MES | 20 | 5 | 20 | 90.24 | 89.22 | 0.04 | 0.67 |
| MG | 225 | 5 | 40.93 | 94.51 | 94.17 | 0.12 | 0.82 |
| MLY | 3 | 4 | 86.99 | 17.59 | 24.65 | 0.04 | 0.53 |
| MPD | 30 | 5 | 27.8 | 83.21 | 81.72 | 0.05 | 0.55 |
| MSE | 66 | 5 | 65.49 | 47.95 | 50.17 | 0.09 | 0.59 |
| NAD | 15 | 5 | 55.59 | 58.3 | 58.11 | 0.07 | 0.60 |
| NAG | 131 | 5 | 28.14 | 95.18 | 93.55 | 0.16 | 0.67 |
| NAP | 34 | 5 | 55.65 | 68.57 | 67.46 | 0.14 | 0.65 |
| NDG | 1 | 5 | 12.5 | 95.34 | 93.9 | 0.05 | 0.52 |
| NDP | 31 | 5 | 58.68 | 64.94 | 64.46 | 0.13 | 0.60 |
| NO3 | 9 | 4 | 53.49 | 75.75 | 75.33 | 0.09 | 0.67 |
| PEG | 37 | 4 | 56.28 | 62.17 | 62.05 | 0.05 | 0.62 |
| PG4 | 12 | 4 | 24.53 | 77.8 | 75.99 | 0.01 | 0.54 |
| PLP | 16 | 4 | 62.11 | 69.94 | 69.74 | 0.11 | 0.70 |
| PO4 | 74 | 4 | 40.08 | 87.75 | 86.78 | 0.12 | 0.68 |
| SEP | 18 | 5 | 67.86 | 66.29 | 66.33 | 0.11 | 0.73 |
| SF4 | 25 | 5 | 34.42 | 98.75 | 95.4 | 0.43 | 0.70 |
| SO4 | 297 | 4 | 35.51 | 87.52 | 86.2 | 0.11 | 0.67 |
| TPO | 9 | 5 | 50 | 85.09 | 84.35 | 0.14 | 0.71 |
| TRS | 16 | 4 | 69.44 | 66.9 | 66.95 | 0.11 | 0.72 |
| UNX | 18 | 4 | 73.78 | 41.94 | 43.34 | 0.07 | 0.59 |

**Table S2:** List of 823 ligands having more than 30 binding sites in the PDB.

| Ligand ID | No. of  PDB Chains | No. of binding sites | Ligand Formula | Ligand MW | Ligand Name |
| --- | --- | --- | --- | --- | --- |
| MSE | 14468 | 95031 | C5 H11 N O2 SE | 196.11 | SELENOMETHIONINE |
| MG | 18811 | 51324 | Mg 2 | 24.31 | MAGNESIUM ION |
| SO4 | 20045 | 48601 | O4 S -2 | 96.06 | SULFATE ION |
| GOL | 13272 | 31349 | C3 H8 O3 | 92.09 | GLYCEROL |
| ZN | 14406 | 22679 | ZN 2 | 65.38 | ZINC ION |
| EDO | 5621 | 21653 | C2 H6 O2 | 62.07 | 1,2-ETHANEDIOL |
| CA | 11761 | 21241 | CA 2 | 40.08 | CALCIUM ION |
| NAG | 6482 | 20968 | C8 H15 N O6 | 221.21 | N-ACETYL-D-GLUCOSAMINE |
| CL | 10980 | 19182 | CL -1 | 35.45 | CHLORIDE ION |
| NA | 6519 | 11170 | NA 1 | 22.99 | SODIUM ION |
| PO4 | 6117 | 10896 | O4 P -3 | 94.97 | PHOSPHATE ION |
| HEM | 6428 | 8469 | C34 H32 FE N4 O4 | 616.5 | PROTOPORPHYRIN IX CONTAINING FE |
| ACT | 3755 | 6987 | C2 H3 O2 -1 | 59.04 | ACETATE ION |
| DMS | 948 | 6347 | C2 H6 O S | 78.13 | DIMETHYL SULFOXIDE |
| MN | 3898 | 6325 | MN 2 | 54.94 | MANGANESE (II) ION |
| K | 2762 | 4269 | K 1 | 39.1 | POTASSIUM ION |
| IOD | 664 | 4078 | I -1 | 126.9 | IODIDE ION |
| FAD | 2947 | 4053 | C27 H33 N9 O15 P2 | 785.56 | FLAVIN-ADENINE DINUCLEOTIDE |
| CD | 1356 | 3745 | CD 2 | 112.41 | CADMIUM ION |
| UNX | 604 | 3630 | X | 0 | UNKNOWN ATOM OR ION |
| MAN | 1421 | 3622 | C6 H12 O6 | 180.16 | ALPHA-D-MANNOSE |
| FE | 2400 | 3534 | FE 3 | 55.85 | FE (III) ION |
| ADP | 2704 | 3407 | C10 H15 N5 O10 P2 | 427.2 | ADENOSINE-5'-DIPHOSPHATE |
| CLA | 577 | 3218 | C55 H72 MG N4 O5 2 | 893.5 | CHLOROPHYLL A |
| NAD | 2448 | 3143 | C21 H27 N7 O14 P2 | 663.43 | NICOTINAMIDE-ADENINE-DINUCLEOTIDE |
| PEG | 1674 | 3140 | C4 H10 O3 | 106.12 | DI(HYDROXYETHYL)ETHER |
| HYP | 332 | 3108 | C5 H9 N O3 | 131.13 | 4-HYDROXYPROLINE |
| MPD | 1456 | 2962 | C6 H14 O2 | 118.18 | (4S)-2-METHYL-2,4-PENTANEDIOL |
| CU | 1597 | 2789 | CU 2 | 63.55 | COPPER (II) ION |
| FMT | 889 | 2641 | C H2 O2 | 46.03 | FORMIC ACID |
| MLY | 349 | 2602 | C8 H18 N2 O2 | 174.24 | N-DIMETHYL-LYSINE |
| ACE | 2175 | 2534 | C2 H4 O | 44.05 | ACETYL GROUP |
| FE2 | 1084 | 2326 | FE 2 | 55.85 | FE (II) ION |
| PLP | 1413 | 2118 | C8 H10 N O6 P | 247.14 | PYRIDOXAL-5'-PHOSPHATE |
| ATP | 1509 | 2062 | C10 H16 N5 O13 P3 | 507.18 | ADENOSINE-5'-TRIPHOSPHATE |
| GLC | 667 | 2028 | C6 H12 O6 | 180.16 | ALPHA-D-GLUCOSE |
| ACY | 1030 | 1974 | C2 H4 O2 | 60.05 | ACETIC ACID |
| BR | 405 | 1964 | BR -1 | 79.9 | BROMIDE ION |
| NAP | 1726 | 1953 | C21 H28 N7 O17 P3 | 743.41 | NADP NICOTINAMIDE-ADENINE-DINUCLEOTIDE PHOSPHATE |
| BME | 1060 | 1786 | C2 H6 O S | 78.13 | BETA-MERCAPTOETHANOL |
| FMN | 1288 | 1768 | C17 H21 N4 O9 P | 456.35 | FLAVIN MONONUCLEOTIDE |
| NH2 | 1563 | 1678 | H2 N | 16.02 | AMINO GROUP |
| NI | 1099 | 1674 | NI 2 | 58.7 | NICKEL (II) ION |
| SR | 680 | 1625 | SR 2 | 87.62 | STRONTIUM ION |
| PG4 | 907 | 1621 | C8 H18 O5 | 194.23 | TETRAETHYLENE GLYCOL |
| SF4 | 1028 | 1589 | FE4 S4 | 351.63 | IRON/SULFUR CLUSTER |
| GAL | 1166 | 1588 | C6 H12 O6 | 180.16 | BETA-D-GALACTOSE |
| BCR | 647 | 1521 | C40 H56 | 536.88 | BETA-CAROTENE |
| MES | 1114 | 1500 | C6 H13 N O4 S | 195.23 | 2-(N-MORPHOLINO)-ETHANESULFONIC ACID |
| TRS | 1042 | 1486 | C4 H12 N O3 1 | 122.14 | 2-AMINO-2-HYDROXYMETHYL-PROPANE-1,3-DIOL |
| HG | 619 | 1455 | HG 2 | 200.59 | MERCURY (II) ION |
| HEC | 508 | 1451 | C34 H34 FE N4 O4 | 618.51 | HEME C |
| BGC | 760 | 1437 | C6 H12 O6 | 180.16 | BETA-D-GLUCOSE |
| BMA | 975 | 1429 | C6 H12 O6 | 180.16 | BETA-D-MANNOSE |
| SEP | 997 | 1324 | C3 H8 N O6 P | 185.07 | PHOSPHOSERINE |
| LDA | 372 | 1322 | C14 H31 N O | 229.4 | LAURYL DIMETHYLAMINE-N-OXIDE |
| GDP | 1118 | 1281 | C10 H15 N5 O11 P2 | 443.2 | GUANOSINE-5'-DIPHOSPHATE |
| CO | 703 | 1261 | CO 2 | 58.93 | COBALT (II) ION |
| BCL | 316 | 1248 | C55 H74 MG N4 O6 | 911.52 | BACTERIOCHLOROPHYLL A |
| NO3 | 488 | 1229 | N O3 -1 | 62.01 | NITRATE ION |
| COA | 766 | 1200 | C21 H36 N7 O16 P3 S | 767.53 | COENZYME A |
| TPO | 1047 | 1190 | C4 H10 N O6 P | 199.1 | PHOSPHOTHREONINE |
| FUC | 858 | 1162 | C6 H12 O5 | 164.16 | ALPHA-L-FUCOSE |
| BOG | 499 | 1154 | C14 H28 O6 | 292.37 | B-OCTYLGLUCOSIDE |
| EPE | 869 | 1138 | C8 H18 N2 O4 S | 238.3 | 4-(2-HYDROXYETHYL)-1-PIPERAZINE ETHANESULFONIC ACID |
| NDP | 949 | 1109 | C21 H30 N7 O17 P3 | 745.43 | NADPH DIHYDRO-NICOTINAMIDE-ADENINE-DINUCLEOTIDE PHOSPHATE |
| IPA | 556 | 1097 | C3 H8 O | 60.1 | ISOPROPYL ALCOHOL |
| PTR | 802 | 1071 | C9 H12 N O6 P | 261.17 | O-PHOSPHOTYROSINE |
| CIT | 770 | 1065 | C6 H8 O7 | 192.12 | CITRIC ACID |
| NDG | 687 | 1060 | C8 H15 N O6 | 221.21 | 2-(ACETYLAMINO)-2-DEOXY-A-D-GLUCOPYRANOSE |
| LMT | 455 | 1055 | C24 H46 O11 | 510.62 | DODECYL-BETA-D-MALTOSIDE |
| FES | 896 | 1054 | FE2 S2 | 175.81 | FE2/S2 (INORGANIC) CLUSTER |
| ANP | 813 | 1017 | C10 H17 N6 O12 P3 | 506.2 | PHOSPHOAMINOPHOSPHONIC ACID-ADENYLATE ESTER |
| GTP | 778 | 999 | C10 H16 N5 O14 P3 | 523.18 | GUANOSINE-5'-TRIPHOSPHATE |
| SAH | 851 | 924 | C14 H20 N6 O5 S | 384.41 | S-ADENOSYL-L-HOMOCYSTEINE |
| AMP | 685 | 922 | C10 H14 N5 O7 P | 347.22 | ADENOSINE MONOPHOSPHATE |
| LMG | 395 | 918 | C45 H86 O10 | 787.17 | 1,2-DISTEAROYL-MONOGALACTOSYL-DIGLYCERIDE |
| IMD | 552 | 904 | C3 H5 N2 1 | 69.09 | IMIDAZOLE |
| PGE | 584 | 884 | C6 H14 O4 | 150.17 | TRIETHYLENE GLYCOL |
| 1PE | 455 | 865 | C10 H22 O6 | 238.28 | PENTAETHYLENE GLYCOL |
| MLE | 230 | 840 | C7 H15 N O2 | 145.2 | N-METHYLLEUCINE |
| LLP | 535 | 820 | C14 H24 N3 O7 P | 377.33 | 2-LYSINE(3-HYDROXY-2-METHYL-5-PHOSPHONOOXYMETHYL-PYRIDIN-4-YLMETHANE) |
| CSO | 600 | 810 | C3 H7 N O3 S | 137.15 | S-HYDROXYCYSTEINE |
| MRD | 397 | 785 | C6 H14 O2 | 118.18 | (4R)-2-METHYLPENTANE-2,4-DIOL |
| DMF | 139 | 775 | C3 H7 N O | 73.09 | DIMETHYLFORMAMIDE |
| DGD | 381 | 754 | C51 H96 O15 | 949.31 | DIGALACTOSYL DIACYL GLYCEROL (DGDG) |
| KCX | 674 | 727 | C7 H14 N2 O4 | 190.2 | LYSINE NZ-CARBOXYLIC ACID |
| CME | 388 | 715 | C5 H11 N O3 S2 | 197.27 | S,S-(2-HYDROXYETHYL)THIOCYSTEINE |
| TRP | 369 | 680 | C11 H12 N2 O2 | 204.23 | TRYPTOPHAN |
| PCA | 582 | 673 | C5 H7 N O3 | 129.12 | PYROGLUTAMIC ACID |
| EOH | 217 | 624 | C2 H6 O | 46.07 | ETHANOL |
| CYC | 259 | 624 | C33 H40 N4 O6 | 588.7 | PHYCOCYANOBILIN |
| SQD | 421 | 609 | C41 H78 O12 S | 795.12 | 1,2-DI-O-ACYL-3-O-[6-DEOXY-6-SULFO-ALPHA-D-GLUCOPYRANOSYL]-SN-GLYCEROL |
| FLC | 444 | 605 | C6 H5 O7 -3 | 189.1 | CITRATE ANION |
| CDL | 423 | 589 | C81 H156 O17 P2 -2 | 1464.06 | CARDIOLIPIN |
| SCN | 317 | 586 | C N S -1 | 58.08 | THIOCYANATE ION |
| XYP | 207 | 578 | C5 H10 O5 | 150.13 | BETA-D-XYLOPYRANOSE |
| LHG | 252 | 576 | C38 H75 O10 P | 722.98 | 1,2-DIPALMITOYL-PHOSPHATIDYL-GLYCEROLE |
| SIA | 398 | 574 | C11 H19 N O9 | 309.27 | O-SIALIC ACID |
| SMC | 273 | 569 | C4 H9 N O2 S | 135.18 | S-METHYLCYSTEINE |
| DAL | 412 | 554 | C3 H7 N O2 | 89.09 | D-ALANINE |
| NCO | 239 | 551 | CO H18 N6 3 | 161.12 | COBALT HEXAMMINE(III) |
| PEE | 309 | 547 | C41 H83 N O8 P 1 | 749.08 | PHOSPHATIDYLETHANOLAMINE |
| CSD | 423 | 545 | C3 H7 N O4 S | 153.15 | CYSTEINE-S-DIOXIDE |
| CGU | 85 | 534 | C6 H9 N O6 | 191.14 | GAMMA-CARBOXY-GLUTAMIC ACID |
| PYR | 420 | 523 | C3 H4 O3 | 88.06 | PYRUVIC ACID |
| UMP | 294 | 519 | C9 H13 N2 O8 P | 308.18 | 2'-DEOXYURIDINE 5'-MONOPHOSPHATE |
| GAI | 193 | 519 | C H5 N3 | 59.07 | GUANIDINE |
| SAM | 439 | 514 | C15 H22 N6 O5 S | 398.44 | S-ADENOSYLMETHIONINE |
| UDP | 490 | 513 | C9 H14 N2 O12 P2 | 404.16 | URIDINE-5'-DIPHOSPHATE |
| PX4 | 61 | 511 | C36 H73 N O8 P 1 | 678.95 | 1,2-DIMYRISTOYL-SN-GLYCERO-3-PHOSPHOCHOLINE |
| GLA | 296 | 507 | C6 H12 O6 | 180.16 | ALPHA D-GALACTOSE |
| CO3 | 404 | 507 | C O3 -2 | 60.01 | CARBONATE ION |
| C8E | 125 | 504 | C16 H34 O5 | 306.44 | (HYDROXYETHYLOXY)TRI(ETHYLOXY)OCTANE |
| OLC | 76 | 491 | C21 H40 O4 | 356.55 | (2R)-2,3-DIHYDROXYPROPYL (9Z)-OCTADEC-9-ENOATE |
| CAC | 335 | 472 | C2 H6 As O2 -1 | 136.99 | CACODYLATE ION |
| DIO | 217 | 471 | C4 H8 O2 | 88.11 | 1,4-DIETHYLENE DIOXIDE |
| CMO | 451 | 468 | C O | 28.01 | CARBON MONOXIDE |
| URE | 113 | 467 | C H4 N2 O | 60.06 | UREA |
| FME | 365 | 464 | C6 H11 N O3 S | 177.22 | N-FORMYLMETHIONINE |
| DLE | 106 | 461 | C6 H13 N O2 | 131.17 | D-LEUCINE |
| GLU | 409 | 458 | C5 H9 N O4 | 147.13 | GLUTAMIC ACID |
| TLA | 347 | 446 | C4 H6 O6 | 150.09 | L(+)-TARTARIC ACID |
| XE | 152 | 445 | XE | 131.3 | XENON |
| TPP | 265 | 439 | C12 H19 N4 O7 P2 S 1 | 425.31 | THIAMINE DIPHOSPHATE |
| IPH | 251 | 439 | C6 H6 O | 94.11 | PHENOL |
| OXY | 415 | 438 | O2 | 32 | OXYGEN MOLECULE |
| ABA | 227 | 433 | C4 H9 N O2 | 103.12 | ALPHA-AMINOBUTYRIC ACID |
| ARG | 243 | 432 | C6 H15 N4 O2 1 | 175.21 | ARGININE |
| POP | 357 | 428 | H2 O7 P2 -2 | 175.96 | PYROPHOSPHATE 2- |
| GNP | 382 | 427 | C10 H17 N6 O13 P3 | 522.2 | PHOSPHOAMINOPHOSPHONIC ACID-GUANYLATE ESTER |
| PSU | 257 | 421 | C9 H13 N2 O9 P | 324.18 | PSEUDOURIDINE-5'-MONOPHOSPHATE |
| GSH | 255 | 420 | C10 H17 N3 O6 S | 307.32 | GLUTATHIONE |
| PLM | 256 | 418 | C16 H32 O2 | 256.43 | PALMITIC ACID |
| AZI | 287 | 411 | N3 -1 | 42.02 | AZIDE ION |
| BA | 125 | 408 | BA 2 | 137.33 | BARIUM ION |
| BEN | 272 | 398 | C7 H8 N2 | 120.15 | BENZAMIDINE |
| TYS | 355 | 396 | C9 H11 N O6 S | 261.25 | O-SULFO-L-TYROSINE |
| CHD | 187 | 396 | C24 H40 O5 | 408.58 | CHOLIC ACID |
| BPH | 201 | 393 | C55 H76 N4 O6 | 889.23 | BACTERIOPHEOPHYTIN A |
| MYR | 211 | 387 | C14 H28 O2 | 228.37 | MYRISTIC ACID |
| CU1 | 264 | 385 | CU 1 | 63.55 | COPPER (I) ION |
| GLY | 202 | 379 | C2 H5 N O2 | 75.07 | GLYCINE |
| SUC | 280 | 378 | C12 H22 O11 | 342.3 | SUCROSE |
| M3L | 347 | 372 | C9 H21 N2 O2 1 | 189.28 | N-TRIMETHYLLYSINE |
| CS | 125 | 370 | CS 1 | 132.9 | CESIUM ION |
| U10 | 233 | 358 | C59 H90 O4 | 863.36 | UBIQUINONE-10 |
| H4B | 217 | 356 | C9 H15 N5 O3 | 241.25 | 5,6,7,8-TETRAHYDROBIOPTERIN |
| CAP | 201 | 353 | C6 H14 O13 P2 | 356.12 | 2-CARBOXYARABINITOL-1,5-DIPHOSPHATE |
| SIN | 207 | 350 | C4 H6 O4 | 118.09 | SUCCINIC ACID |
| DTT | 239 | 345 | C4 H10 O2 S2 | 154.24 | 2,3-DIHYDROXY-1,4-DITHIOBUTANE |
| ACO | 235 | 338 | C23 H38 N7 O17 P3 S | 809.57 | ACETYL COENZYME *A |
| PGO | 119 | 332 | C3 H8 O2 | 76.1 | S-1,2-PROPANEDIOL |
| MLI | 210 | 323 | C3 H2 O4 -2 | 102.05 | MALONATE ION |
| P6G | 240 | 322 | C12 H26 O7 | 282.33 | HEXAETHYLENE GLYCOL |
| LFA | 34 | 321 | C20 H42 | 282.55 | EICOSANE |
| PL9 | 220 | 312 | C53 H80 O2 | 749.21 | 2,3-DIMETHYL-5-(3,7,11,15,19,23,27,31,35-NONAMETHYL-2,6,10,14,18,22,26,30,34-HEXATRIACONTANONAENYL-2,5-CYCLOHEXADIENE-1,4-DIONE-2,3-DIMETHYL-5-SOLANESYL-1,4-BENZOQUINONE |
| IUM | 65 | 310 | O2 U 2 | 270.03 | URANYL (VI) ION |
| DMU | 181 | 305 | C22 H42 O11 | 482.57 | DECYL-BETA-D-MALTOPYRANOSIDE |
| YCM | 168 | 304 | C5 H10 N2 O3 S | 178.21 | S-(2-AMINO-2-OXOETHYL)-L-CYSTEINE |
| RCO | 149 | 303 | C6 H6 O2 | 110.11 | RESORCINOL |
| AKG | 244 | 300 | C5 H6 O5 | 146.1 | 2-OXOGLUTARIC ACID |
| MME | 164 | 296 | C6 H13 N O2 S | 163.23 | N-METHYL METHIONINE |
| CMP | 155 | 293 | C10 H12 N5 O6 P | 329.21 | ADENOSINE-3',5'-CYCLIC-MONOPHOSPHATE |
| TL | 80 | 292 | TL 1 | 204.37 | THALLIUM (I) ION |
| PGV | 162 | 281 | C40 H77 O10 P | 749.02 | (1R)-2-{[{[(2S)-2,3-DIHYDROXYPROPYL]OXY}(HYDROXY)PHOSPHORYL]OXY}-1-[(PALMITOYLOXY)METHYL]ETHYL (11E)-OCTADEC-11-ENOATE |
| SAR | 222 | 278 | C3 H7 N O2 | 89.09 | SARCOSINE |
| NAI | 224 | 278 | C21 H29 N7 O14 P2 | 665.45 | 1,4-DIHYDRONICOTINAMIDE ADENINE DINUCLEOTIDE |
| LI1 | 31 | 277 | C42 H86 O3 | 639.14 | 1-[2,6,10.14-TETRAMETHYL-HEXADECAN-16-YL]-2-[2,10,14-TRIMETHYLHEXADECAN-16-YL]GLYCEROL |
| BCT | 237 | 274 | C H O3 -1 | 61.02 | BICARBONATE ION |
| ALY | 216 | 269 | C8 H16 N2 O3 | 188.23 | N(6)-ACETYLLYSINE |
| CYN | 240 | 263 | C N -1 | 26.02 | CYANIDE ION |
| CSX | 176 | 260 | C3 H7 N O3 S | 137.15 | S-OXY CYSTEINE |
| OXL | 182 | 255 | C2 O4 -2 | 88.02 | OXALATE ION |
| NH4 | 177 | 255 | H4 N 1 | 18.04 | AMMONIUM ION |
| RET | 250 | 252 | C20 H28 O | 284.44 | RETINAL |
| BNG | 62 | 252 | C15 H30 O6 | 306.4 | B-NONYLGLUCOSIDE |
| F3S | 246 | 251 | Fe3 S4 | 295.78 | FE3-S4 CLUSTER |
| FUL | 187 | 240 | C6 H12 O5 | 164.16 | BETA-L-FUCOSE |
| OCS | 204 | 237 | C3 H7 N O5 S | 169.15 | CYSTEINESULFONIC ACID |
| DVA | 88 | 236 | C5 H11 N O2 | 117.15 | D-VALINE |
| U5P | 151 | 233 | C9 H13 N2 O9 P | 324.18 | URIDINE-5'-MONOPHOSPHATE |
| BTN | 163 | 233 | C10 H16 N2 O3 S | 244.31 | BIOTIN |
| MLA | 148 | 232 | C3 H4 O4 | 104.06 | MALONIC ACID |
| DTP | 161 | 229 | C10 H16 N5 O12 P3 | 491.18 | 2'-DEOXYADENOSINE 5'-TRIPHOSPHATE |
| HEZ | 93 | 228 | C6 H14 O2 | 118.18 | HEXANE-1,6-DIOL |
| PHE | 171 | 226 | C9 H11 N O2 | 165.19 | PHENYLALANINE |
| BEZ | 153 | 225 | C7 H6 O2 | 122.12 | BENZOIC ACID |
| A2G | 160 | 225 | C8 H15 N O6 | 221.21 | N-ACETYL-2-DEOXY-2-AMINO-GALACTOSE |
| NLE | 148 | 222 | C6 H13 N O2 | 131.17 | NORLEUCINE |
| LYS | 163 | 220 | C6 H15 N2 O2 1 | 147.2 | LYSINE |
| NO2 | 126 | 219 | N O2 -1 | 46.01 | NITRITE ION |
| MDO | 99 | 215 | C8 H13 N3 O3 | 197.19 | {2-[(1S)-1-AMINOETHYL]-5-HYDROXY-4-METHYL-1H-IMIDAZOL-1-YL}ACETIC ACID |
| PT | 103 | 213 | PT 2 | 195.09 | PLATINUM (II) ION |
| TAR | 151 | 212 | C4 H6 O6 | 150.09 | D(-)-TARTARIC ACID |
| DUR | 87 | 212 | C9 H12 N2 O5 | 228.2 | 2'-DEOXYURIDINE |
| BEF | 191 | 211 | Be F3 -1 | 66.01 | BERYLLIUM TRIFLUORIDE ION |
| 5GP | 149 | 210 | C10 H14 N5 O8 P | 363.22 | GUANOSINE-5'-MONOPHOSPHATE |
| IDS | 72 | 209 | C6 H10 O10 S | 274.2 | 2-O-SULFO-ALPHA-L-IDOPYRANURONIC ACID |
| TYD | 145 | 207 | C10 H16 N2 O11 P2 | 402.19 | THYMIDINE-5'-DIPHOSPHATE |
| PEK | 96 | 204 | C43 H78 N O8 P | 768.07 | (1S)-2-{[(2-AMINOETHOXY)(HYDROXY)PHOSPHORYL]OXY}-1-[(STEAROYLOXY)METHYL]ETHYL (5E,8E,11E,14E)-ICOSA-5,8,11,14-TETRAENOATE |
| PMP | 124 | 200 | C8 H13 N2 O5 P | 248.18 | 4'-DEOXY-4'-AMINOPYRIDOXAL-5'-PHOSPHATE |
| TGL | 108 | 198 | C57 H110 O6 | 891.49 | TRISTEAROYLGLYCEROL |
| TTP | 129 | 196 | C10 H17 N2 O14 P3 | 482.17 | THYMIDINE-5'-TRIPHOSPHATE |
| MOH | 42 | 195 | C H4 O | 32.04 | METHANOL |
| BTB | 150 | 195 | C8 H19 N O5 | 209.24 | 2-[BIS-(2-HYDROXY-ETHYL)-AMINO]-2-HYDROXYMETHYL-PROPANE-1,3-DIOL |
| ADN | 161 | 193 | C10 H13 N5 O4 | 267.24 | ADENOSINE |
| BMP | 97 | 191 | C9 H13 N2 O10 P | 340.18 | 6-HYDROXYURIDINE-5'-PHOSPHATE |
| H2U | 86 | 190 | C9 H15 N2 O9 P | 326.2 | 5,6-DIHYDROURIDINE-5'-MONOPHOSPHATE |
| F6P | 115 | 189 | C6 H13 O9 P | 260.14 | FRUCTOSE-6-PHOSPHATE |
| MMA | 145 | 188 | C7 H14 O6 | 194.18 | O1-METHYL-MANNOSE |
| MAL | 146 | 188 | C12 H22 O11 | 342.3 | MALTOSE |
| HIS | 116 | 188 | C6 H10 N3 O2 1 | 156.16 | HISTIDINE |
| CAS | 70 | 188 | C5 H12 AS N O2 S | 225.14 | S-(DIMETHYLARSENIC)CYSTEINE |
| SO3 | 128 | 185 | O3 S -2 | 80.06 | SULFITE ION |
| NGA | 148 | 184 | C8 H15 N O6 | 221.21 | N-ACETYL-D-GALACTOSAMINE |
| XYS | 91 | 183 | C5 H10 O5 | 150.13 | XYLOPYRANOSE |
| B12 | 142 | 183 | C62 H89 CO N13 O14 P 2 | 1330.37 | COBALAMIN |
| APC | 137 | 182 | C11 H18 N5 O12 P3 | 505.21 | DIPHOSPHOMETHYLPHOSPHONIC ACID ADENOSYL ESTER |
| ALF | 136 | 182 | AL F4 -1 | 102.97 | TETRAFLUOROALUMINATE ION |
| ASP | 124 | 181 | C4 H7 N O4 | 133.1 | ASPARTIC ACID |
| DGT | 108 | 180 | C10 H16 N5 O13 P3 | 507.18 | 2'-DEOXYGUANOSINE-5'-TRIPHOSPHATE |
| AU | 89 | 179 | AU 1 | 196.97 | GOLD ION |
| DPN | 176 | 178 | C9 H11 N O2 | 165.19 | D-PHENYLALANINE |
| TFP | 31 | 177 | C21 H24 F3 N3 S | 407.5 | 10-[3-(4-METHYL-PIPERAZIN-1-YL)-PROPYL]-2-TRIFLUOROMETHYL-10H-PHENOTHIAZINE |
| PEP | 163 | 177 | C3 H5 O6 P | 168.04 | PHOSPHOENOLPYRUVATE |
| HEA | 122 | 177 | C49 H56 FE N4 O6 | 852.85 | HEME-A |
| HTG | 89 | 176 | C13 H26 O5 S | 294.4 | HEPTYL 1-THIOHEXOPYRANOSIDE |
| HED | 140 | 174 | C4 H10 O2 S2 | 154.24 | 2-HYDROXYETHYL DISULFIDE |
| AIB | 63 | 173 | C4 H9 N O2 | 103.12 | ALPHA-AMINOISOBUTYRIC ACID |
| PHO | 86 | 172 | C55 H74 N4 O5 | 871.21 | PHEOPHYTIN A |
| WO4 | 100 | 170 | O4 W -2 | 247.85 | TUNGSTATE(VI)ION |
| FRU | 94 | 169 | C6 H12 O6 | 180.16 | FRUCTOSE |
| 5MU | 157 | 169 | C10 H15 N2 O9 P | 338.21 | 5-METHYLURIDINE 5'-MONOPHOSPHATE |
| NHE | 105 | 168 | C8 H17 N O3 S | 207.29 | 2-[N-CYCLOHEXYLAMINO]ETHANE SULFONIC ACID |
| F09 | 77 | 168 | C9 H20 O | 144.26 | NONAN-1-OL |
| DGL | 150 | 166 | C5 H9 N O4 | 147.13 | D-GLUTAMIC ACID |
| 5BU | 89 | 166 | C9 H12 BR N2 O9 P | 403.08 | 5-BROMO-URIDINE-5'-MONOPHOSPHATE |
| ACP | 140 | 164 | C11 H18 N5 O12 P3 | 505.21 | PHOSPHOMETHYLPHOSPHONIC ACID ADENYLATE ESTER |
| TRD | 34 | 162 | C13 H28 | 184.36 | TRIDECANE |
| HTO | 81 | 162 | C7 H16 O3 | 148.2 | HEPTANE-1,2,3-TRIOL |
| CTP | 125 | 161 | C9 H16 N3 O14 P3 | 483.16 | CYTIDINE-5'-TRIPHOSPHATE |
| C2E | 70 | 160 | C20 H24 N10 O14 P2 | 690.42 | 9,9'-[(2R,3R,3AS,5S,7AR,9R,10R,10AS,12S,14AR)-3,5,10,12-TETRAHYDROXY-5,12-DIOXIDOOCTAHYDRO-2H,7H-DIFURO[3,2-D:3',2'-J][1,3,7,9,2,8]TETRAOXADIPHOSPHACYCLODODECINE-2,9-DIYL]BIS(2-AMINO-1,9-DIHYDRO-6H-PURIN-6-ONE) |
| MGD | 80 | 157 | C20 H26 N10 O13 P2 S2 | 740.55 | 2-AMINO-5,6-DIMERCAPTO-7-METHYL-3,7,8A,9-TETRAHYDRO-8-OXA-1,3,9,10-TETRAAZA-ANTHRACEN-4-ONE GUANOSINE DINUCLEOTIDE |
| BMT | 140 | 157 | C10 H19 N O3 | 201.26 | 4-METHYL-4-[(E)-2-BUTENYL]-4,N-METHYL-THREONINE |
| 5MC | 115 | 156 | C10 H16 N3 O8 P | 337.23 | 5-METHYLCYTIDINE-5'-MONOPHOSPHATE |
| CHT | 45 | 155 | C5 H14 N O 1 | 104.17 | CHOLINE ION |
| SRT | 90 | 154 | C4 H6 O6 | 150.09 | S,R MESO-TARTARIC ACID |
| PB | 70 | 154 | PB 2 | 207.2 | LEAD (II) ION |
| CAA | 89 | 153 | C25 H40 N7 O18 P3 S | 851.61 | ACETOACETYL-COENZYME A |
| NRQ | 150 | 150 | C16 H17 N3 O4 S | 347.39 | {(4Z)-4-(4-HYDROXYBENZYLIDENE)-2-[3-(METHYLTHIO)PROPANIMIDOYL]-5-OXO-4,5-DIHYDRO-1H-IMIDAZOL-1-YL}ACETIC ACID |
| INI | 73 | 148 | C9 H14 N4 O8 | 306.23 | 5-NITRO-6-RIBITYL-AMINO-2,4(1H,3H)-PYRIMIDINEDIONE |
| GLN | 105 | 148 | C5 H10 N2 O3 | 146.15 | GLUTAMINE |
| C5P | 101 | 148 | C9 H14 N3 O8 P | 323.2 | CYTIDINE-5'-MONOPHOSPHATE |
| UPG | 118 | 147 | C15 H24 N2 O17 P2 | 566.3 | URIDINE-5'-DIPHOSPHATE-GLUCOSE |
| KDO | 108 | 147 | C8 H14 O8 | 238.19 | 3-DEOXY-D-MANNO-OCT-2-ULOSONIC ACID |
| B3L | 46 | 147 | C7 H15 N O2 | 145.2 | (3S)-3-AMINO-5-METHYLHEXANOIC ACID |
| AGS | 112 | 147 | C10 H16 N5 O12 P3 S | 523.24 | PHOSPHOTHIOPHOSPHORIC ACID-ADENYLATE ESTER |
| UMQ | 73 | 144 | C23 H44 O11 | 496.59 | UNDECYL-MALTOSIDE |
| RAM | 75 | 144 | C6 H12 O5 | 164.16 | ALPHA-L-RHAMNOSE |
| PCW | 14 | 143 | C44 H85 N O8 P 1 | 787.13 | 1,2-DIOLEOYL-SN-GLYCERO-3-PHOSPHOCHOLINE |
| MPG | 34 | 143 | C21 H40 O4 | 356.55 | 1-MONOOLEOYL-RAC-GLYCEROL |
| PEO | 77 | 142 | H2 O2 | 34.01 | HYDROGEN PEROXIDE |
| ORN | 114 | 142 | C5 H12 N2 O2 | 132.16 | L-ORNITHINE |
| YB | 72 | 141 | YB 3 | 173.04 | YTTERBIUM (III) ION |
| APR | 119 | 141 | C15 H23 N5 O14 P2 | 559.32 | ADENOSINE-5-DIPHOSPHORIBOSE |
| SGN | 58 | 139 | C6 H13 N O11 S2 | 339.29 | N,O6-DISULFO-GLUCOSAMINE |
| OH | 114 | 139 | H O -1 | 17.01 | HYDROXIDE ION |
| 2CV | 20 | 139 | C18 H37 N O7 | 379.49 | HEGA-10 |
| 2AN | 47 | 139 | C16 H13 N O3 S | 299.34 | 8-ANILINO-1-NAPHTHALENE SULFONATE |
| PHQ | 127 | 138 | C8 H7 Cl O2 | 136.15 | benzyl chlorocarbonate |
| ADE | 125 | 138 | C5 H5 N5 | 135.13 | ADENINE |
| 2PE | 92 | 138 | C18 H38 O10 | 414.49 | NONAETHYLENE GLYCOL |
| CXS | 80 | 136 | C9 H19 N O3 S | 221.31 | 3-CYCLOHEXYL-1-PROPYLSULFONIC ACID |
| NO | 120 | 135 | N O | 30.01 | NITRIC OXIDE |
| THM | 87 | 134 | C10 H14 N2 O5 | 242.23 | THYMIDINE |
| MLZ | 101 | 134 | C7 H16 N2 O2 | 160.22 | N-METHYL-LYSINE |
| LEU | 110 | 133 | C6 H13 N O2 | 131.17 | LEUCINE |
| DOC | 131 | 133 | C9 H14 N3 O6 P | 291.2 | 2',3'-DIDEOXYCYTIDINE-5'-MONOPHOSPHATE |
| SRM | 88 | 132 | C42 H42 FE N4 O16 | 914.66 | SIROHEME |
| MET | 93 | 131 | C5 H11 N O2 S | 149.21 | METHIONINE |
| URA | 93 | 130 | C4 H4 N2 O2 | 112.09 | URACIL |
| PGA | 108 | 130 | C2 H5 O6 P | 156.03 | 2-PHOSPHOGLYCOLIC ACID |
| P3S | 68 | 130 | C5 H13 N2 O6 P S | 260.2 | L-METHIONINE-S-SULFOXIMINE PHOSPHATE |
| GTX | 67 | 130 | C16 H30 N3 O6 S 1 | 392.49 | S-HEXYLGLUTATHIONE |
| TDP | 97 | 129 | C12 H18 N4 O7 P2 S | 424.3 | THIAMIN DIPHOSPHATE |
| MVA | 116 | 129 | C6 H13 N O2 | 131.17 | N-METHYLVALINE |
| DPV | 32 | 129 | C17 H38 N O4 P | 351.46 | DODECYL 2-(TRIMETHYLAMMONIO)ETHYL PHOSPHATE |
| CHL | 16 | 129 | C55 H70 MG N4 O6 2 | 907.49 | CHLOROPHYLL B |
| BRU | 85 | 129 | C9 H12 BR N2 O8 P | 387.08 | 5-BROMO-2'-DEOXYURIDINE-5'-MONOPHOSPHATE |
| 8OG | 127 | 129 | C10 H14 N5 O8 P | 363.22 | 8-OXO-2'-DEOXY-GUANOSINE-5'-MONOPHOSPHATE |
| STA | 79 | 128 | C8 H17 N O3 | 175.23 | STATINE |
| DPR | 107 | 128 | C5 H9 N O2 | 115.13 | D-PROLINE |
| BLA | 92 | 128 | C33 H34 N4 O6 | 582.66 | BILIVERDINE IX ALPHA |
| TPQ | 126 | 126 | C9 H9 N O5 | 211.17 | 5-(2-CARBOXY-2-AMINOETHYL)-2-HYDROXY-1,4-BENZOQUINONE |
| CPS | 60 | 126 | C32 H58 N2 O7 S | 614.88 | 3-[(3-CHOLAMIDOPROPYL)DIMETHYLAMMONIO]-1-PROPANESULFONATE |
| BCB | 32 | 126 | C55 H72 MG N4 O6 2 | 909.5 | BACTERIOCHLOROPHYLL B |
| 2HP | 46 | 126 | H2 O4 P -1 | 96.99 | DIHYDROGENPHOSPHATE ION |
| VO4 | 109 | 124 | O4 V -3 | 114.94 | VANADATE ION |
| 15P | 62 | 124 | C69 H140 O35 | 1529.84 | POLYETHYLENE GLYCOL (N=34) |
| CXE | 11 | 123 | C20 H42 O6 | 378.55 | PENTAETHYLENE GLYCOL MONODECYL ETHER |
| CDP | 98 | 123 | C9 H15 N3 O11 P2 | 403.18 | CYTIDINE-5'-DIPHOSPHATE |
| GSP | 111 | 122 | C10 H16 N5 O13 P3 S | 539.24 | 5'-GUANOSINE-DIPHOSPHATE-MONOTHIOPHOSPHATE |
| ALA | 112 | 122 | C3 H7 N O2 | 89.09 | ALANINE |
| CRO | 121 | 121 | C15 H17 N3 O5 | 319.32 | {2-[(1R,2R)-1-AMINO-2-HYDROXYPROPYL]-4-(4-HYDROXYBENZYLIDENE)-5-OXO-4,5-DIHYDRO-1H-IMIDAZOL-1-YL}ACETIC ACID |
| BIL | 33 | 121 | C7 H15 N O2 | 145.2 | (3R,4S)-3-AMINO-4-METHYLHEXANOIC ACID |
| PE4 | 94 | 120 | C16 H34 O8 | 354.44 | 2-{2-[2-(2-{2-[2-(2-ETHOXY-ETHOXY)-ETHOXY]-ETHOXY}-ETHOXY)-ETHOXY]-ETHOXY}-ETHANOL |
| FBP | 107 | 120 | C6 H14 O12 P2 | 340.12 | BETA-FRUCTOSE-1,6-DIPHOSPHATE |
| UD1 | 102 | 118 | C17 H27 N3 O17 P2 | 607.36 | URIDINE-DIPHOSPHATE-N-ACETYLGLUCOSAMINE |
| OLA | 70 | 118 | C18 H34 O2 | 282.46 | OLEIC ACID |
| IMP | 102 | 118 | C10 H13 N4 O8 P | 348.21 | INOSINIC ACID |
| DAO | 85 | 118 | C12 H24 O2 | 200.32 | LAURIC ACID |
| PHB | 93 | 117 | C7 H6 O3 | 138.12 | P-HYDROXYBENZOIC ACID |
| LMU | 58 | 116 | C24 H46 O11 | 510.62 | DODECYL-ALPHA-D-MALTOSIDE |
| MLT | 98 | 115 | C4 H6 O5 | 133.08 | MALATE ION |
| ADA | 31 | 115 | C6 H10 O7 | 194.14 | ALPHA-D-GALACTOPYRANURONIC ACID |
| A3P | 107 | 115 | C10 H15 N5 O10 P2 | 427.2 | ADENOSINE-3'-5'-DIPHOSPHATE |
| CYS | 88 | 114 | C3 H7 N O2 S | 121.15 | CYSTEINE |
| AG | 37 | 114 | AG 1 | 107.87 | SILVER ION |
| MAA | 113 | 113 | C4 H9 N O2 | 103.12 | N-METHYL-L-ALANINE |
| CSS | 99 | 113 | C3 H7 N O2 S2 | 153.21 | S-MERCAPTOCYSTEINE |
| MP8 | 112 | 112 | C6 H11 N O2 | 129.16 | (4R)-4-METHYL-L-PROLINE |
| HDD | 60 | 112 | C34 H32 FE N4 O5 | 632.5 | CIS-HEME D HYDROXYCHLORIN GAMMA-SPIROLACTONE |
| 7MG | 106 | 111 | C11 H18 N5 O8 P | 379.27 | 7N-METHYL-8-HYDROGUANOSINE-5'-MONOPHOSPHATE |
| AF3 | 107 | 110 | AL F3 | 83.98 | ALUMINUM FLUORIDE |
| SAC | 80 | 109 | C5 H9 N O4 | 147.13 | N-ACETYL-SERINE |
| HCA | 109 | 109 | C7 H10 O7 | 206.15 | 3-HYDROXY-3-CARBOXY-ADIPIC ACID |
| 3PG | 88 | 109 | C3 H7 O7 P | 186.06 | 3-PHOSPHOGLYCERIC ACID |
| MGM | 108 | 108 | C19 H37 N O7 P2 | 453.45 | 2-[METHYL-(5-GERANYL-4-METHYL-PENT-3-ENYL)-AMINO]-ETHYL-DIPHOSPHATE |
| MEA | 62 | 108 | C10 H13 N O2 | 179.22 | N-METHYLPHENYLALANINE |
| LAT | 95 | 108 | C12 H22 O11 | 342.3 | BETA-LACTOSE |
| 13P | 107 | 108 | C3 H7 O6 P | 170.06 | 1,3-DIHYDROXYACETONEPHOSPHATE |
| SER | 89 | 107 | C3 H7 N O3 | 105.09 | SERINE |
| RBF | 64 | 107 | C17 H20 N4 O6 | 376.37 | RIBOFLAVIN |
| FEO | 100 | 107 | FE2 O | 127.69 | MU-OXO-DIIRON |
| YT3 | 69 | 106 | Y 3 | 88.91 | YTTRIUM (III) ION |
| PLC | 61 | 106 | C32 H65 N O8 P 1 | 622.84 | DIUNDECYL PHOSPHATIDYL CHOLINE |
| OCT | 47 | 106 | C8 H18 | 114.23 | N-OCTANE |
| IPT | 71 | 106 | C9 H18 O5 S | 238.3 | ISOPROPYL-1-BETA-D-THIOGALACTOSIDE |
| CRS | 52 | 106 | C7 H8 O | 108.14 | M-CRESOL |
| WFP | 70 | 105 | C9 H9 F2 N O2 | 201.17 | 3,5-DIFLUORO-L-PHENYLALANINE |
| OTT | 70 | 105 | C8 H10 O2 | 138.17 | (2E,4E,6E)-OCTA-2,4,6-TRIENOIC ACID |
| JHM | 20 | 105 | C6 H12 O8 S | 244.22 | 2-DEOXY-6-O-SULFO-ALPHA-D-ARABINO-HEXOPYRANOSE |
| HIC | 92 | 105 | C7 H11 N3 O2 | 169.18 | 4-METHYL-HISTIDINE |
| HC4 | 69 | 105 | C9 H8 O3 | 164.16 | 4'-HYDROXYCINNAMIC ACID |
| AHR | 41 | 105 | C5 H10 O5 | 150.13 | ALPHA-L-ARABINOFURANOSE |
| URI | 63 | 104 | C9 H12 N2 O6 | 244.2 | URIDINE |
| MTX | 92 | 104 | C20 H22 N8 O5 | 454.44 | METHOTREXATE |
| GUN | 75 | 104 | C5 H5 N5 O | 151.13 | GUANINE |
| DCP | 86 | 104 | C9 H16 N3 O13 P3 | 467.16 | 2'-DEOXYCYTIDINE-5'-TRIPHOSPHATE |
| TAM | 83 | 103 | C7 H17 N O3 | 163.22 | TRIS(HYDROXYETHYL)AMINOMETHANE |
| MIA | 92 | 103 | C16 H26 N5 O7 P S | 463.44 | 2-METHYLTHIO-N6-ISOPENTENYL-ADENOSINE-5'-MONOPHOSPHATE |
| 3DR | 89 | 103 | C5 H11 O6 P | 198.11 | 1',2'-DIDEOXYRIBOFURANOSE-5'-PHOSPHATE |
| RB | 46 | 102 | RB 1 | 85.47 | RUBIDIUM ION |
| DUT | 50 | 102 | C9 H15 N2 O14 P3 | 468.14 | DEOXYURIDINE-5'-TRIPHOSPHATE |
| CLF | 102 | 102 | FE8 S7 | 671.2 | FE(8)-S(7) CLUSTER |
| 1MA | 85 | 102 | C11 H16 N5 O7 P | 361.25 | 6-HYDRO-1-METHYLADENOSINE-5'-MONOPHOSPHATE |
| U | 54 | 101 | C9 H13 N2 O9 P | 324.18 | URIDINE-5'-MONOPHOSPHATE |
| TYR | 92 | 101 | C9 H11 N O3 | 181.19 | TYROSINE |
| PRP | 85 | 101 | C5 H13 O14 P3 | 390.07 | ALPHA-PHOSPHORIBOSYLPYROPHOSPHORIC ACID |
| MTE | 101 | 101 | C10 H14 N5 O6 P S2 | 395.34 | PHOSPHONIC ACIDMONO-(2-AMINO-5,6-DIMERCAPTO-4-OXO-3,7,8A,9,10,10A-HEXAHYDRO-4H-8-OXA-1,3,9,10-TETRAAZA-ANTHRACEN-7-YLMETHYL)ESTER |
| CRQ | 101 | 101 | C16 H16 N4 O5 | 344.33 | [2-(3-CARBAMOYL-1-IMINO-PROPYL)-4-(4-HYDROXY-BENZYLIDENE)-5-OXO-4,5-DIHYDRO-IMIDAZOL-1-YL]-ACETIC ACID |
| PE5 | 43 | 100 | C18 H38 O9 | 398.49 | 3,6,9,12,15,18,21,24-OCTAOXAHEXACOSAN-1-OL |
| SAL | 63 | 99 | C7 H6 O3 | 138.12 | 2-HYDROXYBENZOIC ACID |
| IPE | 79 | 99 | C5 H12 O7 P2 | 246.09 | 3-METHYLBUT-3-ENYL TRIHYDROGEN DIPHOSPHATE |
| 1PG | 79 | 99 | C11 H24 O6 | 252.31 | 2-(2-{2-[2-(2-METHOXY-ETHOXY)-ETHOXY]-ETHOXY}-ETHOXY)-ETHANOL |
| CXM | 98 | 98 | C6 H11 N O4 S | 193.22 | N-CARBOXYMETHIONINE |
| VAL | 90 | 97 | C5 H11 N O2 | 117.15 | VALINE |
| TRE | 59 | 97 | C12 H22 O11 | 342.3 | TREHALOSE |
| TMP | 80 | 97 | C10 H15 N2 O8 P | 322.21 | THYMIDINE-5'-PHOSPHATE |
| PC1 | 52 | 97 | C44 H88 N O8 P | 790.16 | 1,2-DIACYL-SN-GLYCERO-3-PHOSPHOCHOLINE |
| IRI | 12 | 97 | H18 IR N6 3 | 294.4 | IRIDIUM HEXAMMINE ION |
| F43 | 72 | 96 | C42 H49 N6 NI O13 | 904.58 | FACTOR 430 |
| UNK | 44 | 95 | C4 H9 N O2 | 103.12 | UNKNOWN |
| SMA | 95 | 95 | C30 H42 O7 | 514.66 | STIGMATELLIN A |
| P33 | 74 | 95 | C14 H30 O8 | 326.39 | 3,6,9,12,15,18-HEXAOXAICOSANE-1,20-DIOL |
| OGA | 94 | 95 | C4 H5 N O5 | 147.09 | N-OXALYLGLYCINE |
| DHB | 77 | 95 | C7 H6 O4 | 154.12 | 3,4-DIHYDROXYBENZOIC ACID |
| CLR | 74 | 95 | C27 H46 O | 386.66 | CHOLESTEROL |
| B3P | 72 | 95 | C11 H26 N2 O6 | 282.34 | 2-[3-(2-HYDROXY-1,1-DIHYDROXYMETHYL-ETHYLAMINO)-PROPYLAMINO]-2-HYDROXYMETHYL-PROPANE-1,3-DIOL |
| ARF | 24 | 95 | C H3 N O | 45.04 | FORMAMIDE |
| MTA | 76 | 94 | C11 H15 N5 O3 S | 297.33 | 5'-DEOXY-5'-METHYLTHIOADENOSINE |
| MGE | 44 | 93 | C38 H72 O10 | 688.98 | (1S)-2-(ALPHA-L-ALLOPYRANOSYLOXY)-1-[(TRIDECANOYLOXY)METHYL]ETHYL PALMITATE |
| DUP | 51 | 93 | C9 H16 N3 O13 P3 | 467.16 | 2'-DEOXYURIDINE 5'-ALPHA,BETA-IMIDO-TRIPHOSPHATE |
| CB3 | 81 | 93 | C24 H23 N5 O6 | 477.48 | 10-PROPARGYL-5,8-DIDEAZAFOLIC ACID |
| ALC | 85 | 93 | C9 H17 N O2 | 171.24 | 2-AMINO-3-CYCLOHEXYL-PROPIONIC ACID |
| OMC | 87 | 92 | C10 H16 N3 O8 P | 337.23 | O2'-METHYLYCYTIDINE-5'-MONOPHOSPHATE |
| IT1 | 62 | 92 | C14 H22 N3 O7 P | 375.32 | (2S)-2-AMINO-6-[[3-HYDROXY-2-METHYL-5-(PHOSPHONOOXYMETHYL)PYRIDIN-4-YL]METHYLIDENEAMINO]HEXANOIC ACID |
| BOC | 86 | 92 | C5 H10 O3 | 118.13 | TERT-BUTYL HYDROGEN CARBONATE |
| 5IU | 39 | 92 | C9 H12 I N2 O8 P | 434.08 | 5-IODO-2'-DEOXYURIDINE-5'-MONOPHOSPHATE |
| HEX | 32 | 91 | C6 H14 | 86.18 | HEXANE |
| HBI | 59 | 91 | C9 H13 N5 O3 | 239.23 | 7,8-DIHYDROBIOPTERIN |
| GHP | 32 | 91 | C8 H9 N O3 | 167.16 | (2R)-AMINO(4-HYDROXYPHENYL)ETHANOIC ACID |
| DSN | 71 | 91 | C3 H7 N O3 | 105.09 | D-SERINE |
| ARS | 61 | 91 | AS | 74.92 | ARSENIC |
| O | 71 | 90 | O | 16 | OXYGEN ATOM |
| MEN | 90 | 90 | C5 H10 N2 O3 | 146.15 | N-METHYL ASPARAGINE |
| DCY | 60 | 90 | C3 H7 N O2 S | 121.15 | D-CYSTEINE |
| CBI | 75 | 90 | C12 H22 O11 | 342.3 | CELLOBIOSE |
| MPO | 74 | 89 | C7 H15 N O4 S | 209.26 | 3[N-MORPHOLINO]PROPANE SULFONIC ACID |
| COM | 80 | 89 | C2 H6 O3 S2 | 142.19 | 1-THIOETHANESULFONIC ACID |
| UFP | 52 | 88 | C9 H12 F N2 O8 P | 326.17 | 5-FLUORO-2'-DEOXYURIDINE-5'-MONOPHOSPHATE |
| GCP | 75 | 88 | C11 H18 N5 O13 P3 | 521.21 | PHOSPHOMETHYLPHOSPHONIC ACID GUANYLATE ESTER |
| DTR | 53 | 88 | C11 H12 N2 O2 | 204.23 | D-TRYPTOPHAN |
| ASA | 88 | 88 | C4 H7 N O3 | 117.1 | ASPARTIC ALDEHYDE |
| TBU | 50 | 87 | C4 H10 O | 74.12 | TERTIARY-BUTYL ALCOHOL |
| LMZ | 40 | 87 | C9 H14 N4 O7 | 290.23 | 5-NITROSO-6-RIBITYL-AMINO-2,4(1H,3H)-PYRIMIDINEDIONE |
| CR2 | 87 | 87 | C13 H13 N3 O4 | 275.26 | {(4Z)-2-(AMINOMETHYL)-4-[(4-HYDROXYPHENYL)METHYLIDENE]-5-OXO-4,5-DIHYDRO-1H-IMIDAZOL-1-YL}ACETIC ACID |
| BU3 | 35 | 87 | C4 H10 O2 | 90.12 | (R,R)-2,3-BUTANEDIOL |
| P4C | 52 | 86 | C14 H28 O8 | 324.37 | O-ACETALDEHYDYL-HEXAETHYLENE GLYCOL |
| GDU | 79 | 86 | C15 H24 N2 O17 P2 | 566.3 | GALACTOSE-URIDINE-5'-DIPHOSPHATE |
| RG1 | 30 | 85 | C46 H66 O6 | 715.02 | RHODOPIN GLUCOSIDE |
| DLY | 65 | 85 | C6 H14 N2 O2 | 146.19 | D-LYSINE |
| XCP | 31 | 84 | C6 H11 N O2 | 129.16 | (1S,2S)-2-AMINOCYCLOPENTANECARBOXYLIC ACID |
| UTP | 71 | 84 | C9 H15 N2 O15 P3 | 484.14 | URIDINE 5'-TRIPHOSPHATE |
| SFG | 78 | 84 | C15 H23 N7 O5 | 381.39 | SINEFUNGIN |
| PEB | 31 | 84 | C33 H40 N4 O6 | 588.7 | PHYCOERYTHROBILIN |
| BU1 | 42 | 84 | C4 H10 O2 | 90.12 | 1,4-BUTANEDIOL |
| 08T | 48 | 84 | C10 H14 BE F3 N5 O10 P2 | 492.2 | [[[(2R,3S,4R,5R)-5-(6-AMINOPURIN-9-YL)-3,4-BIS(OXIDANYL)OXOLAN-2-YL]METHOXY-OXIDANYL-PHOSPHORYL]OXY-OXIDANYL-PHOSPHORYL]OXY-TRIS(FLUORANYL)BERYLLIUM |
| ZIL | 28 | 83 | C20 H30 N2 O5 | 378.46 | N-[(benzyloxy)carbonyl]-L-isoleucyl-L-leucine |
| UP6 | 47 | 83 | C8 H12 N3 O9 P | 325.17 | 6-AZA URIDINE 5'-MONOPHOSPHATE |
| SM | 44 | 83 | SM 3 | 150.4 | SAMARIUM (III) ION |
| OPC | 58 | 83 | C45 H87 N O8 P 1 | 801.16 | (7R,17E)-4-HYDROXY-N,N,N,7-TETRAMETHYL-7-[(8E)-OCTADEC-8-ENOYLOXY]-10-OXO-3,5,9-TRIOXA-4-PHOSPHAHEPTACOS-17-EN-1-AMINIUM 4-OXIDE |
| OAA | 61 | 83 | C4 H3 O5 -1 | 131.07 | OXALOACETATE ION |
| F | 43 | 83 | F -1 | 19 | FLUORIDE ION |
| 5AD | 65 | 83 | C10 H13 N5 O3 | 251.24 | 5'-DEOXYADENOSINE |
| PLG | 45 | 82 | C10 H15 N2 O7 P | 306.21 | N-GLYCINE-[3-HYDROXY-2-METHYL-5-PHOSPHONOOXYMETHYL-PYRIDIN-4-YL-METHANE] |
| GPP | 67 | 82 | C10 H20 O7 P2 | 314.21 | GERANYL DIPHOSPHATE |
| 0QE | 80 | 82 | C H3 CL | 50.49 | CHLOROMETHANE |
| PRO | 66 | 81 | C5 H9 N O2 | 115.13 | PROLINE |
| OMG | 66 | 81 | C11 H16 N5 O8 P | 377.25 | O2'-METHYLGUANOSINE-5'-MONOPHOSPHATE |
| CR8 | 81 | 81 | C17 H16 N5 O4 -1 | 354.34 | 2-[1-AMINO-2-(1H-IMIDAZOL-5-YL)ETHYL]-1-(CARBOXYMETHYL)-4-[(4-OXOCYCLOHEXA-2,5-DIEN-1-YLIDENE)METHYL]-1H-IMIDAZOL-5-OLATE |
| BHG | 62 | 81 | C12 H24 O6 | 264.32 | 2-HEXYLOXY-6-HYDROXYMETHYL-TETRAHYDRO-PYRAN-3,4,5-TRIOL |
| TCL | 79 | 80 | C12 H7 CL3 O2 | 289.55 | TRICLOSAN |
| PEV | 8 | 80 | C39 H78 N O8 P | 720.02 | (1S)-2-{[(2-AMINOETHOXY)(HYDROXY)PHOSPHORYL]OXY}-1-[(PALMITOYLOXY)METHYL]ETHYL STEARATE |
| BPB | 44 | 80 | C55 H76 N4 O6 | 887.21 | BACTERIOPHEOPHYTIN B |
| THP | 78 | 79 | C10 H16 N2 O11 P2 | 402.19 | THYMIDINE-3',5'-DIPHOSPHATE |
| G6P | 53 | 79 | C6 H13 O9 P | 260.14 | ALPHA-D-GLUCOSE-6-PHOSPHATE |
| ETA | 48 | 79 | C2 H7 N O | 61.08 | ETHANOLAMINE |
| 12P | 55 | 79 | C24 H50 O13 | 546.65 | DODECAETHYLENE GLYCOL |
| MAE | 48 | 78 | C4 H4 O4 | 116.07 | MALEIC ACID |
| B3K | 29 | 78 | C7 H16 N2 O2 | 160.22 | (3S)-3,7-DIAMINOHEPTANOIC ACID |
| 4SU | 78 | 78 | C9 H13 N2 O8 P S | 340.24 | 4-THIOURIDINE-5'-MONOPHOSPHATE |
| 2GP | 66 | 78 | C10 H14 N5 O8 P | 363.22 | GUANOSINE-2'-MONOPHOSPHATE |
| TRQ | 77 | 77 | C11 H10 N2 O4 | 234.21 | 2-AMINO-3-(6,7-DIOXO-6,7-DIHYDRO-1H-INDOL-3-YL)-PROPIONIC ACID |
| CM5 | 32 | 77 | C23 H42 O11 | 494.58 | 5-CYCLOHEXYL-1-PENTYL-BETA-D-MALTOSIDE |
| AZA | 57 | 77 | C4 H3 N5 O2 | 153.1 | 8-AZAXANTHINE |
| GYC | 76 | 76 | C14 H15 N3 O4 S | 321.35 | [(4Z)-2-[(1R)-1-AMINO-2-MERCAPTOETHYL]-4-(4-HYDROXYBENZYLIDENE)-5-OXO-4,5-DIHYDRO-1H-IMIDAZOL-1-YL]ACETIC ACID |
| B3E | 39 | 76 | C6 H11 N O4 | 161.16 | (3S)-3-AMINOHEXANEDIOIC ACID |
| 3CO | 56 | 76 | CO 3 | 58.93 | COBALT (III) ION |
| PGH | 55 | 75 | C2 H6 N O6 P | 171.05 | PHOSPHOGLYCOLOHYDROXAMIC ACID |
| IVA | 73 | 75 | C5 H10 O2 | 102.13 | ISOVALERIC ACID |
| DKA | 50 | 75 | C10 H20 O2 | 172.27 | DECANOIC ACID |
| 1MG | 75 | 75 | C11 H16 N5 O8 P | 377.25 | 1N-METHYLGUANOSINE-5'-MONOPHOSPHATE |
| PGW | 11 | 74 | C40 H77 O10 P | 749.02 | (1R)-2-{[(S)-{[(2S)-2,3-DIHYDROXYPROPYL]OXY}(HYDROXY)PHOSPHORYL]OXY}-1-[(HEXADECANOYLOXY)METHYL]ETHYL (9Z)-OCTADEC-9-ENOATE |
| PE8 | 51 | 74 | C16 H34 O9 | 370.44 | 3,6,9,12,15,18,21-HEPTAOXATRICOSANE-1,23-DIOL |
| KPI | 74 | 74 | C9 H16 N2 O4 | 216.24 | (2S)-2-AMINO-6-[(1-HYDROXY-1-OXO-PROPAN-2-YLIDENE)AMINO]HEXANOIC ACID |
| GRG | 60 | 74 | C20 H36 O7 P2 | 450.45 | GERANYLGERANYL DIPHOSPHATE |
| FUM | 52 | 74 | C4 H4 O4 | 116.07 | FUMARIC ACID |
| FDA | 60 | 74 | C27 H35 N9 O15 P2 | 787.57 | DIHYDROFLAVINE-ADENINE DINUCLEOTIDE |
| DUD | 44 | 74 | C9 H14 N2 O11 P2 | 388.16 | DEOXYURIDINE-5'-DIPHOSPHATE |
| BB9 | 26 | 74 | C3 H5 N O2 S | 119.14 | (2Z)-2-AMINO-3-SULFANYLPROP-2-ENOIC ACID |
| WO2 | 42 | 73 | O62 P2 W18 | 4363.21 | OCTADECATUNGSTENYL DIPHOSPHATE |
| STU | 67 | 73 | C28 H26 N4 O3 | 466.54 | STAUROSPORINE |
| PER | 63 | 73 | O2 -2 | 32 | PEROXIDE ION |
| OZT | 40 | 73 | C5 H7 N O4 | 145.12 | (4S,5R)-5-METHYL-2-OXO-1,3-OXAZOLIDINE-4-CARBOXYLIC ACID |
| L3P | 21 | 73 | C46 H94 O11 P2 -2 | 885.19 | 2,3-DI-O-PHYTANLY-3-SN-GLYCERO-1-PHOSPHORYL-3'-SN-GLYCEROL-1'-PHOSPHATE |
| FOR | 63 | 73 | C H2 O | 30.03 | FORMYL GROUP |
| FA1 | 37 | 73 | C7 H10 O5 | 174.15 | 2,3 -ANHYDRO-QUINIC ACID |
| DHA | 40 | 73 | C3 H5 N O2 | 87.08 | 2-AMINO-ACRYLIC ACID |
| V7O | 32 | 72 | O19 V7 | 660.58 | META VANADATE |
| SPD | 57 | 72 | C7 H19 N3 | 145.25 | SPERMIDINE |
| PSC | 72 | 72 | C42 H81 N O8 P 1 | 759.08 | (7R,17E,20E)-4-HYDROXY-N,N,N-TRIMETHYL-9-OXO-7-[(PALMITOYLOXY)METHYL]-3,5,8-TRIOXA-4-PHOSPHAHEXACOSA-17,20-DIEN-1-AMINIUM 4-OXIDE |
| ORO | 68 | 72 | C5 H4 N2 O4 | 156.1 | OROTIC ACID |
| CH2 | 72 | 72 |  |  |  |
| PG6 | 44 | 71 | C12 H26 O6 | 266.33 | 1-(2-METHOXY-ETHOXY)-2-{2-[2-(2-METHOXY-ETHOXY]-ETHOXY}-ETHANE |
| PPV | 59 | 70 | H4 O7 P2 | 177.98 | PYROPHOSPHATE |
| PLR | 44 | 70 | C8 H12 N O5 P | 233.16 | (5-HYDROXY-4,6-DIMETHYLPYRIDIN-3-YL)METHYL DIHYDROGEN PHOSPHATE |
| 5RP | 56 | 70 | C5 H11 O8 P | 230.11 | RIBULOSE-5-PHOSPHATE |
| PPU | 69 | 69 | C22 H30 N7 O8 P | 551.49 | PUROMYCIN-5'-MONOPHOSPHATE |
| DTY | 44 | 69 | C9 H11 N O3 | 181.19 | D-TYROSINE |
| CAM | 69 | 69 | C10 H16 O | 152.24 | CAMPHOR |
| ASN | 51 | 69 | C4 H8 N2 O3 | 132.12 | ASPARAGINE |
| PNS | 58 | 68 | C11 H23 N2 O7 P S | 358.35 | 4'-PHOSPHOPANTETHEINE |
| NIY | 61 | 68 | C9 H10 N2 O5 | 226.19 | META-NITRO-TYROSINE |
| FPP | 64 | 68 | C15 H28 O7 P2 | 382.33 | FARNESYL DIPHOSPHATE |
| CUA | 68 | 68 | CU2 | 127.09 | DINUCLEAR COPPER ION |
| BGL | 47 | 68 | C14 H28 O6 | 292.37 | B-2-OCTYLGLUCOSIDE |
| REA | 57 | 67 | C20 H28 O2 | 300.44 | RETINOIC ACID |
| LYZ | 12 | 67 | C6 H14 N2 O3 | 162.19 | 5-HYDROXYLYSINE |
| ICT | 47 | 67 | C6 H8 O7 | 192.12 | ISOCITRIC ACID |
| FOL | 65 | 67 | C19 H19 N7 O6 | 441.4 | FOLIC ACID |
| DTU | 54 | 67 | C4 H10 O2 S2 | 154.24 | (2R,3S)-1,4-DIMERCAPTOBUTANE-2,3-DIOL |
| BCN | 50 | 67 | C6 H13 N O4 | 163.17 | BICINE |
| BCD | 44 | 67 | C42 H70 O35 | 1134.99 | BETA-CYCLODEXTRIN |
| AG2 | 46 | 67 | C5 H14 N4 | 130.19 | AGMATINE |
| TP7 | 44 | 66 | C11 H22 N O7 P S | 343.33 | COENZYME B |
| OXM | 65 | 66 | C2 H3 N O3 | 89.05 | OXAMIC ACID |
| CCN | 23 | 66 | C2 H3 N | 41.05 | ACETONITRILE |
| AGM | 44 | 66 | C7 H17 N4 O2 1 | 189.24 | 5-METHYL-ARGININE |
| EMC | 36 | 65 | C2 H5 HG 1 | 229.65 | ETHYL MERCURY ION |
| CXP | 44 | 65 | C9 H16 O2 | 156.22 | CYCLOHEXANE PROPIONIC ACID |
| 2MG | 61 | 65 | C11 H16 N5 O8 P | 377.25 | 2N-METHYLGUANOSINE-5'-MONOPHOSPHATE |
| R5P | 55 | 64 | C5 H13 O8 P | 232.13 | RIBOSE-5-PHOSPHATE |
| PG0 | 35 | 64 | C5 H12 O3 | 120.15 | 2-(2-METHOXYETHOXY)ETHANOL |
| PEH | 18 | 64 | C41 H82 N O8 P | 748.07 | DI-STEAROYL-3-SN-PHOSPHATIDYLETHANOLAMINE |
| HAS | 64 | 64 | C54 H64 FE N4 O6 | 920.97 | HEME-AS |
| FNR | 48 | 64 | C17 H23 N4 O9 P | 458.36 | 1-DEOXY-1-(7,8-DIMETHYL-2,4-DIOXO-3,4-DIHYDRO-2H-BENZO[G]PTERIDIN-1-ID-10(5H)-YL)-5-O-PHOSPHONATO-D-RIBITOL |
| AZM | 56 | 64 | C4 H6 N4 O3 S2 | 222.24 | 5-ACETAMIDO-1,3,4-THIADIAZOLE-2-SULFONAMIDE |
| AYA | 40 | 64 | C5 H9 N O3 | 131.13 | N-ACETYLALANINE |
| ADX | 36 | 64 | C10 H14 N5 O10 P S | 427.28 | ADENOSINE-5'-PHOSPHOSULFATE |
| NMN | 47 | 63 | C11 H16 N2 O8 P 1 | 335.23 | BETA-NICOTINAMIDE RIBOSE MONOPHOSPHATE |
| BAL | 51 | 63 | C3 H7 N O2 | 89.09 | BETA-ALANINE |
| SCY | 42 | 62 | C5 H9 N O3 S | 163.19 | S-ACETYL-CYSTEINE |
| R1P | 34 | 62 | C5 H11 O8 P | 230.11 | RIBOSE-1-PHOSPHATE |
| PXX | 28 | 62 | C14 H11 N3 O | 237.26 | N-1,10-PHENANTHROLIN-5-YLACETAMIDE |
| PUT | 56 | 62 | C4 H12 N2 | 88.15 | 1,4-DIAMINOBUTANE |
| MLK | 30 | 62 | C37 H50 N2 O10 | 682.81 | METHYLLYCACONITINE |
| PTY | 32 | 61 | C40 H80 N O8 P | 734.05 | PHOSPHATIDYLETHANOLAMINE |
| DAN | 61 | 61 | C11 H17 N O8 | 291.26 | 2-DEOXY-2,3-DEHYDRO-N-ACETYL-NEURAMINIC ACID |
| D10 | 37 | 61 | C10 H22 | 142.28 | DECANE |
| API | 49 | 61 | C7 H14 N2 O4 | 190.2 | 2,6-DIAMINOPIMELIC ACID |
| NCA | 55 | 60 | C6 H6 N2 O | 122.13 | NICOTINAMIDE |
| FGA | 49 | 60 | C5 H9 N O4 | 147.13 | GAMMA-D-GLUTAMIC ACID |
| A2P | 42 | 60 | C10 H15 N5 O10 P2 | 427.2 | ADENOSINE-2'-5'-DIPHOSPHATE |
| YOF | 5 | 59 | C9 H10 F N O3 | 199.18 | 3-FLUOROTYROSINE |
| SPM | 56 | 59 | C10 H26 N4 | 202.34 | SPERMINE |
| PID | 9 | 59 | C39 H50 O7 | 630.82 | PERIDININ |
| NCN | 39 | 59 | C11 H14 N O9 P | 335.21 | NICOTINATE MONONUCLEOTIDE |
| MA4 | 30 | 59 | C24 H44 O11 | 508.61 | CYCLOHEXYL-HEXYL-BETA-D-MALTOSIDE |
| FDP | 38 | 59 | C6 H14 O12 P2 | 340.12 | FRUCTOSE-2,6-DIPHOSPHATE |
| 78M | 15 | 59 | C18 H34 O4 | 314.46 | (2S)-2,3-DIHYDROXYPROPYL(7Z)-PENTADEC-7-ENOATE |
| IAS | 58 | 58 | C4 H7 N O4 | 133.1 | BETA-L-ASPARTIC ACID |
| ETF | 31 | 58 | C2 H3 F3 O | 100.04 | TRIFLUOROETHANOL |
| ABU | 36 | 58 | C4 H9 N O2 | 103.12 | GAMMA-AMINO-BUTANOIC ACID |
| YG | 44 | 57 | C21 H29 N6 O12 P | 588.47 | WYBUTOSINE |
| OPE | 51 | 57 | C2 H8 N O4 P | 141.06 | PHOSPHORIC ACID MONO-(2-AMINO-ETHYL) ESTER |
| MQ7 | 53 | 57 | C46 H64 O2 | 649.01 | MENAQUINONE-7 |
| H2S | 42 | 57 | H2 S | 34.08 | HYDROSULFURIC ACID |
| CP | 37 | 57 | C H4 N O5 P | 141.02 | PHOSPHORIC ACID MONO(FORMAMIDE)ESTER |
| SLB | 30 | 56 | C11 H19 N O9 | 309.27 | 5-N-ACETYL-BETA-D-NEURAMINIC ACID |
| RNS | 32 | 56 | C6 H12 O5 | 164.16 | L-RHAMNOSE |
| MOS | 56 | 56 | H MO O2 S | 161.01 | DIOXOTHIOMOLYBDENUM(VI) ION |
| DHE | 42 | 56 | C34 H32 FE N4 O10 | 712.49 | HEME D |
| DCM | 27 | 56 | C9 H14 N3 O7 P | 307.2 | 2'-DEOXYCYTIDINE-5'-MONOPHOSPHATE |
| DAR | 43 | 56 | C6 H15 N4 O2 1 | 175.21 | D-ARGININE |
| 5CM | 45 | 56 | C10 H16 N3 O7 P | 321.23 | 5-METHYL-2'-DEOXY-CYTIDINE-5'-MONOPHOSPHATE |
| XYL | 40 | 55 | C5 H12 O5 | 152.15 | D-XYLITOL |
| SVR | 17 | 55 | C51 H40 N6 O23 S6 | 1297.26 | 8,8'-[CARBONYLBIS[IMINO-3,1-PHENYLENECARBONYLIMINO(4-METHYL-3,1-PHENYLENE)CARBONYLIMINO]]BIS-1,3,5-NAPHTHALENETRISULFONIC ACID |
| SOG | 19 | 55 | C14 H28 O5 S | 308.43 | 2-HYDROXYMETHYL-6-OCTYLSULFANYL-TETRAHYDRO-PYRAN-3,4,5-TRIOL |
| O12 | 12 | 55 | C17 H34 N2 O3 | 314.47 | N~5~-DODECANOYL-L-ORNITHINE |
| MOO | 34 | 55 | MO O4 -2 | 159.94 | MOLYBDATE ION |
| DXC | 32 | 55 | C24 H40 O4 | 392.58 | (3ALPHA,5BETA,12ALPHA)-3,12-DIHYDROXYCHOLAN-24-OIC ACID |
| GER | 54 | 54 | C20 H34 | 274.49 | GERAN-8-YL GERAN |
| BE2 | 30 | 54 | C7 H7 N O2 | 137.14 | 2-AMINOBENZOIC ACID |
| AP5 | 52 | 54 | C20 H29 N10 O22 P5 | 916.37 | BIS(ADENOSINE)-5'-PENTAPHOSPHATE |
| ACH | 31 | 54 | C7 H16 N O2 1 | 146.21 | ACETYLCHOLINE |
| STL | 36 | 53 | C14 H12 O3 | 228.25 | RESVERATROL |
| OS | 9 | 53 | OS 3 | 190.2 | OSMIUM ION |
| LU | 32 | 53 | LU 3 | 174.97 | LUTETIUM (III) ION |
| HPA | 53 | 53 | C5 H4 N4 O | 136.11 | HYPOXANTHINE |
| G3H | 49 | 53 | C3 H7 O6 P | 170.06 | GLYCERALDEHYDE-3-PHOSPHATE |
| G2P | 51 | 53 | C11 H18 N5 O13 P3 | 521.21 | PHOSPHOMETHYLPHOSPHONIC ACID GUANYLATE ESTER |
| B3N | 31 | 53 | C16 H25 N3 O3 | 307.39 | 4-(DIMETHYLAMINO)-N-[7-(HYDROXYAMINO)-7-OXOHEPTYL]BENZAMIDE |
| SGM | 46 | 52 | C3 H8 O2 S | 108.15 | MONOTHIOGLYCEROL |
| PMS | 43 | 52 | C7 H8 O3 S | 172.2 | PHENYLMETHANESULFONIC ACID |
| PHI | 38 | 52 | C9 H10 I N O2 | 291.09 | IODO-PHENYLALANINE |
| P5P | 24 | 52 | C10 H13 N4 O7 P | 332.21 | PURINE RIBOSIDE-5'-MONOPHOSPHATE |
| MHO | 20 | 52 | C5 H11 N O3 S | 165.21 | S-OXYMETHIONINE |
| IHP | 43 | 52 | C6 H18 O24 P6 | 660.04 | INOSITOL HEXAKISPHOSPHATE |
| EPH | 49 | 52 | C39 H68 N O8 P | 709.94 | L-ALPHA-PHOSPHATIDYL-BETA-OLEOYL-GAMMA-PALMITOYL-PHOSPHATIDYLETHANOLAMINE |
| CMH | 16 | 52 | C4 H9 HG N O2 S | 335.77 | S-(METHYLMERCURY)-L-CYSTEINE |
| C2F | 40 | 52 | C20 H25 N7 O6 | 459.46 | 5-METHYL-5,6,7,8-TETRAHYDROFOLIC ACID |
| AMG | 38 | 52 | C7 H14 O6 | 194.18 | ALPHA-METHYL-D-GALACTOSIDE |
| A5P | 32 | 52 | C5 H13 O8 P | 232.13 | ARABINOSE-5-PHOSPHATE |
| URF | 35 | 51 | C4 H3 F N2 O2 | 130.08 | 5-FLUOROURACIL |
| TMO | 17 | 51 | C3 H9 N O | 75.11 | TRIMETHYLAMINE OXIDE |
| TCA | 15 | 51 | C9 H8 O2 | 148.16 | PHENYLETHYLENECARBOXYLIC ACID |
| NEP | 46 | 51 | C6 H10 N3 O5 P | 235.14 | N1-PHOSPHONOHISTIDINE |
| RUB | 34 | 50 | C5 H12 O11 P2 | 310.09 | RIBULOSE-1,5-DIPHOSPHATE |
| NVP | 50 | 50 | C15 H14 N4 O | 266.3 | 11-CYCLOPROPYL-5,11-DIHYDRO-4-METHYL-6H-DIPYRIDO[3,2-B:2',3'-E][1,4]DIAZEPIN-6-ONE |
| NCT | 30 | 50 | C10 H14 N2 | 162.23 | (S)-3-(1-METHYLPYRROLIDIN-2-YL)PYRIDINE |
| GCO | 30 | 50 | C6 H12 O7 | 196.16 | GLUCONIC ACID |
| G39 | 48 | 50 | C14 H24 N2 O4 | 284.36 | (3R,4R,5S)-4-(ACETYLAMINO)-5-AMINO-3-(PENTAN-3-YLOXY)CYCLOHEX-1-ENE-1-CARBOXYLIC ACID |
| ETX | 33 | 50 | C4 H10 O2 | 90.12 | 2-ETHOXYETHANOL |
| DY | 8 | 50 | DY 2 | 162.5 | DYSPROSIUM ION |
| CBS | 29 | 50 | C16 H28 N2 O11 | 424.4 | DI(N-ACETYL-D-GLUCOSAMINE) |
| C2O | 41 | 50 | CU2 O | 143.09 | CU-O-CU LINKAGE |
| ACN | 34 | 50 | C3 H6 O | 58.08 | ACETONE |
| ACM | 33 | 50 | C2 H5 N O | 59.07 | ACETAMIDE |
| 6MZ | 38 | 50 | C11 H16 N5 O7 P | 361.25 | N6-METHYLADENOSINE-5'-MONOPHOSPHATE |
| TBG | 38 | 49 | C6 H13 N O2 | 131.17 | 3-METHYL-L-VALINE |
| TB | 23 | 49 | TB 3 | 158.93 | TERBIUM(III) ION |
| SPO | 49 | 49 | C41 H60 O | 568.92 | SPHEROIDENE |
| QUE | 39 | 49 | C15 H10 O7 | 302.24 | 3,5,7,3',4'-PENTAHYDROXYFLAVONE |
| PGR | 30 | 49 | C3 H8 O2 | 76.1 | R-1,2-PROPANEDIOL |
| L2P | 27 | 49 | C43 H88 O3 | 653.17 | 2,3-DI-PHYTANYL-GLYCEROL |
| IIL | 12 | 49 | C6 H13 N O2 | 131.17 | ISO-ISOLEUCINE |
| IBM | 44 | 49 | C10 H14 N4 O2 | 222.25 | 3-ISOBUTYL-1-METHYLXANTHINE |
| Y5P | 9 | 48 | C9 H15 N2 O7 P | 294.2 | 1-(5-O-PHOSPHONO-BETA-D-RIBOFURANOSYL)-1,4-DIHYDROPYRIMIDINE |
| T44 | 36 | 48 | C15 H11 I4 N O4 | 776.87 | 3,5,3',5'-TETRAIODO-L-THYRONINE |
| SPH | 48 | 48 | C18 H37 N O2 | 299.5 | SPHINGOSINE |
| S6P | 44 | 48 | C6 H15 O9 P | 262.15 | D-SORBITOL-6-PHOSPHATE |
| RJP | 24 | 48 | C16 H17 N O6 | 319.31 | (1R,5R)-1,5-DIHYDROXY-4-OXO-3-[3-OXO-3-(PHENYLAMINO)PROPYL]CYCLOHEX-2-ENE-1-CARBOXYLIC ACID |
| PBZ | 27 | 48 | C7 H10 N3 1 | 136.18 | P-AMINO BENZAMIDINE |
| OMT | 30 | 48 | C5 H11 N O4 S | 181.21 | S-DIOXYMETHIONINE |
| NIO | 35 | 48 | C6 H5 N O2 | 123.11 | NICOTINIC ACID |
| N87 | 24 | 48 | C20 H18 O6 | 354.36 | (1R,4R,5R)-1,4,5-TRIHYDROXY-3-[3-(PHENYLCARBONYL)PHENYL]CYCLOHEX-2-ENE-1-CARBOXYLIC ACID |
| MHS | 26 | 48 | C7 H11 N3 O2 | 169.18 | N1-METHYLATED HISTIDINE |
| M2G | 45 | 48 | C12 H18 N5 O8 P | 391.28 | N2-DIMETHYLGUANOSINE-5'-MONOPHOSPHATE |
| FPS | 34 | 48 | C15 H28 O6 P2 S | 398.39 | S-[(2E,6E)-3,7,11-TRIMETHYLDODECA-2,6,10-TRIENYL] TRIHYDROGEN THIODIPHOSPHATE |
| FEC | 48 | 48 | C36 H36 FE N4 O8 2 | 708.55 | 1,3,5,8-TETRAMETHYL-PORPHINE-2,4,6,7-TETRAPROPIONIC ACID FERROUS COMPLEX |
| D12 | 22 | 48 | C12 H26 | 170.34 | DODECANE |
| A23 | 36 | 48 | C10 H13 N5 O9 P2 | 409.19 | ADENOSINE-5'-PHOSPHATE-2',3'-CYCLIC PHOSPHATE |
| 6PG | 30 | 48 | C6 H13 O10 P | 276.14 | 6-PHOSPHOGLUCONIC ACID |
| PCG | 29 | 47 | C10 H12 N5 O7 P | 345.21 | CYCLIC GUANOSINE MONOPHOSPHATE |
| PAR | 43 | 47 | C23 H45 N5 O14 | 615.63 | PAROMOMYCIN |
| INS | 42 | 47 | C6 H12 O6 | 180.16 | 1,2,3,4,5,6-HEXAHYDROXY-CYCLOHEXANE |
| FTT | 12 | 47 | C14 H28 O3 | 244.37 | 3-HYDROXY-TETRADECANOIC ACID |
| FMC | 33 | 47 | C10 H13 N5 O4 | 267.24 | (1S)-1-(7-AMINO-1H-PYRAZOLO[4,3-D]PYRIMIDIN-3-YL)-1,4-ANHYDRO-D-RIBITOL |
| CPT | 32 | 47 | CL2 H6 N2 PT | 300.06 | CISPLATIN |
| XMP | 34 | 46 | C10 H14 N4 O9 P 1 | 365.22 | XANTHOSINE-5'-MONOPHOSPHATE |
| OAS | 45 | 46 | C5 H9 N O4 | 147.13 | O-ACETYLSERINE |
| GL3 | 46 | 46 | C2 H5 N O S | 91.13 | THIOGLYCIN |
| CIR | 36 | 46 | C6 H13 N3 O3 | 175.19 | CITRULLINE |
| BEM | 25 | 46 | C6 H10 O7 | 194.14 | BETA-D-MANNURONIC ACID |
| Y01 | 19 | 45 | C31 H50 O4 | 486.73 | CHOLESTEROL HEMISUCCINATE |
| UGA | 26 | 45 | C15 H22 N2 O18 P2 | 580.29 | URIDINE-5'-DIPHOSPHATE-GLUCURONIC ACID |
| THJ | 24 | 45 | O3 S2 -2 | 112.12 | THIOSULFATE |
| S9L | 45 | 45 | C6 H15 O7 P | 230.15 | 2-[2-(2-HYDROXYETHOXY)ETHOXY]ETHYL DIHYDROGEN PHOSPHATE |
| RU | 21 | 45 | RU 3 | 101.07 | RUTHENIUM ION |
| PQQ | 45 | 45 | C14 H6 N2 O8 | 330.21 | PYRROLOQUINOLINE QUINONE |
| NMY | 31 | 45 | C23 H46 N6 O13 | 614.65 | NEOMYCIN |
| HMR | 29 | 45 | C7 H16 N4 O2 | 188.23 | BETA-HOMOARGININE |
| EST | 43 | 45 | C18 H24 O2 | 272.39 | ESTRADIOL |
| CY3 | 38 | 45 | C3 H8 N2 O S | 120.17 | 2-AMINO-3-MERCAPTO-PROPIONAMIDE |
| BB2 | 39 | 45 | C19 H35 N3 O5 | 385.5 | ACTINONIN |
| ALQ | 41 | 45 | C4 H8 O2 | 88.11 | 2-METHYL-PROPIONIC ACID |
| 3FG | 18 | 45 | C8 H9 N O4 | 183.16 | (2S)-AMINO(3,5-DIHYDROXYPHENYL)ETHANOIC ACID |
| UQ2 | 43 | 44 | C19 H26 O4 | 318.41 | UBIQUINONE-2 |
| TYI | 26 | 44 | C9 H9 I2 N O3 | 432.98 | 3,5-DIIODOTYROSINE |
| TBR | 27 | 44 | BR12 TA6 | 2044.54 | HEXATANTALUM DODECABROMIDE |
| SSU | 8 | 44 | C9 H13 N2 O8 P S | 340.24 | URIDINE-5'-PHOSPHOROTHIOATE |
| SC2 | 16 | 44 | C5 H9 N O3 S | 163.19 | N-ACETYL-L-CYSTEINE |
| PPI | 28 | 44 | C3 H6 O2 | 74.08 | PROPANOIC ACID |
| PAL | 29 | 44 | C6 H10 N O8 P | 255.12 | N-(PHOSPHONACETYL)-L-ASPARTIC ACID |
| P77 | 10 | 44 | C20 H24 CL N3 S | 373.94 | 2-CHLORO-10-[3-(4-METHYLPIPERAZIN-1-YL)PROPYL]-10H-PHENOTHIAZINE |
| MBO | 36 | 44 | C7 H5 HG O2 | 321.7 | MERCURIBENZOIC ACID |
| LI | 39 | 44 | LI 1 | 6.94 | LITHIUM ION |
| KIW | 24 | 44 | C15 H12 N2 O6 | 316.27 | 5-[(3-NITROBENZYL)AMINO]BENZENE-1,3-DICARBOXYLIC ACID |
| KIU | 24 | 44 | C15 H11 N O7 | 317.25 | 5-[(3-NITROBENZYL)OXY]BENZENE-1,3-DICARBOXYLIC ACID |
| ILE | 33 | 44 | C6 H13 N O2 | 131.17 | ISOLEUCINE |
| DBB | 33 | 44 | C4 H9 N O2 | 103.12 | D-ALPHA-AMINOBUTYRIC ACID |
| CAQ | 32 | 44 | C6 H6 O2 | 110.11 | CATECHOL |
| BMQ | 22 | 44 | C9 H13 N2 O10 P | 340.18 | 1-(5'-PHOSPHO-BETA-D-RIBOFURANOSYL)BARBITURIC ACID |
| 1R2 | 24 | 44 | C13 H9 N O6 | 275.22 | 3-HYDROXY-5-(3-NITROPHENOXY)BENZOIC ACID |
| UPL | 2 | 43 | C34 H70 | 478.93 | UNKNOWN BRANCHED FRAGMENT OF PHOSPHOLIPID |
| STE | 25 | 43 | C18 H36 O2 | 284.48 | STEARIC ACID |
| PE3 | 27 | 43 | C28 H58 O15 | 634.76 | 3,6,9,12,15,18,21,24,27,30,33,36,39-TRIDECAOXAHENTETRACONTANE-1,41-DIOL |
| MO | 43 | 43 | MO | 95.94 | MOLYBDENUM ATOM |
| MK8 | 28 | 43 | C7 H15 N O2 | 145.2 | 2-METHYL-L-NORLEUCINE |
| DCS | 25 | 43 | C11 H16 N3 O7 P | 333.24 | D-[3-HYDROXY-2-METHYL-5-PHOSPHONOOXYMETHYL-PYRIDIN-4-YLMETHYL]-N,O-CYCLOSERYLAMIDE |
| CTT | 34 | 43 | C24 H42 O21 | 666.58 | BETA-D-GLUCOPYRANOSYL-(1->4)-BETA-D-GLUCOPYRANOSYL-(1->4)-BETA-D-GLUCOPYRANOSYL-(1->4)-BETA-D-GLUCOPYRANOSE |
| CAF | 21 | 43 | C5 H12 AS N O3 S | 241.14 | S-DIMETHYLARSINOYL-CYSTEINE |
| YCP | 42 | 42 | C6 H11 N O2 | 129.16 | (2S)-PIPERIDINE-2-CARBOXYLIC ACID |
| SME | 30 | 42 | C5 H11 N O3 S | 165.21 | METHIONINE SULFOXIDE |
| M1N | 14 | 42 | C23 H32 B N3 O5 | 441.33 | (1R)-3-METHYL-1-{[N-(MORPHOLIN-4-YLCARBONYL)-3-(1-NAPHTHYL)-D-ALANYL]AMINO}BUTYLBORONIC ACID |
| KAI | 42 | 42 | C10 H15 N O4 | 213.23 | 3-(CARBOXYMETHYL)-4-ISOPROPENYLPROLINE |
| HNI | 40 | 42 | C34 H32 N4 NI O4 | 619.35 | PROTOPORPHYRIN IX CONTAINING NI(II) |
| FUA | 28 | 42 | C31 H48 O6 | 516.72 | FUSIDIC ACID |
| DPO | 34 | 42 | O7 P2 -4 | 173.94 | DIPHOSPHATE |
| DDQ | 17 | 42 | C12 H27 N O | 201.35 | DECYLAMINE-N,N-DIMETHYL-N-OXIDE |
| AMU | 33 | 42 | C11 H19 N O8 | 293.27 | BETA-N-ACETYLMURAMIC ACID |
| 3PO | 20 | 42 | H5 O10 P3 | 257.95 | TRIPHOSPHATE |
| 39Y | 28 | 42 | C8 H14 O2 | 142.2 | (2E,5S)-5-methylhept-2-enoic acid |
| XLS | 23 | 41 | C5 H10 O5 | 150.13 | D-XYLOSE (LINEAR FORM) |
| SGC | 21 | 41 | C6 H12 O5 S | 196.22 | 4-DEOXY-4-THIO-BETA-D-GLUCOPYRANOSE |
| SCH | 28 | 41 | C4 H9 N O2 S2 | 167.24 | S-METHYL-THIO-CYSTEINE |
| MN3 | 32 | 41 | MN 3 | 54.94 | MANGANESE (III) ION |
| LVS | 28 | 41 | C28 H43 I N4 O8 S | 722.63 | 4-IODO-3-NITROPHENYL ACETYL-LEUCINYL-LEUCINYL-LEUCINYL-VINYLSULFONE |
| GXL | 17 | 41 | C6 H12 O6 | 180.16 | ALPHA-L-GALACTOPYRANOSE |
| DMN | 8 | 41 | C2 H7 N | 45.08 | DIMETHYLAMINE |
| DHT | 41 | 41 | C19 H30 O2 | 290.45 | 5-ALPHA-DIHYDROTESTOSTERONE |
| BLM | 20 | 41 | C55 H85 N17 O21 S3 | 1416.56 | BLEOMYCIN A2 |
| XRX | 20 | 40 | C9 H20 N2 O2 | 188.27 | 3-(DIMETHYLAMINO)BUTYL DIMETHYLCARBAMATE |
| THR | 30 | 40 | C4 H9 N O3 | 119.12 | THREONINE |
| NET | 40 | 40 | C8 H20 N 1 | 130.25 | TETRAETHYLAMMONIUM ION |
| N8E | 27 | 40 | C18 H38 O6 | 350.49 | 3,6,9,12,15-PENTAOXATRICOSAN-1-OL |
| MTN | 17 | 40 | C10 H18 N O3 S2 | 264.38 | S-[(1-OXYL-2,2,5,5-TETRAMETHYL-2,5-DIHYDRO-1H-PYRROL-3-YL)METHYL] METHANESULFONOTHIOATE |
| LMD | 10 | 40 | C26 H50 O11 | 538.67 | TETRADECYL 4-O-ALPHA-D-GLUCOPYRANOSYL-BETA-D-GLUCOPYRANOSIDE |
| IMH | 27 | 40 | C11 H14 N4 O4 | 266.26 | 1,4-DIDEOXY-4-AZA-1-(S)-(9-DEAZAHYPOXANTHIN-9-YL)-D-RIBITOL |
| FEB | 24 | 40 | C27 H51 N5 O7 | 557.73 | N~2~-[(3R)-3-HYDROXYDODECANOYL]-L-ASPARAGINYL-N~1~-[(1S)-1-(HYDROXYMETHYL)-3-METHYLBUTYL]-L-GLUTAMAMIDE |
| DPP | 38 | 40 | C3 H8 N2 O2 | 104.11 | DIAMINOPROPANOIC ACID |
| DCL | 36 | 40 | C6 H15 N O | 117.19 | 2-AMINO-4-METHYL-PENTAN-1-OL |
| AHG | 20 | 40 | C6 H14 O11 P2 | 324.12 | 2,5-ANHYDROGLUCITOL-1,6-BIPHOSPHATE |
| ACA | 33 | 40 | C6 H13 N O2 | 131.17 | 6-AMINOHEXANOIC ACID |
| 4LJ | 10 | 40 | C3 H8 Br N | 138.01 | 1.7.6 3-bromanylpropan-1-amine |
| 4LE | 20 | 40 | C3 H2 Cl F5 O | 184.49 | (2R)-2-chloro-2-(difluoromethoxy)-1,1,1-trifluoroethane |
| 1PS | 21 | 40 | C8 H11 N O3 S | 201.24 | 3-PYRIDINIUM-1-YLPROPANE-1-SULFONATE |
| TAC | 23 | 39 | C22 H24 N2 O8 | 444.44 | TETRACYCLINE |
| PFF | 34 | 39 | C9 H10 F N O2 | 183.18 | 4-FLUORO-L-PHENYLALANINE |
| NPO | 30 | 39 | C6 H5 N O3 | 139.11 | P-NITROPHENOL |
| NGT | 28 | 39 | C8 H13 N O4 S | 219.26 | 3AR,5R,6S,7R,7AR-5-HYDROXYMETHYL-2-METHYL-5,6,7,7A-TETRAHYDRO-3AH-PYRANO[3,2-D]THIAZOLE-6,7-DIOL |
| MYA | 39 | 39 | C35 H62 N7 O17 P3 S | 977.89 | TETRADECANOYL-COA |
| GTS | 22 | 39 | C10 H17 N3 O9 S | 355.32 | GLUTATHIONE SULFONIC ACID |
| GTB | 23 | 39 | C17 H22 N4 O8 S | 442.44 | S-(P-NITROBENZYL)GLUTATHIONE |
| GMP | 27 | 39 | C10 H13 N5 O5 | 283.24 | GUANOSINE |
| GLV | 32 | 39 | C2 H2 O3 | 74.04 | GLYOXYLIC ACID |
| BGM | 15 | 39 | C10 H13 BR N5 O7 P | 426.12 | 8-BROMO-2'-DEOXYGUANOSINE-5'-MONOPHOSPHATE |
| BDP | 25 | 39 | C6 H10 O7 | 194.14 | BETA-D-GLUCOPYRANURONIC ACID |
| 22B | 30 | 39 | C50 H76 O4 | 741.15 | BACTERIORUBERIN |
| OEC | 38 | 38 | CA MN4 O4 | 323.83 | OXYGEN EVOLVING SYSTEM |
| KDA | 38 | 38 | C11 H18 O8 | 278.26 | (3-DEOXY-D-MANNO-OCT-2-ULOSONIC ACID)-2-O-ALLYL |
| IYR | 25 | 38 | C9 H10 I N O3 | 307.09 | 3-IODO-TYROSINE |
| HXC | 25 | 38 | C27 H46 N7 O17 P3 S | 865.68 | HEXANOYL-COENZYME A |
| GOA | 37 | 38 | C2 H4 O3 | 76.05 | GLYCOLIC ACID |
| GMH | 25 | 38 | C7 H14 O7 | 210.18 | L-GLYCERO-D-MANNO-HEPTOPYRANOSE |
| FU2 | 26 | 38 | C5 H4 O2 | 96.08 | FURFURAL |
| DND | 30 | 38 | C21 H27 N6 O15 P2 1 | 665.42 | NICOTINIC ACID ADENINE DINUCLEOTIDE |
| DDG | 38 | 38 | C10 H14 N5 O6 P | 331.22 | 2',3'-DIDEOXY-GUANOSINE-5'-MONOPHOSPHATE |
| CO2 | 29 | 38 | C O2 | 44.01 | CARBON DIOXIDE |
| ANU | 24 | 38 | C9 H10 N2 O5 | 226.19 | 2,2'-ANHYDROURIDINE |
| 3PH | 29 | 38 | C39 H77 O8 P | 705.01 | 1,2-DIACYL-GLYCEROL-3-SN-PHOSPHATE |
| TOP | 31 | 37 | C14 H18 N4 O3 | 290.32 | TRIMETHOPRIM |
| PAU | 23 | 37 | C9 H17 N O5 | 219.24 | PANTOTHENOIC ACID |
| NMG | 37 | 37 | C3 H7 N3 O2 | 117.11 | GUANIDINO ACETATE |
| NME | 37 | 37 | C H5 N | 31.06 | METHYLAMINE |
| NHW | 37 | 37 | C36 H64 N7 O17 P3 S | 991.92 | 2-OXOPENTADECYL-COA |
| MLC | 15 | 37 | C24 H38 N7 O19 P3 S | 853.58 | MALONYL-COENZYME A |
| HSO | 21 | 37 | C6 H12 N3 O 1 | 140.16 | L-HISTIDINOL |
| FON | 23 | 37 | C20 H23 N7 O7 | 473.44 | N-{[4-({[(6R)-2-AMINO-5-FORMYL-4-OXO-1,4,5,6,7,8-HEXAHYDROPTERIDIN-6-YL]METHYL}AMINO)PHENYL]CARBONYL}-L-GLUTAMIC ACID |
| C | 22 | 37 | C9 H14 N3 O8 P | 323.2 | CYTIDINE-5'-MONOPHOSPHATE |
| ACR | 27 | 37 | C25 H43 N O18 | 645.61 | ALPHA-ACARBOSE |
| 4CO | 19 | 37 | C29 H42 N7 O18 P3 S | 901.67 | 4-HYDROXYPHENACYL COENZYME A |
| 0FQ | 13 | 37 | C29 H42 N7 O17 P3 S | 885.67 | PHENACYL COENZYME A |
| XCC | 18 | 36 | FE4 NI S4 | 410.33 | FE(4)-NI(1)-S(4) CLUSTER |
| VIB | 20 | 36 | C12 H17 N4 O S 1 | 265.35 | 3-(4-AMINO-2-METHYL-PYRIMIDIN-5-YLMETHYL)-5-(2-HYDROXY-ETHYL)-4-METHYL-THIAZOL-3-IUM |
| TSA | 22 | 36 | C10 H12 O6 | 228.2 | 8-HYDROXY-2-OXA-BICYCLO[3.3.1]NON-6-ENE-3,5-DICARBOXYLIC ACID |
| SOR | 22 | 36 | C6 H14 O6 | 182.17 | D-SORBITOL |
| PPY | 24 | 36 | C9 H8 O3 | 164.16 | 3-PHENYLPYRUVIC ACID |
| PAM | 31 | 36 | C16 H30 O2 | 254.41 | PALMITOLEIC ACID |
| OEX | 36 | 36 | CA MN4 O5 | 339.83 | CA-MN4-O5 CLUSTER |
| NIT | 30 | 36 | C6 H6 N2 O2 | 138.13 | 4-NITROANILINE |
| NAA | 20 | 36 | C8 H15 N O6 | 221.21 | N-ACETYL-D-ALLOSAMINE |
| MBN | 24 | 36 | C7 H8 | 92.14 | TOLUENE |
| IM5 | 18 | 36 | C12 H17 N5 O3 | 279.3 | 2-AMINO-7-{[(3R,4R)-3-HYDROXY-4-(HYDROXYMETHYL)PYRROLIDIN-1-YL]METHYL}-3,5-DIHYDRO-4H-PYRROLO[3,2-D]PYRIMIDIN-4-ONE |
| HCS | 29 | 36 | C4 H9 N O2 S | 135.18 | 2-AMINO-4-MERCAPTO-BUTYRIC ACID |
| GDX | 24 | 36 | C16 H23 N5 O17 P2 | 619.33 | GUANOSINE 5'-(TRIHYDROGEN DIPHOSPHATE), P'-D-MANNOPYRANOSYL ESTER |
| DCT | 34 | 36 | C9 H16 N3 O12 P3 | 451.16 | 2',3'-DIDEOXYCYTIDINE 5'-TRIPHOSPHATE |
| ARM | 36 | 36 | C7 H16 N4 O | 172.23 | DEOXY-METHYL-ARGININE |
| AHZ | 20 | 36 | C17 H19 N6 O12 P2 S -3 | 593.38 | ADENOSINE DIPHOSPHATE 5-(BETA-ETHYL)-4-METHYL-THIAZOLE-2-CARBOXYLIC ACID |
| 16G | 26 | 36 | C8 H16 N O9 P | 301.19 | N-ACETYL-D-GLUCOSAMINE-6-PHOSPHATE |
| 00S | 36 | 36 | C8 H11 N3 | 149.2 | 4-(AMINOMETHYL)BENZENECARBOXIMIDAMIDE |
| UVW | 10 | 35 | C2 H5 O5 P | 140.03 | ACETYLPHOSPHATE |
| URC | 27 | 35 | C5 H4 N4 O3 | 168.11 | URIC ACID |
| UQ | 33 | 35 | C59 H90 O4 | 863.36 | COENZYME Q10, (2Z,6E,10Z,14E,18E,22E,26Z)-ISOMER |
| SKM | 35 | 35 | C7 H10 O5 | 174.15 | (3R,4S,5R)-3,4,5-TRIHYDROXYCYCLOHEX-1-ENE-1-CARBOXYLIC ACID |
| RMN | 19 | 35 | C8 H8 O3 | 152.15 | (R)-MANDELIC ACID |
| PHD | 35 | 35 | C4 H8 N O7 P | 213.08 | ASPARTYL PHOSPHATE |
| PC | 22 | 35 | C5 H15 N O4 P 1 | 184.15 | PHOSPHOCHOLINE |
| LAC | 34 | 35 | C3 H6 O3 | 90.08 | LACTIC ACID |
| IMN | 30 | 35 | C19 H16 CL N O4 | 357.79 | INDOMETHACIN |
| H4M | 18 | 35 | C31 H45 N6 O16 P | 788.7 | 5,10-DIMETHYLENE TETRAHYDROMETHANOPTERIN |
| CCC | 35 | 35 | C9 H13 N3 O10 P2 | 385.16 | CYTIDINE-5'-PHOSPHATE-2',3'-CYCLIC PHOSPHATE |
| A2M | 33 | 35 | C11 H16 N5 O7 P | 361.25 | 2'-O-METHYLADENOSINE 5'-(DIHYDROGEN PHOSPHATE) |
| 2PG | 33 | 35 | C3 H7 O7 P | 186.06 | 2-PHOSPHOGLYCERIC ACID |
| UQ1 | 29 | 34 | C14 H18 O4 | 250.29 | UBIQUINONE-1 |
| UOQ | 8 | 34 | C32 H56 N7 O17 P3 S | 935.81 | UNDECA-2-ONE COENZYME A |
| SNN | 28 | 34 | C4 H6 N2 O2 | 114.1 | L-3-AMINOSUCCINIMIDE |
| OMY | 28 | 34 | C9 H10 CL N O4 | 231.63 | (BETAR)-3-CHLORO-BETA-HYDROXY-L-TYROSINE |
| O4B | 15 | 34 | C12 H24 O6 | 264.32 | 1,4,7,10,13,16-HEXAOXACYCLOOCTADECANE |
| MRY | 25 | 34 | C4 H10 O4 | 122.12 | MESO-ERYTHRITOL |
| DHI | 32 | 34 | C6 H10 N3 O2 1 | 156.16 | D-HISTIDINE |
| CE1 | 19 | 34 | C28 H58 O9 | 538.76 | O-DODECANYL OCTAETHYLENE GLYCOL |
| BFD | 34 | 34 | C4 H6 BE F3 N O4 -2 | 198.1 | ASPARTATE BERYLLIUM TRIFLUORIDE |
| AS1 | 15 | 34 | C10 H18 N4 O6 | 290.27 | ARGININOSUCCINATE |
| AAL | 13 | 34 | C6 H10 O5 | 162.14 | 3,6-ANHYDRO-L-GALACTOSE |
| XY1 | 14 | 33 | C15 H15 N3 O3 | 285.3 | 4-[(E)-[5-(2-AZANYLETHYL)-2-OXIDANYL-PHENYL]DIAZENYL]BENZOIC ACID |
| TFA | 32 | 33 | C2 H F3 O2 | 114.02 | TRIFLUOROACETIC ACID |
| STR | 33 | 33 | C21 H30 O2 | 314.47 | PROGESTERONE |
| S4M | 33 | 33 | C14 H24 N6 O3 S | 356.44 | 5'-[(S)-(3-AMINOPROPYL)(METHYL)-LAMBDA~4~-SULFANYL]-5'-DEOXYADENOSINE |
| PHT | 19 | 33 | C8 H6 O4 | 166.13 | PHTHALIC ACID |
| PCP | 21 | 33 | C6 H15 O13 P3 | 388.1 | 1-ALPHA-PYROPHOSPHORYL-2-ALPHA,3-ALPHA-DIHYDROXY-4-BETA-CYCLOPENTANE-METHANOL-5-PHOSPHATE |
| PCI | 24 | 33 | C6 H CL5 O | 266.34 | PENTACHLOROPHENOL |
| NTM | 19 | 33 | C7 H5 N O4 | 167.12 | QUINOLINIC ACID |
| NLG | 33 | 33 | C7 H11 N O5 | 189.17 | N-ACETYL-L-GLUTAMATE |
| LBT | 28 | 33 | C12 H22 O11 | 342.3 | ALPHA-LACTOSE |
| G3P | 32 | 33 | C3 H9 O6 P | 172.07 | SN-GLYCEROL-3-PHOSPHATE |
| FFO | 17 | 33 | C20 H23 N7 O7 | 471.43 | N-[4-({[(6S)-2-AMINO-5-FORMYL-4-OXO-3,4,5,6,7,8-HEXAHYDROPTERIDIN-6-YL]METHYL}AMINO)BENZOYL]-L-GLUTAMIC ACID |
| FAH | 11 | 33 | C2 H3 F O2 | 78.04 | FLUOROACETIC ACID |
| CYT | 24 | 33 | C4 H5 N3 O | 111.1 | 6-AMINOPYRIMIDIN-2(1H)-ONE |
| BAU | 17 | 33 | C14 H16 N2 O4 | 276.29 | 1-((2-HYDROXYETHOXY)METHYL)-5-BENZYLPYRIMIDINE-2,4(1H,3H)-DIONE |
| A3S | 21 | 33 | C13 H19 N7 O5 | 353.34 | SERINE-3'-AMINOADENOSINE |
| 5PA | 33 | 33 | C12 H17 N2 O7 P | 332.25 | N-[3-HYDROXY-2-METHYL-5-PHOSPHONOOXYMETHYL-PYRIDIN-4-Y-LMETHYL]-1-AMINO-CYCLOPROPANECARBOXYLIC ACID |
| SN0 | 20 | 32 | C9 H15 N O5 | 217.22 | N-(3-CARBOXYPROPANOYL)-L-NORVALINE |
| RIP | 24 | 32 | C5 H10 O5 | 150.13 | RIBOSE(PYRANOSE FORM) |
| RIO | 16 | 32 | C17 H34 N4 O10 | 454.48 | RIBOSTAMYCIN |
| PSJ | 20 | 32 | C6 H12 O6 | 180.16 | D-PSICOSE |
| PIN | 20 | 32 | C8 H18 N2 O6 S2 | 302.36 | PIPERAZINE-N,N'-BIS(2-ETHANESULFONIC ACID) |
| OSF | 4 | 32 | C8 H17 O4 S -1 | 209.28 | OCTYL SULFATE |
| NFA | 31 | 32 | C9 H12 N2 O | 164.21 | PHENYLALANINE AMIDE |
| MH9 | 30 | 32 | C12 H20 O2 | 196.29 | (2E,4E)-DODECA-2,4-DIENOIC ACID |
| LWY | 32 | 32 | C6 H12 N O4 P | 193.14 | 1-[(R)-HYDROXY(METHYL)PHOSPHORYL]-L-PROLINE |
| G1P | 31 | 32 | C6 H13 O9 P | 260.14 | ALPHA-D-GLUCOSE-1-PHOSPHATE |
| FTR | 12 | 32 | C11 H11 F N2 O2 | 222.22 | FLUOROTRYPTOPHANE |
| DMT | 22 | 32 | C11 H21 N O3 | 215.29 | 3-HYDROXY-4,4-DIMETHYL-2-(METHYLAMINO)-6-OCTENOIC ACID |
| DFP | 32 | 32 | C6 H15 O3 P | 166.16 | DIISOPROPYL PHOSPHONATE |
| CMQ | 14 | 32 | C24 H32 N2 O5 | 428.53 | N~2~-[(BENZYLOXY)CARBONYL]-N-[(1S,2S)-2-HYDROXY-1-(4-HYDROXYBENZYL)PROPYL]-L-LEUCINAMIDE |
| BXB | 16 | 32 | C15 H11 CL2 N O3 | 324.16 | N-(1,3-BENZODIOXOL-5-YLMETHYL)-2,6-DICHLOROBENZAMIDE |
| BML | 16 | 32 | C6 H5 BR O | 173.01 | 4-BROMOPHENOL |
| BET | 20 | 32 | C5 H12 N O2 1 | 118.15 | TRIMETHYL GLYCINE |
| ASJ | 32 | 32 | C4 H9 N O3 | 119.12 | (3S)-3-AMINO-4-HYDROXYBUTANOIC ACID |
| AES | 16 | 32 | C8 H10 F N O2 S | 203.23 | 4-(2-AMINOETHYL)BENZENESULFONYL FLUORIDE |
| 0YR | 16 | 32 | C5 H11 N O6 | 181.15 | (2R,3R,4R)-N,2,3,4,5-PENTAKIS(OXIDANYL)PENTANAMIDE |
| 0VJ | 16 | 32 | C18 H14 O4 | 294.31 | 2-HYDROXY-3-(4-METHOXYBENZYL)NAPHTHALENE-1,4-DIONE |
| 09T | 16 | 32 | C16 H14 CL2 N2 | 305.21 | 1-(3,4-DICHLOROBENZYL)-5,6-DIMETHYL-1H-BENZIMIDAZOLE |
| XPC | 17 | 31 | C5 H10 N2 O2 | 130.15 | (3S,4R)-4-AMINOPYRROLIDINE-3-CARBOXYLIC ACID |
| PQ9 | 22 | 31 | C43 H64 O2 | 612.98 | 5-[(2E,6E,10E,14E,18E,22E)-3,7,11,15,19,23,27-HEPTAMETHYLOCTACOSA-2,6,10,14,18,22,26-HEPTAENYL]-2,3-DIMETHYLBENZO-1,4-QUINONE |
| PDC | 16 | 31 | C7 H5 N O4 | 167.12 | PYRIDINE-2,6-DICARBOXYLIC ACID |
| MIS | 29 | 31 | C6 H14 N O6 P | 227.15 | MONOISOPROPYLPHOSPHORYLSERINE |
| KR | 13 | 31 | KR | 83.8 | KRYPTON |
| GDN | 17 | 31 | C16 H19 N5 O10 S | 473.41 | GLUTATHIONE S-(2,4 DINITROBENZENE) |
| FCO | 31 | 31 | C3 FE N2 O | 135.89 | CARBONMONOXIDE-(DICYANO) IRON |
| CNC | 28 | 31 | C63 H88 CO N14 O14 P 1 | 1355.38 | CO-CYANOCOBALAMIN |

Table S3: Minimum, Maximum and Median Resolution of PDB chains for different ligands.

| Ligand ID | Resolution of PDB chains (Å) | | |
| --- | --- | --- | --- |
|  | Minimum | Maximum | Median |
| MSE | 1.00 | 20.00 | 1.78 |
| MG | 0.98 | 37.00 | 2.00 |
| SO4 | 0.84 | 9.00 | 1.56 |
| GOL | 0.85 | 4.19 | 1.50 |
| ZN | 0.97 | 36.00 | 2.25 |
| EDO | 0.85 | 3.88 | 1.50 |
| CA | 0.85 | 35.00 | 2.35 |
| NAG | 1.20 | 30.00 | 1.45 |
| CL | 0.97 | 13.00 | 2.00 |
| NA | 0.97 | 13.00 | 2.00 |
| PO4 | 1.00 | 19.00 | 3.50 |
| HEM | 1.04 | 19.00 | 1.50 |
| ACT | 1.00 | 3.50 | 2.54 |
| DMS | 1.12 | 3.20 | 1.65 |
| MN | 1.10 | 7.60 | 2.40 |
| K | 1.08 | 9.70 | 1.75 |
| IOD | 1.45 | 4.00 | 2.90 |
| FAD | 1.20 | 4.30 | 1.70 |
| CD | 1.02 | 4.20 | 1.65 |
| UNX | 1.00 | 11.40 | 2.50 |
| MAN | 1.06 | 20.50 | 2.38 |
| FE | 0.95 | 7.50 | 2.70 |
| ADP | 1.05 | 37.00 | 2.22 |
| CLA | 1.40 | 9.50 | 1.45 |
| NAD | 1.15 | 6.93 | 2.05 |
| PEG | 0.85 | 3.25 | 3.20 |
| HYP | 1.30 | 5.16 | 2.75 |
| MPD | 1.10 | 3.12 | 1.69 |
| CU | 1.02 | 19.00 | 1.50 |
| FMT | 1.30 | 7.50 | 1.50 |
| MLY | 1.40 | 70.00 | 2.50 |
| ACE | 0.90 | 10.10 | 1.65 |
| FE2 | 1.03 | 8.20 | 2.70 |
| PLP | 1.15 | 3.63 | 1.70 |
| ATP | 1.17 | 37.00 | 1.90 |
| GLC | 1.18 | 4.00 | 2.00 |
| ACY | 0.89 | 3.59 | 1.50 |
| BR | 1.15 | 3.70 | 2.75 |
| NAP | 1.08 | 6.93 | 2.36 |
| BME | 1.10 | 3.10 | 1.80 |
| FMN | 1.20 | 19.00 | 1.65 |
| NH2 | 1.05 | 10.10 | 1.85 |
| NI | 1.30 | 3.70 | 2.00 |
| SR | 1.19 | 4.67 | 2.00 |
| PG4 | 1.30 | 3.25 | 1.75 |
| SF4 | 1.20 | 19.00 | 1.74 |
| GAL | 1.25 | 5.00 | 2.12 |
| BCR | 1.90 | 8.98 | 4.00 |
| MES | 0.85 | 3.60 | 1.85 |
| TRS | 1.12 | 3.61 | 1.80 |
| HG | 1.00 | 3.80 | 3.00 |
| HEC | 0.84 | 19.00 | 1.74 |
| BGC | 1.20 | 3.65 | 1.90 |
| BMA | 1.06 | 20.50 | 1.90 |
| SEP | 1.16 | 6.60 | 2.80 |
| LDA | 1.72 | 3.60 | 3.30 |
| GDP | 1.00 | 28.00 | 2.30 |
| CO | 1.20 | 3.70 | 2.15 |
| BCL | 1.75 | 4.60 | 2.70 |
| NO3 | 0.98 | 3.25 | 2.30 |
| COA | 1.35 | 3.50 | 1.60 |
| TPO | 1.35 | 6.60 | 2.25 |
| FUC | 1.20 | 26.00 | 1.40 |
| BOG | 1.40 | 3.51 | 2.20 |
| EPE | 1.25 | 15.00 | 2.40 |
| NDP | 1.23 | 3.62 | 2.00 |
| IPA | 1.05 | 3.10 | 1.90 |
| PTR | 1.35 | 3.90 | 2.60 |
| CIT | 1.20 | 3.62 | 3.30 |
| NDG | 1.30 | 30.00 | 1.80 |
| LMT | 1.78 | 6.56 | 2.00 |
| FES | 1.18 | 19.00 | 2.20 |
| ANP | 1.25 | 28.00 | 9.40 |
| GTP | 1.20 | 28.00 | 2.70 |
| SAH | 1.20 | 3.82 | 2.00 |
| AMP | 1.33 | 4.30 | 2.70 |
| LMG | 1.90 | 8.98 | 6.56 |
| IMD | 1.17 | 3.05 | 1.63 |
| PGE | 0.85 | 3.20 | 1.80 |
| 1PE | 1.25 | 3.50 | 1.45 |
| MLE | 1.20 | 3.10 | 2.10 |
| LLP | 1.33 | 7.50 | 1.80 |
| CSO | 1.30 | 3.00 | 1.87 |
| MRD | 1.05 | 3.40 | 1.77 |
| DMF | 1.45 | 2.80 | 2.20 |
| DGD | 1.75 | 6.56 | 1.90 |
| KCX | 1.30 | 3.30 | 2.30 |
| CME | 1.45 | 2.95 | 2.35 |
| TRP | 1.30 | 3.30 | 1.65 |
| PCA | 0.84 | 13.70 | 1.95 |
| EOH | 0.83 | 3.30 | 1.80 |
| CYC | 1.35 | 3.50 | 3.00 |
| SQD | 1.90 | 6.56 | 3.07 |
| FLC | 1.39 | 4.30 | 2.80 |
| CDL | 1.60 | 19.00 | 2.20 |
| SCN | 1.27 | 3.30 | 1.70 |
| XYP | 1.14 | 3.20 | 2.22 |
| LHG | 1.90 | 8.98 | 1.90 |
| SIA | 1.30 | 4.00 | 1.90 |
| SMC | 1.16 | 2.80 | 2.20 |
| DAL | 1.20 | 3.80 | 1.73 |
| NCO | 1.26 | 3.35 | 2.85 |
| PEE | 1.90 | 4.09 | 3.16 |
| CSD | 1.28 | 3.10 | 1.60 |
| CGU | 1.55 | 3.38 | 2.70 |
| PYR | 1.40 | 3.20 | 2.00 |
| UMP | 1.47 | 3.30 | 1.98 |
| GAI | 1.42 | 2.70 | 1.85 |
| SAM | 1.35 | 18.00 | 1.55 |
| UDP | 1.32 | 3.51 | 2.80 |
| PX4 | 1.90 | 3.40 | 3.20 |
| GLA | 1.49 | 12.50 | 2.00 |
| CO3 | 1.35 | 7.50 | 2.80 |
| C8E | 1.59 | 3.50 | 1.94 |
| OLC | 1.80 | 3.19 | 2.00 |
| CAC | 1.05 | 4.20 | 1.95 |
| DIO | 1.33 | 3.10 | 1.85 |
| CMO | 1.03 | 3.50 | 1.95 |
| URE | 1.36 | 3.09 | 1.70 |
| FME | 1.20 | 8.20 | 2.60 |
| DLE | 1.25 | 2.50 | 1.93 |
| GLU | 1.24 | 12.80 | 1.85 |
| TLA | 1.20 | 3.40 | 2.00 |
| XE | 1.45 | 3.37 | 2.50 |
| TPP | 1.42 | 3.20 | 1.82 |
| IPH | 1.50 | 3.10 | 2.90 |
| OXY | 1.34 | 3.30 | 2.30 |
| ABA | 1.20 | 3.10 | 2.10 |
| ARG | 1.58 | 3.27 | 2.34 |
| POP | 1.40 | 3.60 | 2.10 |
| GNP | 1.26 | 23.00 | 1.90 |
| PSU | 1.57 | 17.00 | 3.00 |
| GSH | 1.30 | 3.38 | 1.90 |
| PLM | 1.35 | 9.00 | 1.40 |
| AZI | 1.20 | 3.51 | 2.40 |
| BA | 1.08 | 3.49 | 2.24 |
| BEN | 1.20 | 3.22 | 1.65 |
| TYS | 1.40 | 3.20 | 1.80 |
| CHD | 1.50 | 3.00 | 2.20 |
| BPH | 1.80 | 4.60 | 2.50 |
| MYR | 1.50 | 22.00 | 2.80 |
| CU1 | 1.30 | 3.25 | 3.25 |
| GLY | 1.35 | 3.96 | 1.84 |
| SUC | 1.50 | 3.49 | 1.98 |
| M3L | 1.10 | 2.97 | 2.32 |
| CS | 1.40 | 3.50 | 2.50 |
| U10 | 1.80 | 4.60 | 2.45 |
| H4B | 1.74 | 3.00 | 1.90 |
| CAP | 1.35 | 2.80 | 1.80 |
| SIN | 1.30 | 2.90 | 1.66 |
| DTT | 1.20 | 3.20 | 2.80 |
| ACO | 1.30 | 3.80 | 1.82 |
| PGO | 1.50 | 2.95 | 2.95 |
| MLI | 1.34 | 3.66 | 1.90 |
| P6G | 1.31 | 3.23 | 2.10 |
| LFA | 1.45 | 3.10 | 2.50 |
| PL9 | 1.90 | 6.56 | 5.90 |
| IUM | 1.20 | 3.40 | 1.25 |
| DMU | 1.70 | 3.27 | 2.30 |
| YCM | 1.40 | 3.40 | 1.60 |
| RCO | 1.36 | 2.50 | 1.36 |
| AKG | 1.31 | 3.20 | 1.69 |
| MME | 1.35 | 2.80 | 2.00 |
| CMP | 1.45 | 19.80 | 1.65 |
| TL | 1.80 | 8.40 | 2.60 |
| PGV | 1.80 | 3.91 | 2.10 |
| SAR | 1.20 | 3.10 | 2.40 |
| NAI | 1.50 | 19.00 | 1.85 |
| LI1 | 1.43 | 3.01 | 1.52 |
| BCT | 1.12 | 6.56 | 2.30 |
| ALY | 1.40 | 3.10 | 2.41 |
| CYN | 1.40 | 3.40 | 1.70 |
| CSX | 1.30 | 3.40 | 1.70 |
| OXL | 1.20 | 3.40 | 2.50 |
| NH4 | 1.45 | 3.40 | 2.46 |
| RET | 1.30 | 3.80 | 2.08 |
| BNG | 1.65 | 4.10 | 1.80 |
| F3S | 1.40 | 3.66 | 2.20 |
| FUL | 1.20 | 13.70 | 2.80 |
| OCS | 1.49 | 30.00 | 1.88 |
| DVA | 1.25 | 3.62 | 1.50 |
| U5P | 1.30 | 3.00 | 2.03 |
| BTN | 1.30 | 3.23 | 2.00 |
| MLA | 1.43 | 3.30 | 1.75 |
| DTP | 1.65 | 5.65 | 2.90 |
| HEZ | 0.81 | 3.41 | 1.75 |
| PHE | 1.25 | 8.90 | 2.80 |
| BEZ | 1.35 | 3.20 | 3.20 |
| A2G | 1.25 | 3.50 | 1.95 |
| NLE | 1.20 | 2.80 | 2.00 |
| LYS | 1.20 | 3.22 | 1.80 |
| NO2 | 1.40 | 3.10 | 1.75 |
| MDO | 1.50 | 2.56 | 2.40 |
| PT | 1.20 | 3.11 | 2.30 |
| TAR | 1.40 | 3.25 | 1.75 |
| DUR | 1.80 | 2.80 | 1.80 |
| BEF | 1.63 | 4.50 | 3.12 |
| 5GP | 1.29 | 3.45 | 2.90 |
| IDS | 1.42 | 3.40 | 2.00 |
| TYD | 1.40 | 3.15 | 2.60 |
| PEK | 1.80 | 2.70 | 2.10 |
| PMP | 1.50 | 3.00 | 2.20 |
| TGL | 1.80 | 2.70 | 1.90 |
| TTP | 1.50 | 3.30 | 2.20 |
| MOH | 1.25 | 2.50 | 2.35 |
| BTB | 1.30 | 3.04 | 2.50 |
| ADN | 1.30 | 3.30 | 1.90 |
| BMP | 1.26 | 3.00 | 1.60 |
| H2U | 1.93 | 17.00 | 3.00 |
| F6P | 1.50 | 3.40 | 2.40 |
| MMA | 1.30 | 3.00 | 2.31 |
| MAL | 1.48 | 3.98 | 2.50 |
| HIS | 1.47 | 2.80 | 1.80 |
| CAS | 1.70 | 3.00 | 1.70 |
| SO3 | 1.46 | 2.90 | 1.80 |
| NGA | 1.35 | 3.60 | 1.90 |
| XYS | 1.39 | 2.90 | 1.70 |
| B12 | 1.11 | 3.75 | 1.11 |
| APC | 1.56 | 3.29 | 1.80 |
| ALF | 1.50 | 7.70 | 1.90 |
| ASP | 1.60 | 3.51 | 2.80 |
| DGT | 1.78 | 3.99 | 2.39 |
| AU | 1.30 | 3.60 | 2.40 |
| DPN | 1.25 | 3.65 | 1.91 |
| TFP | 2.00 | 2.74 | 2.15 |
| PEP | 1.50 | 2.80 | 1.50 |
| HEA | 1.80 | 19.00 | 2.30 |
| HTG | 1.68 | 2.95 | 2.13 |
| HED | 1.25 | 2.79 | 1.65 |
| AIB | 0.90 | 3.10 | 1.80 |
| PHO | 1.90 | 6.56 | 2.10 |
| WO4 | 1.20 | 3.57 | 2.50 |
| FRU | 1.78 | 3.60 | 2.30 |
| 5MU | 1.93 | 17.00 | 2.70 |
| NHE | 1.30 | 3.40 | 2.40 |
| F09 | 1.60 | 2.75 | 2.50 |
| DGL | 1.25 | 3.80 | 1.65 |
| 5BU | 1.30 | 3.80 | 1.80 |
| ACP | 1.22 | 15.00 | 2.30 |
| TRD | 2.00 | 2.60 | 2.20 |
| HTO | 1.60 | 3.21 | 2.13 |
| CTP | 1.49 | 3.90 | 2.25 |
| C2E | 1.45 | 3.00 | 2.50 |
| MGD | 1.30 | 3.20 | 2.20 |
| BMT | 1.20 | 3.10 | 1.76 |
| 5MC | 1.93 | 17.00 | 3.00 |
| CHT | 1.50 | 2.80 | 2.25 |
| SRT | 1.60 | 2.75 | 2.70 |
| PB | 1.50 | 3.70 | 1.60 |
| CAA | 1.34 | 2.90 | 2.00 |
| NRQ | 1.45 | 2.70 | 1.50 |
| INI | 1.85 | 2.90 | 2.30 |
| GLN | 1.44 | 3.50 | 2.30 |
| C5P | 1.50 | 3.60 | 2.25 |
| UPG | 1.20 | 3.23 | 2.53 |
| KDO | 1.45 | 3.30 | 2.60 |
| B3L | 1.80 | 2.94 | 2.10 |
| AGS | 1.65 | 8.40 | 2.10 |
| UMQ | 1.90 | 3.55 | 3.41 |
| RAM | 1.32 | 2.90 | 1.91 |
| PCW | 1.90 | 2.20 | 2.10 |
| MPG | 1.60 | 3.10 | 2.57 |
| PEO | 1.50 | 2.90 | 1.75 |
| ORN | 1.43 | 3.03 | 2.90 |
| YB | 1.58 | 3.60 | 2.05 |
| APR | 1.47 | 11.70 | 1.90 |
| SGN | 1.42 | 3.20 | 3.00 |
| OH | 1.04 | 3.00 | 1.80 |
| 2CV | 2.30 | 3.20 | 3.00 |
| 2AN | 1.60 | 2.50 | 1.90 |
| PHQ | 1.20 | 3.15 | 1.87 |
| ADE | 1.45 | 3.20 | 2.70 |
| 2PE | 1.43 | 2.80 | 2.50 |
| CXS | 1.30 | 2.95 | 1.49 |
| NO | 1.30 | 2.85 | 2.00 |
| THM | 1.50 | 3.10 | 1.90 |
| MLZ | 1.45 | 2.80 | 1.95 |
| LEU | 1.30 | 4.50 | 3.50 |
| DOC | 1.58 | 4.60 | 1.70 |
| SRM | 1.25 | 2.90 | 2.04 |
| MET | 1.40 | 5.00 | 2.40 |
| URA | 1.30 | 3.20 | 3.20 |
| PGA | 1.30 | 3.00 | 2.03 |
| P3S | 2.00 | 3.50 | 3.50 |
| GTX | 1.70 | 2.75 | 2.30 |
| TDP | 1.39 | 2.80 | 2.30 |
| MVA | 1.20 | 3.20 | 2.60 |
| DPV | 2.42 | 2.80 | 2.80 |
| CHL | 1.95 | 9.50 | 2.50 |
| BRU | 1.40 | 6.60 | 2.30 |
| 8OG | 1.55 | 3.90 | 2.40 |
| STA | 1.30 | 3.30 | 2.70 |
| DPR | 1.25 | 2.70 | 2.06 |
| BLA | 1.20 | 3.20 | 2.10 |
| TPQ | 1.55 | 3.20 | 2.50 |
| CPS | 1.51 | 3.50 | 2.30 |
| BCB | 1.86 | 8.20 | 1.86 |
| 2HP | 1.27 | 2.80 | 2.00 |
| VO4 | 1.39 | 4.20 | 1.75 |
| 15P | 1.30 | 2.90 | 1.70 |
| CXE | 1.90 | 3.20 | 2.00 |
| CDP | 1.50 | 3.11 | 1.60 |
| GSP | 1.55 | 8.60 | 3.27 |
| ALA | 1.35 | 3.00 | 1.94 |
| CRO | 1.20 | 3.40 | 1.59 |
| BIL | 1.80 | 2.50 | 2.00 |
| PE4 | 1.40 | 3.20 | 2.40 |
| FBP | 1.60 | 3.20 | 2.75 |
| UD1 | 1.50 | 3.10 | 1.90 |
| OLA | 1.30 | 3.10 | 2.68 |
| IMP | 1.54 | 2.80 | 1.90 |
| DAO | 1.80 | 7.20 | 2.00 |
| PHB | 1.40 | 3.00 | 1.90 |
| LMU | 1.30 | 3.49 | 2.54 |
| MLT | 1.37 | 3.31 | 1.60 |
| ADA | 1.57 | 2.50 | 1.90 |
| A3P | 1.30 | 3.50 | 2.60 |
| CYS | 1.50 | 3.22 | 2.70 |
| AG | 1.70 | 4.37 | 3.30 |
| MAA | 1.40 | 3.80 | 2.10 |
| CSS | 1.36 | 2.90 | 2.80 |
| MP8 | 1.90 | 2.60 | 2.60 |
| HDD | 1.70 | 2.80 | 2.70 |
| 7MG | 1.93 | 17.00 | 3.00 |
| AF3 | 1.55 | 9.20 | 2.50 |
| SAC | 1.43 | 3.20 | 1.90 |
| HCA | 1.60 | 3.20 | 2.10 |
| 3PG | 1.47 | 3.20 | 1.56 |
| MGM | 2.40 | 2.90 | 2.70 |
| MEA | 1.70 | 12.50 | 2.30 |
| LAT | 1.19 | 2.95 | 2.25 |
| 13P | 1.50 | 3.00 | 2.10 |
| SER | 1.46 | 2.80 | 2.00 |
| RBF | 1.42 | 3.60 | 2.60 |
| FEO | 1.30 | 4.45 | 1.66 |
| YT3 | 1.60 | 2.90 | 1.80 |
| PLC | 2.00 | 3.53 | 2.90 |
| OCT | 1.50 | 3.10 | 2.20 |
| IPT | 1.55 | 3.20 | 1.90 |
| CRS | 1.50 | 2.50 | 1.78 |
| WFP | 2.60 | 3.20 | 2.60 |
| OTT | 1.90 | 2.00 | 1.90 |
| JHM | 2.80 | 3.40 | 3.40 |
| HIC | 1.35 | 8.90 | 2.00 |
| HC4 | 1.20 | 3.03 | 1.90 |
| AHR | 1.43 | 2.90 | 2.30 |
| URI | 1.40 | 3.10 | 2.44 |
| MTX | 1.70 | 3.45 | 2.20 |
| GUN | 1.60 | 3.40 | 3.00 |
| DCP | 1.53 | 3.44 | 2.00 |
| TAM | 1.45 | 3.50 | 2.70 |
| MIA | 2.60 | 10.00 | 8.70 |
| 3DR | 1.45 | 3.50 | 2.80 |
| RB | 1.70 | 4.20 | 1.82 |
| DUT | 1.80 | 3.60 | 2.00 |
| CLF | 1.60 | 3.20 | 1.60 |
| 1MA | 1.93 | 17.00 | 2.90 |
| U | 1.50 | 3.21 | 2.60 |
| TYR | 1.45 | 2.80 | 1.80 |
| PRP | 1.67 | 3.25 | 1.75 |
| MTE | 1.45 | 2.80 | 2.30 |
| CRQ | 1.59 | 2.90 | 1.90 |
| PE5 | 1.60 | 3.10 | 1.60 |
| SAL | 1.65 | 3.40 | 1.95 |
| IPE | 1.50 | 3.05 | 1.95 |
| 1PG | 1.30 | 3.20 | 1.59 |
| CXM | 1.40 | 3.00 | 2.60 |
| VAL | 1.20 | 3.00 | 1.65 |
| TRE | 1.50 | 3.40 | 2.05 |
| TMP | 1.50 | 3.00 | 1.90 |
| PC1 | 1.70 | 3.50 | 3.50 |
| IRI | 1.80 | 3.20 | 2.95 |
| F43 | 1.16 | 2.70 | 1.45 |
| UNK | 1.60 | 3.00 | 1.60 |
| SMA | 1.90 | 19.00 | 2.40 |
| P33 | 1.40 | 3.00 | 1.95 |
| OGA | 1.60 | 2.89 | 1.95 |
| DHB | 1.80 | 2.41 | 1.80 |
| CLR | 1.60 | 3.45 | 3.21 |
| B3P | 1.30 | 2.80 | 2.48 |
| ARF | 1.36 | 2.40 | 2.37 |
| MTA | 1.45 | 2.91 | 1.95 |
| MGE | 3.00 | 4.00 | 3.70 |
| DUP | 1.49 | 3.20 | 1.80 |
| CB3 | 1.90 | 3.25 | 2.50 |
| ALC | 1.80 | 2.70 | 2.44 |
| OMC | 1.93 | 17.00 | 2.50 |
| IT1 | 1.28 | 3.30 | 1.77 |
| BOC | 1.50 | 3.10 | 1.90 |
| 5IU | 1.60 | 3.59 | 2.50 |
| HEX | 1.50 | 2.25 | 2.10 |
| HBI | 1.40 | 3.20 | 1.71 |
| GHP | 0.89 | 2.80 | 2.05 |
| DSN | 1.25 | 3.62 | 2.65 |
| ARS | 1.60 | 2.90 | 1.60 |
| O | 1.30 | 3.50 | 2.55 |
| MEN | 1.35 | 3.50 | 1.85 |
| DCY | 1.25 | 2.70 | 2.10 |
| CBI | 1.26 | 3.30 | 2.11 |
| MPO | 1.05 | 2.80 | 2.79 |
| COM | 1.16 | 2.70 | 1.30 |
| UFP | 1.53 | 3.45 | 2.50 |
| GCP | 1.54 | 7.20 | 2.60 |
| DTR | 1.25 | 3.10 | 1.55 |
| ASA | 1.69 | 2.80 | 2.80 |
| TBU | 1.25 | 2.80 | 2.20 |
| LMZ | 2.05 | 3.30 | 2.05 |
| CR2 | 1.23 | 2.60 | 1.45 |
| BU3 | 1.42 | 2.30 | 1.42 |
| P4C | 1.55 | 2.80 | 2.25 |
| GDU | 1.40 | 3.15 | 2.40 |
| RG1 | 2.00 | 2.50 | 2.00 |
| DLY | 1.40 | 2.70 | 1.55 |
| XCP | 1.80 | 2.80 | 2.00 |
| UTP | 1.80 | 4.30 | 4.30 |
| SFG | 1.42 | 3.80 | 2.41 |
| PEB | 1.63 | 2.40 | 2.01 |
| BU1 | 1.40 | 2.80 | 2.15 |
| 08T | 3.21 | 3.60 | 3.21 |
| ZIL | 3.20 | 3.20 | 3.20 |
| UP6 | 1.30 | 1.90 | 1.65 |
| SM | 1.60 | 4.20 | 1.80 |
| OPC | 3.00 | 3.80 | 3.41 |
| OAA | 1.25 | 3.60 | 2.15 |
| F | 1.55 | 3.50 | 2.00 |
| 5AD | 1.11 | 2.90 | 2.80 |
| PLG | 1.90 | 2.90 | 2.50 |
| GPP | 1.55 | 3.11 | 2.50 |
| 0QE | 1.50 | 2.90 | 2.10 |
| PRO | 1.35 | 3.21 | 1.78 |
| OMG | 1.93 | 17.00 | 3.00 |
| CR8 | 1.40 | 2.90 | 2.00 |
| BHG | 1.32 | 2.50 | 2.50 |
| TCL | 1.75 | 2.60 | 2.44 |
| PEV | 2.50 | 2.75 | 2.75 |
| BPB | 1.80 | 8.20 | 2.40 |
| THP | 1.40 | 2.30 | 1.99 |
| G6P | 1.60 | 2.95 | 2.40 |
| ETA | 1.70 | 2.55 | 2.50 |
| 12P | 1.30 | 2.50 | 2.00 |
| MAE | 1.80 | 2.80 | 2.70 |
| B3K | 1.80 | 2.30 | 2.00 |
| 4SU | 2.40 | 10.00 | 2.80 |
| 2GP | 1.23 | 2.40 | 1.76 |
| TRQ | 1.40 | 2.60 | 1.85 |
| CM5 | 1.76 | 3.20 | 3.00 |
| AZA | 1.50 | 2.70 | 1.75 |
| GYC | 1.65 | 2.35 | 1.80 |
| B3E | 1.80 | 2.80 | 2.00 |
| 3CO | 1.60 | 2.95 | 1.78 |
| PGH | 1.45 | 2.80 | 2.50 |
| IVA | 1.30 | 3.30 | 2.40 |
| DKA | 1.36 | 2.70 | 1.80 |
| 1MG | 2.60 | 7.50 | 3.00 |
| PGW | 2.40 | 2.98 | 2.56 |
| PE8 | 1.60 | 2.82 | 1.76 |
| KPI | 1.30 | 2.40 | 2.30 |
| GRG | 1.80 | 2.70 | 2.70 |
| FUM | 1.70 | 3.30 | 2.20 |
| FDA | 1.78 | 2.97 | 2.25 |
| DUD | 1.66 | 3.00 | 1.66 |
| BB9 | 2.12 | 2.90 | 2.45 |
| WO2 | 3.20 | 4.50 | 4.50 |
| STU | 1.75 | 3.43 | 2.80 |
| PER | 1.50 | 3.19 | 2.32 |
| OZT | 2.43 | 2.60 | 2.43 |
| L3P | 1.80 | 2.60 | 2.60 |
| FOR | 1.40 | 2.70 | 1.40 |
| FA1 | 2.00 | 3.10 | 3.10 |
| DHA | 1.20 | 2.90 | 1.82 |
| V7O | 2.80 | 2.90 | 2.80 |
| SPD | 1.65 | 3.20 | 1.95 |
| PSC | 1.80 | 2.70 | 1.90 |
| ORO | 1.55 | 3.00 | 3.00 |
| CH2 | 1.66 | 3.35 | 3.00 |
| PG6 | 1.40 | 2.50 | 1.65 |
| PPV | 1.60 | 3.20 | 2.90 |
| PLR | 1.60 | 3.00 | 2.10 |
| 5RP | 1.55 | 2.90 | 2.79 |
| PPU | 2.20 | 3.70 | 2.95 |
| DTY | 1.40 | 2.80 | 1.66 |
| CAM | 1.40 | 2.90 | 1.80 |
| ASN | 1.50 | 3.00 | 2.00 |
| PNS | 1.50 | 2.55 | 2.15 |
| NIY | 1.60 | 3.10 | 2.75 |
| FPP | 1.30 | 3.40 | 2.70 |
| CUA | 1.60 | 4.80 | 2.80 |
| BGL | 2.10 | 3.20 | 3.20 |
| REA | 1.80 | 3.20 | 2.96 |
| LYZ | 1.63 | 5.16 | 5.16 |
| ICT | 1.60 | 3.20 | 1.60 |
| FOL | 1.44 | 3.26 | 1.80 |
| DTU | 1.20 | 2.95 | 1.80 |
| BCN | 1.43 | 2.90 | 2.25 |
| BCD | 1.49 | 3.30 | 2.10 |
| AG2 | 1.40 | 3.10 | 2.00 |
| TP7 | 1.16 | 2.70 | 1.45 |
| OXM | 1.60 | 2.70 | 2.20 |
| CCN | 0.90 | 2.90 | 2.90 |
| AGM | 1.16 | 1.90 | 1.45 |
| EMC | 1.69 | 3.30 | 2.60 |
| CXP | 2.40 | 2.60 | 2.60 |
| 2MG | 1.93 | 17.00 | 3.00 |
| R5P | 1.59 | 3.10 | 2.50 |
| PG0 | 1.39 | 2.80 | 1.70 |
| PEH | 2.20 | 3.00 | 3.00 |
| HAS | 2.00 | 4.80 | 3.30 |
| FNR | 1.50 | 2.90 | 2.29 |
| AZM | 1.50 | 2.96 | 1.70 |
| AYA | 1.37 | 3.50 | 1.37 |
| ADX | 1.43 | 2.81 | 2.49 |
| NMN | 1.50 | 2.90 | 1.90 |
| BAL | 1.80 | 3.10 | 2.00 |
| SCY | 1.60 | 2.70 | 1.60 |
| R1P | 1.44 | 2.40 | 2.40 |
| PXX | 2.01 | 3.10 | 2.01 |
| PUT | 1.50 | 2.62 | 1.84 |
| MLK | 2.32 | 2.90 | 2.90 |
| PTY | 1.60 | 3.15 | 2.20 |
| DAN | 1.50 | 3.50 | 2.10 |
| D10 | 1.60 | 2.80 | 2.20 |
| API | 1.41 | 2.62 | 2.00 |
| NCA | 1.40 | 3.00 | 2.05 |
| FGA | 1.66 | 2.80 | 2.06 |
| A2P | 1.70 | 3.10 | 3.00 |
| YOF | 1.85 | 2.20 | 1.85 |
| SPM | 1.66 | 3.20 | 1.83 |
| PID | 1.40 | 2.10 | 1.95 |
| NCN | 1.60 | 3.31 | 2.65 |
| MA4 | 1.24 | 2.02 | 1.30 |
| FDP | 1.50 | 3.00 | 2.03 |
| 78M | 2.30 | 3.30 | 2.60 |
| IAS | 1.60 | 2.80 | 2.70 |
| ETF | 1.45 | 2.80 | 1.80 |
| ABU | 1.28 | 2.90 | 1.44 |
| YG | 2.00 | 17.00 | 3.35 |
| OPE | 1.45 | 12.50 | 2.30 |
| MQ7 | 1.86 | 8.20 | 1.86 |
| H2S | 1.59 | 3.00 | 2.00 |
| CP | 1.87 | 2.90 | 1.90 |
| SLB | 1.40 | 2.60 | 2.20 |
| RNS | 1.58 | 2.10 | 1.58 |
| MOS | 1.60 | 3.40 | 2.20 |
| DHE | 1.28 | 2.90 | 1.60 |
| DCM | 1.60 | 3.40 | 2.30 |
| DAR | 1.25 | 2.75 | 2.75 |
| 5CM | 1.60 | 2.89 | 1.99 |
| XYL | 1.60 | 3.30 | 3.30 |
| SVR | 1.70 | 2.50 | 2.06 |
| SOG | 1.80 | 3.65 | 3.00 |
| O12 | 1.70 | 1.70 | 1.70 |
| MOO | 1.50 | 2.75 | 2.30 |
| DXC | 1.67 | 3.85 | 1.90 |
| GER | 1.50 | 3.00 | 2.60 |
| BE2 | 1.50 | 2.60 | 2.40 |
| AP5 | 1.58 | 3.50 | 1.80 |
| ACH | 1.80 | 3.10 | 1.80 |
| STL | 1.20 | 3.20 | 2.33 |
| OS | 2.00 | 3.85 | 2.90 |
| LU | 1.60 | 3.00 | 2.20 |
| HPA | 1.75 | 3.40 | 2.70 |
| G3H | 1.50 | 3.00 | 2.25 |
| G2P | 1.80 | 23.00 | 4.70 |
| B3N | 1.80 | 3.20 | 1.85 |
| SGM | 1.40 | 2.70 | 2.00 |
| PMS | 1.40 | 2.60 | 2.60 |
| PHI | 1.70 | 2.84 | 2.31 |
| P5P | 2.00 | 3.60 | 3.40 |
| MHO | 1.22 | 2.30 | 1.76 |
| IHP | 1.50 | 2.90 | 2.60 |
| EPH | 1.65 | 11.60 | 3.01 |
| CMH | 1.69 | 1.69 | 1.69 |
| C2F | 1.85 | 3.50 | 2.55 |
| AMG | 1.90 | 3.30 | 2.80 |
| A5P | 1.70 | 2.10 | 1.95 |
| URF | 1.80 | 2.60 | 1.80 |
| TMO | 1.68 | 3.10 | 1.75 |
| TCA | 1.90 | 3.30 | 1.90 |
| NEP | 1.45 | 2.90 | 1.50 |
| RUB | 2.15 | 21.00 | 21.00 |
| NVP | 2.09 | 5.00 | 2.50 |
| NCT | 2.20 | 2.85 | 2.20 |
| GCO | 1.55 | 2.50 | 2.19 |
| G39 | 1.75 | 2.95 | 2.20 |
| ETX | 1.50 | 2.50 | 2.45 |
| DY | 2.31 | 2.31 | 2.31 |
| CBS | 1.58 | 3.30 | 2.60 |
| C2O | 1.50 | 2.70 | 2.20 |
| ACN | 1.20 | 2.80 | 1.75 |
| ACM | 1.54 | 2.70 | 2.30 |
| 6MZ | 1.60 | 2.70 | 1.80 |
| TBG | 1.73 | 3.00 | 2.75 |
| TB | 1.80 | 2.90 | 2.10 |
| SPO | 1.80 | 3.50 | 2.90 |
| QUE | 1.50 | 3.20 | 2.40 |
| PGR | 1.50 | 2.30 | 1.70 |
| L2P | 1.80 | 2.70 | 2.50 |
| IIL | 1.50 | 2.00 | 2.00 |
| IBM | 1.50 | 11.00 | 2.10 |
| Y5P | 3.40 | 3.60 | 3.60 |
| T44 | 1.10 | 3.10 | 3.00 |
| SPH | 1.85 | 13.00 | 2.00 |
| S6P | 1.50 | 2.82 | 2.58 |
| RJP | 1.90 | 1.90 | 1.90 |
| PBZ | 1.55 | 3.00 | 1.95 |
| OMT | 1.45 | 3.00 | 2.70 |
| NIO | 1.36 | 2.40 | 2.00 |
| N87 | 2.40 | 2.40 | 2.40 |
| MHS | 1.16 | 2.30 | 1.30 |
| M2G | 1.93 | 17.00 | 3.00 |
| FPS | 1.66 | 2.82 | 2.25 |
| FEC | 1.95 | 2.35 | 2.35 |
| D12 | 1.60 | 3.23 | 2.20 |
| A23 | 1.99 | 3.15 | 2.90 |
| 6PG | 1.50 | 2.50 | 2.40 |
| PCG | 1.90 | 2.90 | 2.10 |
| PAR | 1.80 | 3.50 | 2.00 |
| INS | 1.42 | 2.80 | 2.65 |
| FTT | 2.30 | 3.30 | 3.10 |
| FMC | 1.60 | 2.60 | 1.70 |
| CPT | 1.42 | 3.16 | 1.97 |
| XMP | 1.60 | 2.72 | 1.60 |
| OAS | 2.03 | 3.20 | 2.69 |
| GL3 | 1.16 | 2.50 | 1.45 |
| CIR | 1.80 | 2.90 | 2.30 |
| BEM | 1.50 | 2.21 | 1.55 |
| Y01 | 2.50 | 3.36 | 2.70 |
| UGA | 1.70 | 3.00 | 2.10 |
| THJ | 1.63 | 2.50 | 2.10 |
| S9L | 2.05 | 2.90 | 2.65 |
| RU | 1.50 | 2.65 | 1.82 |
| PQQ | 1.44 | 3.00 | 2.30 |
| NMY | 1.70 | 2.95 | 2.40 |
| HMR | 1.80 | 3.50 | 2.10 |
| EST | 1.60 | 3.20 | 2.30 |
| CY3 | 1.50 | 2.07 | 1.60 |
| BB2 | 1.50 | 3.30 | 1.50 |
| ALQ | 1.60 | 3.39 | 2.50 |
| 3FG | 1.70 | 2.80 | 2.15 |
| UQ2 | 2.04 | 3.00 | 2.04 |
| TYI | 1.39 | 2.90 | 2.00 |
| TBR | 1.70 | 3.94 | 1.80 |
| SSU | 2.50 | 3.50 | 2.60 |
| SC2 | 1.95 | 2.80 | 2.80 |
| PPI | 1.45 | 2.50 | 2.40 |
| PAL | 1.80 | 2.80 | 1.80 |
| P77 | 2.80 | 2.80 | 2.80 |
| MBO | 1.40 | 2.40 | 1.75 |
| LI | 1.46 | 2.80 | 1.70 |
| KIW | 2.57 | 2.57 | 2.57 |
| KIU | 2.40 | 2.40 | 2.40 |
| ILE | 1.75 | 3.20 | 1.95 |
| DBB | 1.60 | 3.42 | 2.31 |
| CAQ | 1.55 | 2.70 | 2.70 |
| BMQ | 1.35 | 2.50 | 1.35 |
| 1R2 | 2.80 | 2.80 | 2.80 |
| UPL | 1.90 | 1.90 | 1.90 |
| STE | 1.37 | 2.70 | 2.30 |
| PE3 | 1.83 | 3.20 | 1.95 |
| MO | 1.70 | 3.20 | 2.20 |
| MK8 | 1.70 | 4.20 | 2.57 |
| DCS | 1.90 | 2.71 | 2.30 |
| CTT | 1.60 | 2.46 | 2.01 |
| CAF | 1.70 | 3.15 | 1.95 |
| YCP | 2.60 | 2.60 | 2.60 |
| SME | 1.30 | 2.30 | 2.30 |
| M1N | 2.99 | 2.99 | 2.99 |
| KAI | 1.49 | 3.40 | 1.97 |
| HNI | 1.45 | 2.81 | 1.45 |
| FUA | 2.18 | 3.05 | 2.20 |
| DPO | 1.53 | 3.30 | 3.30 |
| DDQ | 1.65 | 2.99 | 2.10 |
| AMU | 1.65 | 2.50 | 2.30 |
| 3PO | 1.11 | 3.70 | 2.20 |
| 39Y | 3.20 | 3.20 | 3.20 |
| XLS | 1.60 | 2.50 | 2.30 |
| SGC | 1.44 | 2.00 | 1.90 |
| SCH | 1.70 | 2.41 | 1.71 |
| MN3 | 1.45 | 2.75 | 2.56 |
| LVS | 3.10 | 3.20 | 3.20 |
| GXL | 1.50 | 3.01 | 3.01 |
| DMN | 2.00 | 2.30 | 2.00 |
| DHT | 1.40 | 2.79 | 2.03 |
| BLM | 1.40 | 2.80 | 1.50 |
| XRX | 2.48 | 2.48 | 2.48 |
| THR | 1.58 | 2.80 | 1.58 |
| NET | 1.80 | 2.10 | 2.00 |
| N8E | 2.40 | 3.50 | 3.00 |
| MTN | 1.97 | 2.50 | 2.50 |
| LMD | 2.99 | 3.19 | 3.19 |
| IMH | 1.40 | 2.90 | 2.20 |
| FEB | 2.50 | 2.60 | 2.50 |
| DPP | 1.90 | 2.59 | 1.93 |
| DCL | 1.20 | 2.80 | 1.85 |
| AHG | 2.10 | 3.00 | 2.10 |
| ACA | 1.40 | 2.95 | 1.80 |
| 4LJ | 3.39 | 3.39 | 3.39 |
| 4LE | 3.00 | 3.39 | 3.39 |
| 1PS | 1.50 | 1.80 | 1.80 |
| TAC | 1.62 | 4.50 | 2.80 |
| PFF | 1.85 | 2.90 | 2.90 |
| NPO | 1.20 | 2.50 | 1.81 |
| NGT | 1.61 | 3.25 | 2.40 |
| MYA | 1.30 | 2.40 | 2.08 |
| GTS | 1.37 | 2.90 | 1.90 |
| GTB | 1.40 | 3.00 | 2.50 |
| GMP | 1.55 | 3.20 | 1.70 |
| GLV | 1.60 | 3.00 | 1.75 |
| BGM | 1.50 | 2.68 | 2.49 |
| BDP | 1.45 | 2.70 | 2.70 |
| 22B | 1.80 | 2.70 | 2.20 |
| OEC | 3.00 | 6.56 | 3.60 |
| KDA | 1.45 | 2.85 | 2.60 |
| IYR | 1.70 | 2.50 | 1.77 |
| HXC | 1.75 | 2.50 | 2.10 |
| GOA | 1.55 | 3.00 | 2.60 |
| GMH | 1.70 | 3.30 | 3.30 |
| FU2 | 2.00 | 2.30 | 2.30 |
| DND | 1.70 | 3.20 | 1.90 |
| DDG | 1.62 | 3.30 | 2.75 |
| CO2 | 1.56 | 2.80 | 1.60 |
| ANU | 1.74 | 2.38 | 1.74 |
| 3PH | 1.90 | 3.04 | 2.50 |
| TOP | 1.53 | 2.89 | 2.00 |
| PAU | 1.51 | 2.70 | 2.50 |
| NMG | 2.00 | 2.00 | 2.00 |
| NME | 1.65 | 3.00 | 1.85 |
| NHW | 1.38 | 2.33 | 1.58 |
| MLC | 1.45 | 3.10 | 3.10 |
| HSO | 1.65 | 2.80 | 2.36 |
| FON | 1.40 | 3.20 | 1.40 |
| C | 1.80 | 2.85 | 2.30 |
| ACR | 1.40 | 2.95 | 2.05 |
| 4CO | 1.50 | 1.80 | 1.80 |
| 0FQ | 1.62 | 1.89 | 1.85 |
| XCC | 1.90 | 2.51 | 2.51 |
| VIB | 1.80 | 3.05 | 2.60 |
| TSA | 1.64 | 3.00 | 1.64 |
| SOR | 1.60 | 2.85 | 2.30 |
| PPY | 1.50 | 2.90 | 2.22 |
| PAM | 1.71 | 3.25 | 1.90 |
| OEX | 1.90 | 5.50 | 4.90 |
| NIT | 1.70 | 2.80 | 2.00 |
| NAA | 1.70 | 2.80 | 1.85 |
| MBN | 1.56 | 3.30 | 1.85 |
| IM5 | 1.70 | 2.30 | 1.70 |
| HCS | 1.70 | 2.65 | 1.79 |
| GDX | 1.55 | 2.80 | 1.55 |
| DCT | 1.58 | 3.60 | 2.30 |
| ARM | 1.75 | 3.65 | 2.00 |
| AHZ | 1.60 | 2.70 | 1.82 |
| 16G | 1.50 | 2.40 | 2.20 |
| 00S | 2.15 | 2.70 | 2.70 |
| UVW | 1.45 | 2.85 | 1.60 |
| URC | 1.50 | 2.30 | 1.75 |
| UQ | 2.10 | 3.51 | 3.00 |
| SKM | 1.40 | 2.90 | 1.98 |
| RMN | 1.85 | 2.80 | 1.85 |
| PHD | 1.58 | 4.60 | 2.56 |
| PC | 1.82 | 3.10 | 2.38 |
| LAC | 1.00 | 2.50 | 2.30 |
| IMN | 1.40 | 2.90 | 1.95 |
| H4M | 1.80 | 2.15 | 1.80 |
| CCC | 1.90 | 3.45 | 2.50 |
| A2M | 2.19 | 2.65 | 2.25 |
| 2PG | 1.40 | 2.65 | 1.80 |
| UQ1 | 1.96 | 19.00 | 3.70 |
| UOQ | 1.90 | 1.90 | 1.90 |
| SNN | 1.08 | 2.40 | 2.10 |
| OMY | 0.89 | 2.50 | 1.90 |
| O4B | 1.45 | 2.60 | 1.45 |
| MRY | 1.80 | 3.10 | 2.76 |
| DHI | 1.45 | 2.91 | 2.00 |
| CE1 | 2.30 | 3.60 | 3.30 |
| BFD | 1.50 | 3.10 | 2.15 |
| AS1 | 1.96 | 2.30 | 1.96 |
| AAL | 1.59 | 2.30 | 1.59 |
| XY1 | 2.10 | 2.43 | 2.10 |
| TFA | 1.26 | 2.50 | 2.05 |
| STR | 1.59 | 3.00 | 1.80 |
| S4M | 1.58 | 2.20 | 1.60 |
| PHT | 1.81 | 2.55 | 2.29 |
| PCP | 2.00 | 2.90 | 2.22 |
| PCI | 1.80 | 3.20 | 2.50 |
| NTM | 2.30 | 3.09 | 2.30 |
| NLG | 1.50 | 3.46 | 3.46 |
| LBT | 1.60 | 2.65 | 2.27 |
| G3P | 1.50 | 3.00 | 2.50 |
| FFO | 1.90 | 2.70 | 2.10 |
| FAH | 1.20 | 2.00 | 1.76 |
| CYT | 1.75 | 3.00 | 3.00 |
| BAU | 1.54 | 2.20 | 2.00 |
| A3S | 1.48 | 2.25 | 1.86 |
| 5PA | 2.00 | 2.70 | 2.20 |
| SN0 | 2.30 | 2.90 | 2.50 |
| RIP | 1.60 | 2.50 | 2.15 |
| RIO | 1.50 | 2.20 | 2.20 |
| PSJ | 1.60 | 2.08 | 1.79 |
| PIN | 1.50 | 2.50 | 2.25 |
| OSF | 2.70 | 2.70 | 2.70 |
| NFA | 1.55 | 2.70 | 2.20 |
| MH9 | 2.80 | 3.00 | 2.80 |
| LWY | 2.81 | 2.81 | 2.81 |
| G1P | 1.60 | 2.80 | 2.20 |
| FTR | 1.60 | 2.30 | 2.30 |
| DMT | 2.10 | 2.20 | 2.10 |
| DFP | 1.40 | 3.60 | 1.40 |
| CMQ | 1.90 | 1.90 | 1.90 |
| BXB | 1.69 | 1.86 | 1.86 |
| BML | 1.72 | 2.30 | 1.72 |
| BET | 1.59 | 3.35 | 1.90 |
| ASJ | 1.70 | 3.00 | 1.70 |
| AES | 1.70 | 3.43 | 1.80 |
| 0YR | 1.80 | 1.80 | 1.80 |
| 0VJ | 2.59 | 2.59 | 2.59 |
| 09T | 2.50 | 2.50 | 2.50 |
| XPC | 2.00 | 2.80 | 2.80 |
| PQ9 | 3.00 | 4.00 | 3.70 |
| PDC | 1.97 | 2.60 | 2.50 |
| MIS | 1.34 | 3.00 | 3.00 |
| KR | 1.40 | 2.20 | 1.90 |
| GDN | 1.85 | 3.50 | 3.00 |
| FCO | 1.50 | 2.70 | 2.04 |
| CNC | 1.60 | 3.10 | 3.10 |
